# Cell size regulates human endoderm specification through actomyosin-dependent AMOT-YAP signaling

**DOI:** 10.1016/j.stemcr.2024.07.001

**Published:** 2024-08-01

**Authors:** Lai Jiang, Chenchao Yan, Ying Yi, Lihang Zhu, Zheng Liu, Donghui Zhang, Wei Jiang

**Affiliations:** 1 State Key Laboratory of Biocatalysis and Enzyme Engineering, School of Life Science, Hubei University, Wuhan 430062, China; 2 Department of Biological Repositories, Frontier Science Center for Immunology and Metabolism, Medical Research Institute, Zhongnan Hospital of Wuhan University, Wuhan University, Wuhan 430071, China; 3 The Institute for Advanced Studies, Wuhan University, Wuhan, China; 4 Hubei Provincial Key Laboratory of Developmentally Originated Disease, Wuhan, China

**Keywords:** human endoderm differentiation, cell size, osmotic pressure, actomyosin, YAP, AMOT, pluripotent stem cell

## Abstract

Cell size is a crucial physical property that significantly impacts cellular physiology and function. However, the influence of cell size on stem cell specification remains largely unknown. Here, we investigated the dynamic changes in cell size during the differentiation of human pluripotent stem cells into definitive endoderm (DE). Interestingly, cell size exhibited a gradual decrease as DE differentiation progressed with higher stiffness. Furthermore, the application of hypertonic pressure or chemical to accelerate the reduction in cell size significantly and specifically enhanced DE differentiation. By functionally intervening in mechanosensitive elements, we have identified actomyosin activity as a crucial mediator of both DE differentiation and cell size reduction. Mechanistically, the reduction in cell size induces actomyosin-dependent angiomotin (AMOT) nuclear translocation, which suppresses Yes-associated protein (YAP) activity and thus facilitates DE differentiation. Together, our study has established a novel connection between cell size diminution and DE differentiation, which is mediated by AMOT nuclear translocation. Additionally, our findings suggest that the application of osmotic pressure can effectively promote human endodermal lineage differentiation.

## Highlights

• Cell size decreases during the differentiation of human pluripotent stem cells into endoderm
• Hypertonic pressure is conducive to the differentiation of human definitive endoderm
• Actomyosin contributes to both size diminution and endoderm promotion under hypertonic pressure
• Cell size diminution represses YAP activity via promoting AMOT nuclear translocation

## Graphical abstract

**Figure:**
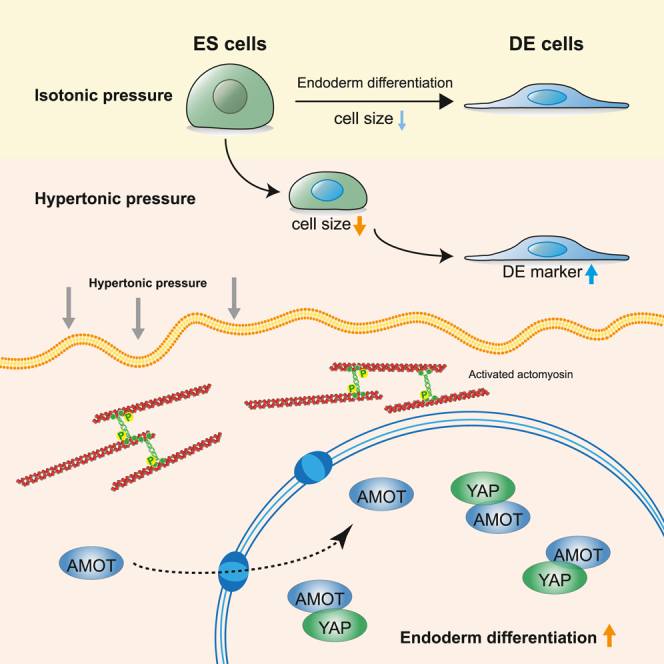

## Teaser

Jiang and colleagues show that cell size exhibits a gradual decrease during human endoderm differentiation. The application of hypertonic pressure or chemical to accelerate the reduction in cell size significantly and specifically enhanced endoderm differentiation. This enhancement is reliant on actomyosin activity and achieved by promoting the nuclear translocation of AMOT, thereby repressing YAP activity.

## Introduction

Cell size is a key characteristic of cell state that undergoes changes throughout the cell’s lifespan, such as enlarging during cell senescence (Lengefeld et al., 2021) and cell growth (Fingar et al., 2002). Furthermore, cell size can change rapidly on shorter time scales, for example, during cell cycle (Cadart et al., 2022) and cell spreading (Guo et al., 2017). Importantly, reducing the size of large cells can rescue their senescence, while inhibiting cell enlargement can suppress cell cycle (Lengefeld et al., 2021; Cadart et al., 2022), indicating that cell size actively regulates multiple biological functions. Cell size is intrinsically related to the mechanical state and physical properties of cells. External control of cell size can be achieved through physical stimuli, such as osmotic pressure and substrate stiffness, as well as chemicals, including various ion channel regulators (Liu et al., 2020; Venkova et al., 2022). Cell shrinkage leads to membrane folding, reducing cell plasma membrane tension and cell elasticity with increasing Young’s modulus (Guo et al., 2017; Roffay et al., 2021). In addition, cell size diminution could induce phase-separated biomolecular condensates through molecular crowding, as reported in regulating WNT and HIPPO pathways (Li et al., 2021b; Wang et al., 2022). Interestingly, different cell types from the same organism can exhibit significantly different volumes. For example, pancreatic β cells are surrounded by acinar cells that are roughly twice their size (Auffret et al., 2013), and this disparity can be utilized as a strategy for cell sorting (Smejkal et al., 2023).

Cell volume undergoes changes during embryonic development and organogenesis. The oocyte undergoes maturation, accompanied by increased osmotic pressure and activation of intracellular calcium signals, during its transition from the ovary to the uterus (Horner and Wolfner, 2008). In the earliest preimplantation embryos, cell size continuously decreases from the zygote to the blastocysts. During this stage, the embryonic cells divide without increasing in volume (Courtois et al., 2012). In the blastocyst stage, cells actively create an osmotic gradient to generate luminal pressure. This pressure stretches and compresses trophectoderm cells, thereby determining their fate (Chan et al., 2019). In addition, neurogenesis from neural stem cells is strongly promoted in soft extracellular matrix with large cell size but suppressed by cell size compression (Baek et al., 2022); intestinal stem cells have small cell size and conical shape, distinct from their differentiating derivates. Interestingly, these unique cellular characteristics activate the WNT signaling pathway by regulating molecular crowding and niche interactions, further enhancing their stemness (Li et al., 2021b; Pentinmikko et al., 2022). These findings raise the hypothesis that cell size might actively participate in cell fate determination. However, it is still unknown whether cell size changes and contributes to human early embryonic germ layer differentiation. Human embryonic stem cells (ESCs) are derived from human blastocysts and can self-renew indefinitely *in vitro* while retaining the potential to differentiate into the three germ layers (Thomson et al., 1998). Therefore, the *in vitro* differentiation of human ESCs provides a valuable tool to investigate the functional relationship between cell size and early germ layer differentiation.

In this study, we utilized the directed definitive endoderm (DE) differentiation system, which gives rise to respiratory and digestive epithelium, as well as the thyroid, thymus, liver, and pancreas (Wells and Melton, 1999), to investigate the cell size dynamics and dissect the functional link between cell size and differentiation. Our findings revealed that during DE differentiation from human ESCs, there was a decrease in cell size, and applying hypertonic pressure or chemical to induce cell compression promoted DE differentiation. In addition, we observed that the mechanosensitive actomyosin played a role in hypertonic cell compression and mediated the boosted DE differentiation. Furthermore, we explored the underlying mechanism and identified that Yes-associated protein (YAP)-angiomotin (AMOT) co-localization under hypertonic pressure contributed to DE differentiation by suppressing the YAP pathway.

## Results

### DE differentiation is accompanied by a diminution of cell size and change in cell mechanical state

**Figure 1:**
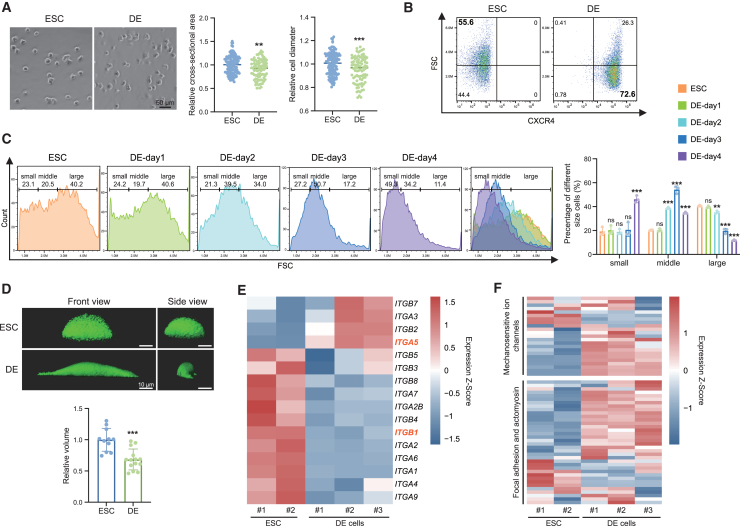
Human definitive endoderm differentiation is accompanied by changes in cell size and cell mechanical state (A) Representative images of suspended ESCs and DE cells and quantification of their cross-sectional area and diameter (*n* > 85 cells from 3 independent batches per group analyzed, scale bar is 50 μm). (B) Flow cytometric analysis of CXCR4 in ESCs and DE cells. Size distribution of cells as determined by forward scatter (FSC). (C) Flow cytometric analysis of the time-course size distribution of ESCs and DE cells as determined by FSC. Gates of small, middle, and large cell size and time-course (day 0–4) quantification of gates percentage during endoderm differentiation are indicated (*n* = 3 from 3 independent batches). (D) Representative 3D confocal images of GFP-labeled ESCs and DE cells, and quantification of their volume (*n* > 11 cells from 3 independent batches per group analyzed, scale bar is 10 μm). (E and F) Heatmap showing the expression of cell volume and cell mechanical state-related genes within ESCs and DE. (E) Heatmap showing the higher expression of *ITGA5* in DE cells, which marks cell high mechanical state; the lower expression of *ITGB1* in DE cells, which participates in cell response to soft matrix (ns means not statistically significant, ^∗∗^*p* < 0.01, ^∗∗∗^*p* < 0.001).

Since it has not yet been determined whether cell fate commitment during embryonic germ layer development is associated with changes in cell size, we utilized DE differentiation of human ESCs to investigate the dynamics of cell size. We initially examined the size of ESCs and differentiated DE cells under microscopy and observed a decrease in the cell size after endoderm differentiation (Figure 1A). The result from Coulter counter also clearly showed that DE cells exhibited smaller cell size than ESCs in suspension condition (Figure S1A). Since the forward scatter (FSC) value determined by the flow cytometer was able to reflect the cell size (Pentinmikko et al., 2022), we performed the same assay and observed that the size of DE cells was significantly smaller than that of ESCs (Figure 1B). We further analyzed the time-course size distribution by categorizing cells into small, middle, and large groups based on FSC gating. The results revealed a gradual increase in the proportion of cells with small size and a decrease in the proportion of cells with large size (Figure 1C), indicating a decrease in cell size over time during DE induction. In addition, we calculated the cell volume and surface area by segmenting time-lapse three-dimensional (3D) confocal stacks of GFP-labeled cells. The result showed that DE cells were statistically significantly smaller compared to ESCs (Figure 1D).

ESCs with a high proliferation rate exhibit increased G2/M phase, likely contributing to the larger size. To investigate this possibility arising from differences in the cell cycle phases, we compared the cell size of DE and ESC in the same state. We indeed observed a significant difference in the cell cycle phases between ESCs and DE cells, with ESCs exhibiting a higher proportion of G2/M phase compared to DE cells (Figure S1B). However, the size of DE cells during the G0/G1 and G2/M phases was smaller than that of ESCs in the corresponding phases (Figure S1B). These data demonstrate that the existence of a cell size difference between ESCs and DE cells cannot be attributed to disparities in the cell cycle states. Additionally, we investigated the alterations in cell size during mesoderm and ectoderm differentiation as a control for our endoderm differentiation. All three cell types were derived from human ESCs and exhibited lower proliferation rates compared to ESCs. Our data indicated that the cell size of mesoderm was slightly smaller than that of ESCs, while the cell size of ectoderm was significantly larger (Figure S1C). These variations in cell size among endoderm, mesoderm, and ectoderm could also support that the varying proliferation rates are not the major cause, at least, of cell size change during lineage differentiation.

Cell size is intrinsically related to cell mechanical state, including cell stiffness, membrane tension, and other parameters such as molecular crowding (Guo et al., 2017; Li et al., 2021b). Additionally, mechanosensitive ion channels are associated with cell susceptibility to various forms of mechanical forces, as well as the processes of cell-autonomous osmotic pressure regulation and cell volume change (Fang et al., 2021). Thus, we first examined the expression of integrin, focal adhesion, actomyosin cytoskeleton and mechanosensitive ion channel-related genes in ESCs and DE cells (Figures 1E and 1F). We found that ESCs and DE cells expressed different types of integrin, indicating that they exhibited different mechanical states. Integrin β1 has been reported to participate in the cell’s response to a soft matrix and it was highly expressed in ESCs. In contrast, α5 integrin signaling is known to monitor cells with a high mechanical state (Lv et al., 2015), which showed higher expression in DE cells (Figure 1E). Meanwhile, the expression levels of most focal adhesion, actomyosin and mechanosensitive ion channel-related genes were significantly different between ESCs and DE cells (Figure 1F). To further assess the difference of integrin tension between ESC and DE cells, we used the reversible shearing DNA-based tension probe (Li et al., 2021a). The results indicated that the tension signal in ESCs was diffuse and their fluorescence intensities of 56-pN were significantly lower. In DE cells, the tension signal was localized at a large area of cell edge and a clear 56-pN tension signal was observed (Figure S1D). The quantification of the 56-pN/12-pN tension signal indicated that the integrin tension to the substrate in DE cells is much stronger than ESCs. Overall, these observations demonstrate that human DE differentiation is accompanied by a decrease in cell size, a specific mechanical state with stronger mechanical tension, and an increase in cell stiffness.

### Cell size diminution contributes to endoderm differentiation

To address whether smaller cells are more competent to endoderm differentiation, we manipulated cell size externally using hypertonic pressure. Hypertonic pressure treatment rapidly and consistently reduces cell volume within a few seconds and for the subsequent period (Guo et al., 2017; Lee et al., 2019; Li et al., 2021b; Roffay et al., 2021). We added sucrose or PEG300 to the medium as hypertonic pressure for 48 h and observed a significant decrease in the volume of ESCs with increasing hypertonicity (Figures S2A–S2C). We also observed that GFP-labeled ESCs presented obviously smaller cell size after 30 min of osmotic pressure stimulation (Figure S2D). The results demonstrate that osmotic pressure is an effective and stable way to regulate cell volume as expected (Guo et al., 2017; Lee et al., 2019; Li et al., 2021b; Roffay et al., 2021). Furthermore, under hypertonic treatment, human ESCs maintained good colony morphology and displayed positive alkaline phosphatase staining (Figure S2E).

**Figure 2:**
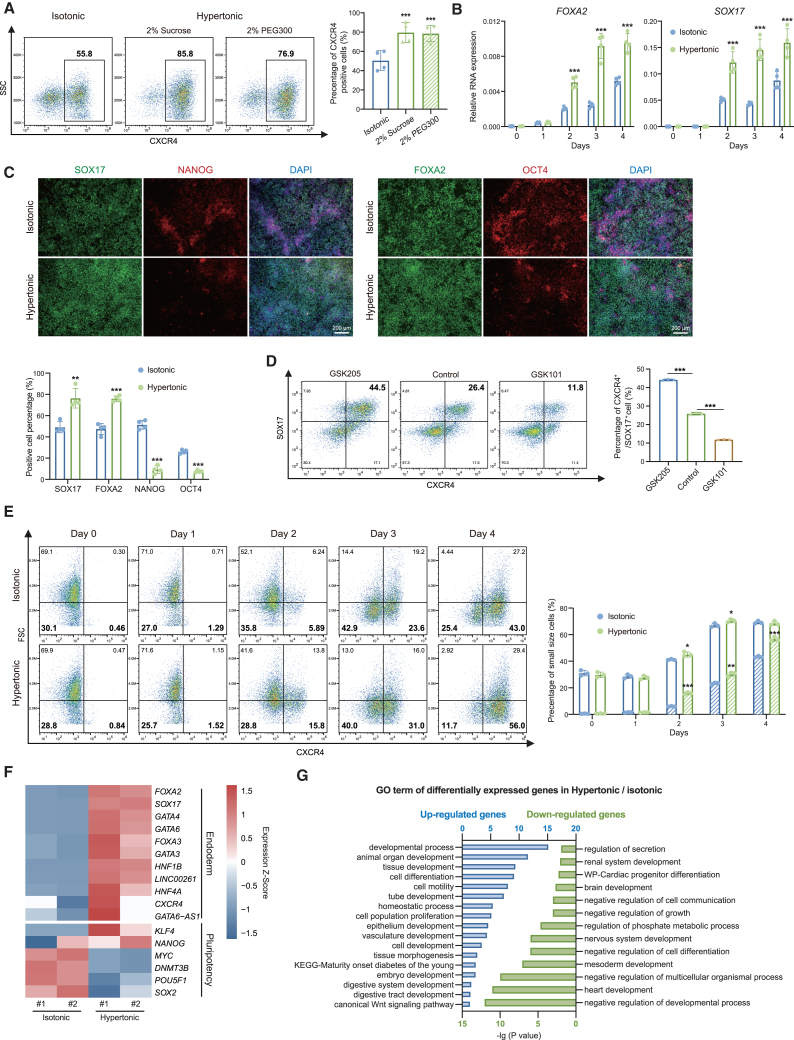
Cell size diminution promotes endoderm differentiation (A) Flow cytometric analysis of CXCR4 expression under isotonic or hypertonic DE differentiation condition and quantification of the DE differentiation efficiency marked as CXCR4-positive cells (*n* = 4 from 4 independent batches). (B) Time-course analysis of mRNA expression of DE markers under isotonic or hypertonic differentiation condition (*n* = 4 from 4 independent batches). (C) Immunostaining analysis of isotonic or hypertonic differentiated DE cells: DE markers SOX17 and FOXA2 (green); ESC markers: NANOG and OCT4 (red); and DNA (DAPI; blue) (scale bar is 200 μm). Quantifications of the positive staining shown as left-below. (D) Intracellular flow cytometric analysis of CXCR4 and SOX17 of differentiated DE cells treated with TRPV4 antagonist GSK205, or agonist GSK101. Quantification was shown as right (*n* = 3 from 3 independent batches). (E) Time-course analysis of cell size distribution during DE differentiation process (*n* = 3 from 3 independent batches). CXCR4-positive cells were used to mark DE cells and lower FSC marks small cells. Quantifications of small size cell percentage shown as right: hollow pillars indicate all the small size cells and filled pillars indicate the CXCR4-positive small size cells. (F) The heatmap showed the differentially expressed DE and ESC marker genes in isotonic and hypertonic DE cells. (G) Gene ontology analysis of upregulated (blue) and downregulated (green) genes in DE cells responding to hypertonic pressure (ns means not statistically significant, ^∗^*p* < 0.05, ^∗∗^*p* < 0.01, ^∗∗∗^*p* < 0.001).

Next, we performed DE differentiation under hypertonic condition and observed a significant increase in the percentage of CXCR4-positive DE cells compared to unperturbed DE cells in isotonic medium (Figure 2A). Moreover, the efficiency of DE differentiation increased with the degree of hypertonicity (Figures S2F and S2G). This was further supported by the analysis of the expression of endoderm markers, forkhead box A2 (*FOXA2*) and SRY-box transcription factor 17 (*SOX17*) (Figures 2B and 2C). Importantly, this phenomenon was observed in suspension differentiation systems as well (Figures S2H and S2I), and not impacted by the addition of PI3K inhibitor LY294002 or agonist insulin-containing medium (Figures S3A and S3B). Of note, the hypertonic pressure used in this study did not impact the apoptosis or cell cycle during DE differentiation (Figures S3C and S3D).

Moreover, we employed another approach to manipulate cell size, in addition to hypertonic treatment, and evaluated the DE differentiation. The transient receptor potential vanilloid-4 (TRPV4) ion channels regulate cell volume through balancing osmolality of calcium ions in the cytoplasm (Jo et al., 2015). The protein level of TRPV4 in MSCs was found to be diminished when cell volume expansion was restricted, such as increased osmotic pressure (Lee et al., 2019). By analyzing our RNA sequencing (RNA-seq) data (Jiang et al., 2015; Lu et al., 2023), we found TRPV4 was lower expressed in DE samples than in ESCs (Figure S3E), suggesting that TRPV4 might contribute to the cell volume change during DE differentiation. Since the TRPV4 agonist GSK101 increased the volume of cells, while cell volume expansion was significantly restricted by treatment with the TRPV4 antagonist GSK205 (Lee et al., 2019), we therefore applied the TRPV4 agonist and antagonist during DE differentiation of human ESCs. As expected, we found that the agonist GSK101 could efficiently enlarge cell volume, while the antagonist GSK205 could decrease cell size (Figure S3F). Most importantly, GSK205 enhanced DE induction and GSK101 reduced DE induction, measured by flow cytometric analysis of CXCR4 and SOX17 (Figure 2D). These data together with the hypertonic treatment support that cell size plays an important role in DE differentiation.

Next, we performed the time-course analysis of cell size distribution, showing that hypertonic pressure increased the percentage of cells with small size during endoderm differentiation, and CXCR4-positive cells appeared to have a more pronounced reduction in cell size (Figure 2E). Since the most significant reduction in cell size occurred between day 2 and day 3, we modified the timing of hypertonic treatment and discovered that hypertonic treatment on day 1–2 only could also effectively promote DE differentiation (Figure S3G). More importantly, we investigated whether manipulating cell size through hypertonic conditions could decrease the requirement for activin A, a widely used but costly growth factor. We reduced the concentration of activin A greatly to 10% (from 100 ng/mL to 10 ng/mL) and found that hypertonic conditions could efficiently induce DE formation at 10 ng/mL, comparable to the normal condition with 100 ng/mL activin A (Figure S3H), thereby reducing costs. Nevertheless, these results indicated that smaller cells were more prone to endoderm differentiation under hypertonic pressure.

To gain a better understanding of the impact of hypertonic pressure on endoderm differentiation, we conducted an RNA-seq experiment using isotonic and hypertonic DE cells. We found that hypertonic pressure downregulated the expression of key pluripotency genes while promoting endodermal gene expression (Figure 2F). Using a cutoff fold-change >2 and *p* < 0.05, we identified 1,675 differentially expressed genes between hypertonic and isotonic DE cells, with 1,212 genes upregulated in hypertonic DE cells. Many of the upregulated genes were associated to developmental processes, such as cell differentiation, tube development and epithelium development (Figure 2G). Notably, the upregulated genes were linked to digestive system development and maturity onset diabetes of the young (MODY), which are critical processes in the later stages of endoderm development. Meanwhile, the downregulated genes in hypertonic DE cells were significantly associated with the negative regulation of developmental process, mesoderm development, and nervous system development (Figure 2G). These results indicate that the reduction in cell size caused by hypertonic pressure specifically promotes the differentiation of human ESCs toward the endodermal fate.

To determine the impact of modulating cell size on endodermal developmental competence, we employed the same protocol to induce isotonic DE and hypertonic DE into endodermal lineages. We first observed that hypertonic DE cells exhibit higher expression levels of markers associated with pancreatic (PDX1), intestinal (CDX2), or hepatic (AFP) lineages (Figures S3I and S3J). Based on our previously developed protocol for differentiating pancreatic beta cells (Jiang et al., 2015; Tan et al., 2019), we further evaluated the beta cell differentiation competence of hypertonic DE cells. The results indicated that hypertonic DE cells were able to differentiate into insulin-positive β-like cells, similar to the isotonic DE cells (Figure S3K). Taken together, these data collectively demonstrate that treatment with hypotonic condition does not impact the developmental competence.

### Actomyosin plays a role in endoderm differentiation boost caused by cell size diminution

Mechanosensitive ion channels, focal adhesion, and the actomyosin cytoskeleton are major factors that respond to changes in cell volume (Syeda et al., 2016; Hoffmann et al., 2009; Venkova et al., 2022). Since ESCs and DE cells have different expression levels of mechanosensitive ion channels, focal adhesion, and actomyosin-related genes (Figures 1E and 1F), we conducted an analysis of these genes in isotonic and hypertonic DE cells by RNA-seq. The results showed that most genes related to mechanosensitive ion channel were upregulated under hypertonic condition, while no consistent changes were observed in genes related to focal adhesion and the actomyosin cytoskeleton (Figures S4A and S4B). To investigate the effect of volume compression on DE differentiation, we applied agonists and antagonists of these targets during the differentiation process. We observed that the activation of Piezo1 was necessary for DE differentiation, while manipulating Piezo1 activity through either activator Yoda1 or inhibitor GsMTx-4 did not affect the enhancement of differentiation caused by hypertonic pressure (Figure S4C). Though inhibiting focal adhesion kinase (FAK) by PF-573228 or Src-family kinase by PP1 both hindered DE differentiation, they did not affect the influence of hypertonic pressure (Figure S4D). Additionally, we observed that actin polymerization was required for DE differentiation and hypertonic pressure could counteract the suppression of DE differentiation caused by actin polymerization inhibitor latrunculin A (Figure S4E). However, the actin polymerization agonist jasplakinolide did not further promote DE differentiation, suggesting that actin polymerization was already functionally saturated for DE differentiation and not the cause of hypertonic DE fate promotion. We also checked the effect of hypertonic treatment upon mitogen-activated protein kinase (MAPK) inhibition. Our data showed that inhibiting MAPKs could reduce the efficiency of DE differentiation as expected (Lau et al., 2023; Loh et al., 2014; Yap et al., 2014), but it did not affect the promoting effect of hypertonic treatment on DE differentiation (Figures S4F and S4G). These data do not support that osmotic stress impacts DE differentiation through MAPK.

**Figure 3:**
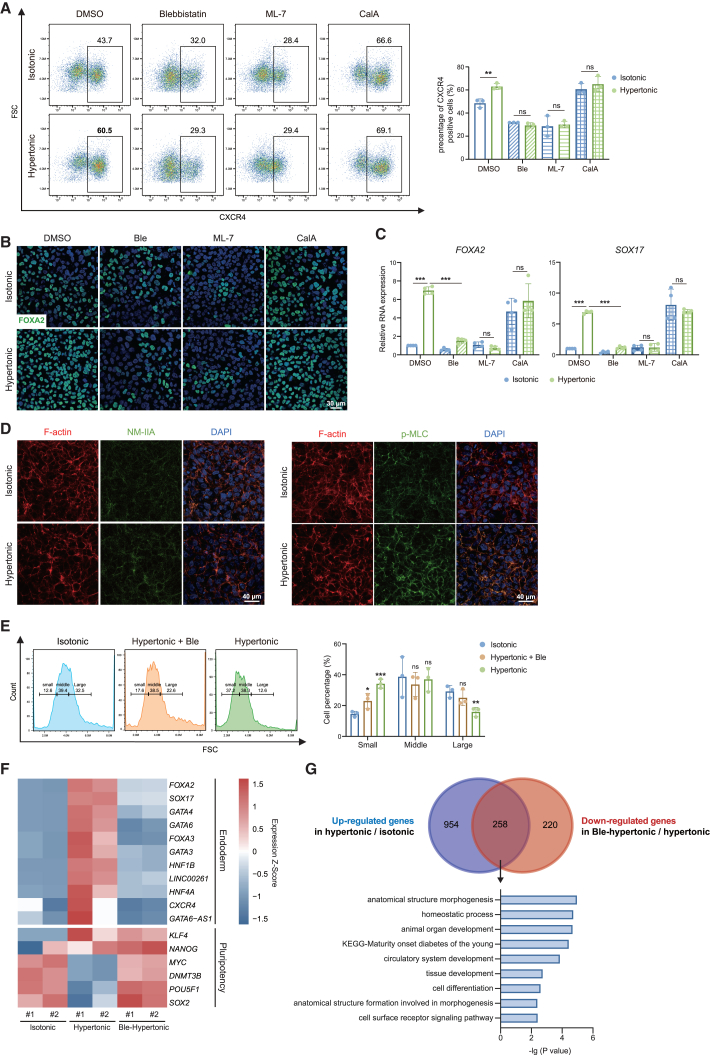
Actomyosin plays a role in endoderm differentiation boost caused by cell size diminution (A) Quantitative flow cytometric analysis of CXCR4 expression in DE cells in isotonic or hypertonic medium with or without myosin-related chemicals (*n* = 3 from 3 independent batches). 10 μM blebbistatin (myosin inhibitor), 2 μM ML-7 hydrochloride (myosin inhibitor), or 0.5 nM calyculin A (myosin agonist) was used. (B) Immunostaining of FOXA2 (green) showing DE cells in isotonic or hypertonic medium with or without myosin-related chemicals (DAPI; blue) (scale bar is 30 μm). (C) Relative mRNA expression of DE marker genes (*n* = 4 from 4 independent batches). (D) Immunostaining of fluorescently labeled phalloidin (red, marking F-actin) and non-muscle myosin IIA (NMMIIA) or phospho-myosin light chain (p-MLC) of isotonic or hypertonic differentiated DE cells (scale bar is 40 μm). (E) Size distribution of ESCs treated with or without blebbistatin was determined by FSC. Gates of small, middle, and large cell size and quantification of gates percentage are indicated (*n* = 3 from 3 independent batches). (F) The heatmap showed the differentially expressed DE and ESC marker genes after differentiation in isotonic or hypertonic medium with or without blebbistatin. (G) Venn diagram showing the overlap of upregulated genes in hypertonic condition (compared with isotonic DE cells) and downregulated genes in Ble-hypertonic condition (compared with hypertonic DE cells). GO analysis of these overlap genes (ns means not statistically significant, ^∗^*p* < 0.05, ^∗∗^*p* < 0.01, ^∗∗∗^*p* < 0.001).

Interestingly, the abolishment of actomyosin contractility using myosin inhibitor blebbistatin and ML-7, or its enhancement via treatment with calyculin A, was found to suppress or promote DE differentiation, respectively (Figure 3A). Meanwhile, all three myosin inhibitors or activators used were able to eliminate the promotion of endoderm differentiation by hypertonic pressure. This is confirmed by analyzing the expression of the endoderm markers (Figures 3B and 3C). This result indicates that myosin activity plays an active role in boosting endoderm differentiation under hypertonic pressure. In addition, we stained cells with fluorescently labeled phalloidin, which binds to F-actin, and with antibodies against non-muscle myosin IIA (NMMIIA) or phospho-myosin light chain (p-MLC), a marker of the activated form of myosin (Rosowski et al., 2015). The organization of actin in hypertonic cells showed no obvious difference from isotonic cells. However, hypertonic treatment increases the co-localization of NMMIIA and actin (Figure 3D), indicating a more mature actomyosin structure. Interestingly, elevated p-MLC content was accompanied by strong co-localization of activated myosin and actin in hypertonic-treated cells (Figure 3D), suggesting enhanced actomyosin activity under hyperosmotic conditions. In addition, the diminution in cell size under hypertonic treatment could be partially relieved by treatment with myosin inhibitors, as evidenced by microscopy and flow cytometry analysis (Figures 3E and S3H). These results suggest that the decrease in cell size under hypertonic treatment is dependent on actomyosin activation (Koushki et al., 2023).

To investigate the crucial role of actomyosin in hypertonic DE differentiation and whether inhibiting actomyosin activity can reverse the transcriptomic changes in hypertonic DE cells, we performed an RNA-seq experiment using isotonic, hypertonic, and Ble-treated hypertonic DE cells. Actomyosin inhibition rescued the decreased expression of the key pluripotent genes and increased the expression of endodermal genes induced by hypertonic pressure (Figure 3F). Using a cutoff fold-change >2 and *p* < 0.05, we identified 478 downregulated genes in Ble-hypertonic DE cells compared to cells treated with hypertonic solution alone. Interestingly, more than half of these downregulated genes (258/478) were found to be upregulated in hypertonic DE cells (1,212 genes) (Figure 3G). These overlapping genes were associated with developmental process, homeostatic process, and MODY (Figure 3G). These observations suggest that the enhancement of endoderm differentiation due to cell size decrease is largely dependent on the activation of actomyosin; moreover, the activation of myosin likely contributes to cell compression in hypertonic conditions.

### YAP signal pathway participates in hypertonic endoderm differentiation

Since cell size reduction promotes DE differentiation from ESCs (Figure 2), our focus shifted to mechanosensitive signal pathways such as WNT/beta-catenin, Rho-associated protein kinase (ROCK), and YAP pathways. The WNT pathway, a crucial regulator of DE differentiation (Jiang et al., 2013b), is enhanced by intracellular crowding caused by cell volumetric compression (Li et al., 2021b). Rho/ROCK has been reported to control cell size (Tilly et al., 1996; Estevez et al., 2001; Sordella et al., 2002) and regulate gene expression (Taglietti et al., 2018; Yoshikawa et al., 2015). Thus, we used agonist and antagonist of these signaling pathways to determine their contribution to the hypertonic DE differentiation boost. We found that persistent activation of the WNT pathway restrained DE differentiation, and intervention in the WNT pathway did not affect the hypertonic differentiation boost (Figure S5A). Though inhibition of ROCK impeded DE differentiation, it failed to minimize the influence of hypertonic pressure (Figure S5B). These results indicated that the promotion of DE differentiation under hypertonic pressure was independent of the ROCK or WNT signaling pathway.

**Figure 4:**
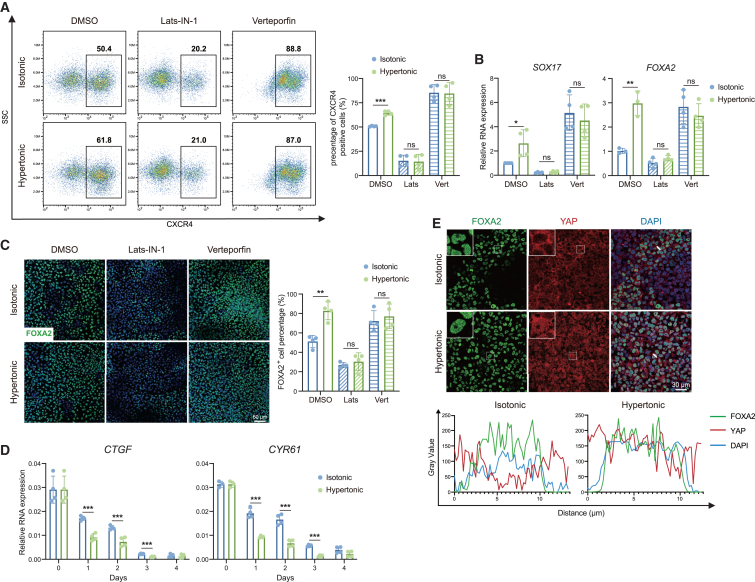
YAP signal pathway participates in the hypertonic endoderm differentiation boosting (A) Quantitative flow cytometric analysis of CXCR4 expression in DE cells in isotonic or hypertonic medium with or without YAP-related chemicals (*n* = 4 from 4 independent batches). 1–10 μM Lats-IN-1 (YAP agonist) or 0.1–1 μM verteporfin (YAP inhibitor). (B) Relative mRNA expression of DE marker genes (*n* = 4 from 4 independent batches). (C) Immunostaining of FOXA2 (green) showing DE cells in isotonic or hypertonic medium with or without YAP-related chemicals (DAPI; blue) (scale bar is 60 μm). Quantifications of the positive staining shown as right. (D) Time-course analysis of YAP target genes expression during isotonic or hypertonic DE differentiation (*n* = 4 from 4 independent batches). (E) Confocal analysis of immunofluorescent images of YAP and FOXA2 in isotonic or hypertonic DE cells. Representative plots of fluorescent signals intensity along the white line for FOXA2 (green), YAP (red), and DAPI (blue) (scale bar is 30 μm) (ns means not statistically significant, ^∗^*p* < 0.05, ^∗∗^*p* < 0.01, ^∗∗∗^*p* < 0.001).

The YAP signaling pathway serves dual roles as a sensor and regulator of cell volume (Perez Gonzalez et al., 2018; Perez-Gonzalez et al., 2019; Pagliari et al., 2021; Sun et al., 2020). Thus, we applied Lats-IN-1 (YAP agonist) or verteporfin (YAP inhibitor), both of which abolished the hypertonic pressure-induced promotion of DE differentiation (Figure 4A). Importantly, YAP pathway suppression significantly enhanced DE lineage differentiation (Figures 4B and 4C). We hypothesized that hypertonic cell compression reduces YAP pathway activity. Through the analysis of gene expression related to the YAP pathway, we found that hypertonic pressure increased the expression of YAP and TAZ, while YAP target genes, such as *TEAD*, *CYR61*, and *BIRC5*, exhibited decreased expression under hypertonic pressure (Figure S5C). We conducted a further analysis of the temporal expression of YAP target gene *CYR61* and *CTGF* (Price et al., 2021). The result showed that the activity of YAP pathway decreased during DE differentiation and hypertonic pressure further accelerated this downregulation (Figure 4D). Moreover, inhibition of myosin partially reversed the suppressive effect of hypertonic pressure on YAP activity (Figure S5C). Given that YAP activity correlates with its intracellular localization (Nishioka et al., 2009), we observed nuclear YAP localization in ESCs and cytoplasmic localization in DE cells under isotonic conditions (Figures 4E and S5D). Unexpectedly, hypertonic treatment induced partial nuclear localization of YAP protein in DE cells but had minor effect on YAP localization in ESCs. This nuclear translocation of YAP caused by hypertonic pressure was not insensitive to actomyosin inhibition (Figure S5E), indicating a high sensitivity to cell size reduction independent of the cytoskeleton. Therefore, we hypothesized that a YAP repressor, responsive to cell compression through actomyosin, may suppress YAP activity in the nucleus.

### Cell size diminution disrupts YAP activation via promoting AMOT nuclear translocation

**Figure 5:**
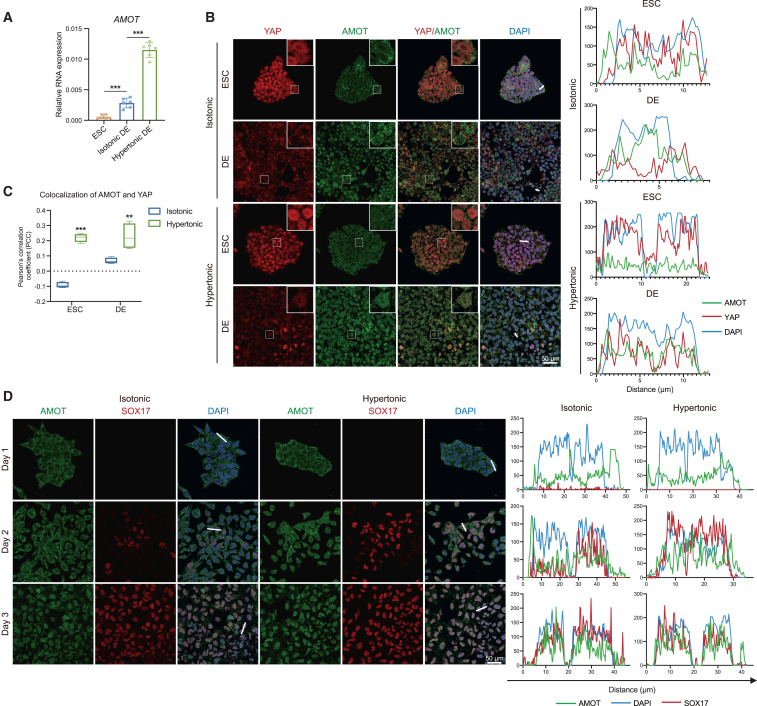
Cell size compression promotes the nuclear translocation of AMOT to co-localize with YAP (A) Relative mRNA expression of *AMOT* in ESCs and DE cells under isotonic or hypertonic condition (*n* = 6 from 6 independent batches). (B) Confocal analysis of immunofluorescent images of YAP and AMOT in isotonic or hypertonic ESCs and DE cells. Magnified views of the regions in the white boxes are provided. Representative plots of fluorescent signals intensity along the white line for YAP (red), AMOT (green), and DAPI (blue) (scale bar is 50 μm). (C) Pearson’s coefficient of YAP and AMOT proteins’ co-localization. (D) Confocal analysis of immunofluorescent images of time-course AMOT nuclear translocation in isotonic or hypertonic DE-differentiated cells. Representative plots of fluorescent signals intensity along the white line for SOX17 (red), AMOT (green), and DAPI (blue) (scale bar is 50 μm) (ns means not statistically significant, ^∗∗^*p* < 0.01, ^∗∗∗^*p* < 0.001).

We then investigated the YAP repressor that is associated with hypertonic pressure during DE differentiation. From the literature, we identified two well-known YAP repressors, ARID1A and AMOT. The nuclear actin-binding factor ARID1A can sequester YAP away from TEADs and inhibit its activity (Chang et al., 2018). However, the expression of *ARID1A* was downregulated during DE differentiation from ESCs and upregulated under hypertonic pressure (Figure S6A). This suggests that ARID1A is not the suppressor of YAP activity under hypertonic pressure. AMOT inhibits YAP activity through directly interacting with YAP or regulating Lats1/2 activation in cytoplasm. Additionally, AMOT can directly bind with YAP in nucleus (Wang et al., 2015; Nakajima et al., 2017). Interestingly, AMOT was reported to be involved in neural differentiation and mesoderm specification (Zaltsman et al., 2019; Pagliari et al., 2021). We observed an increase in the mRNA level of *AMOT* during DE differentiation. Furthermore, AMOT exhibited a higher level after hypertonic pressure (Figure 5A). By confocal examination, we found that AMOT was localized in the cytoplasm of ESCs but in the nucleus of SOX17-positive DE cells, regardless of isotonic or hypertonic conditions (Figure S6B). Moreover, hypertonic stimulation increased the co-localization of AMOT and YAP in both ESCs and DE cells (Figure 5B). In DE cells, YAP translocated from the cytoplasm to the nucleus and achieved higher Pearson’s correlation with AMOT after hypertonic stimulation (Figure 5C). These observations indicate that the compression of cell size caused by hypertonic pressure promotes the co-localization of AMOT and YAP in nucleus, which is accompanied by decreased YAP activity.

We hypothesized that hypertonic pressure promotes the nuclear localization of AMOT, which in turn accelerates the inactivation of YAP signal pathway. To verify our hypothesis, we examined the temporal localization of AMOT during hypertonic DE differentiation. We observed that hypertonic pressure accelerated the nuclear localization of AMOT and the appearance of SOX17-positive DE cells (Figure 5D). This phenomenon was also observed in another human ESC line, H9 (Figure S6C). On day 2 after DE differentiation, the cytoplasmic localization of AMOT disappeared in the hypertonic treatment group, and nuclear localization began to occur, along with the appearance of SOX17-positive DE cells. This observation is highly consistent with the significant decrease in cell size on day 2 and the effective promotion of DE differentiation by hypertonic treatment only on day 1–2 (Figures 2E and S3G). To investigate the role of actomyosin in AMOT nuclear localization, we treated hypertonic DE differentiation with the actomyosin inhibitor ML-7. The result demonstrated that ML-7 effectively reduces the nuclear localization of AMOT, without significantly affecting the nuclear localization of YAP (Figure S6D). In addition, by assessing the expression of YAP target genes, we found that decreased co-localization of AMOT and YAP, caused by myosin inhibition, could activate YAP pathway under hypertonic condition (Figure S5C). Taken together, these observations demonstrate that the decrease in cell size promotes AMOT nuclear localization through actomyosin and increased the co-localization of AMOT and YAP, leading to reduced YAP signal activity.

**Figure 6:**
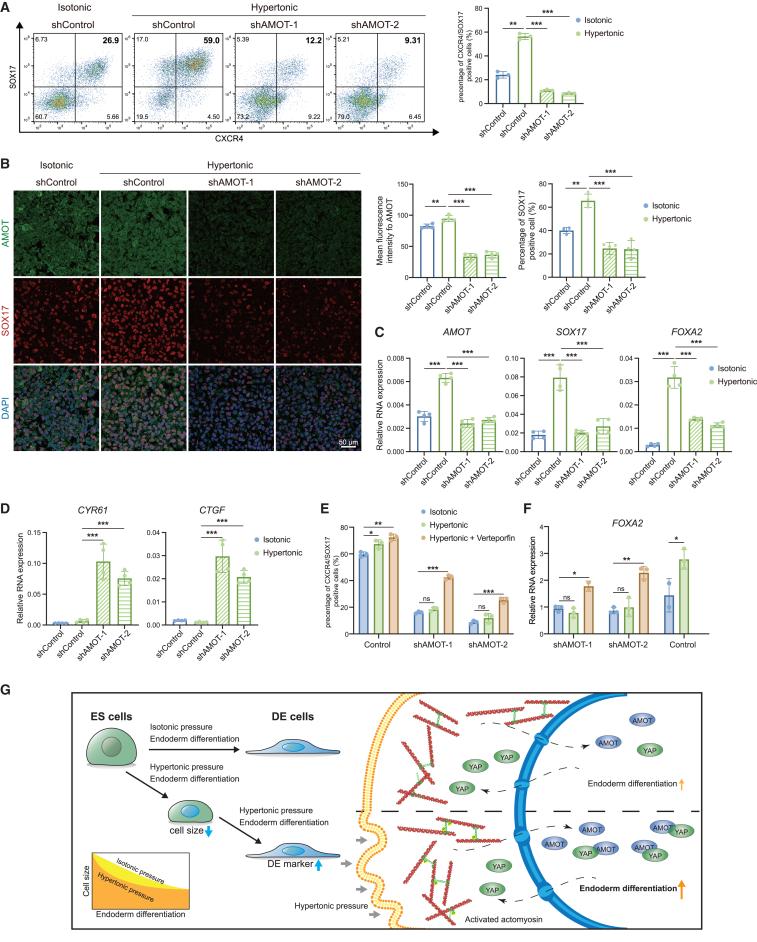
AMOT contributes to the promotion effect of cell size diminution on DE differentiation by inhibiting YAP pathway (A) Quantitative flow cytometric analysis of CXCR4 and SOX17 expression in shControl and shAMOT DE cells under hypertonic condition (*n* = 3 from 3 independent batches). (B) Representative confocal images for AMOT (green) and SOX17 (red) as detected in shControl and shAMOT DE cells under hypertonic condition (scale bar is 50 μm). Quantifications of the intensity of the AMOT fluorescent signals (a.u.) and percentage of SOX17-positive cell (%), with nuclei counterstained with DAPI (blue), shown as right. (C and D) Relative mRNA expression of *AMOT*, DE marker (C) and YAP target genes (D) in shControl and shAMOT DE cells under hypertonic condition. (E and F) Quantitative flow cytometric analysis of CXCR4 and SOX17 expression (E) and RNA expression of DE marker gene *FOXA2* (F) in shControl and shAMOT DE cells under hypertonic condition, with or without YAP inhibitor verteporfin (*n* = 3 from 3 independent batches). (G) Model of hypertonic condition promoting endoderm differentiation via actomyosin-dependent cell size diminution, and AMOT nuclear translocation, which suppresses YAP activity (ns means not statistically significant, ^∗∗^*p* < 0.01, ^∗∗∗^*p* < 0.001).

To investigate the direct effect of AMOT on hypertonic DE differentiation, we used short hairpin RNA to target AMOT and established two human ESC lines with stable AMOT knockdown (Figure S7A). AMOT knockdown did not affect the colony morphology or expression of pluripotent genes and YAP-related genes in ESCs (Figures S7B–S7E), indicating that AMOT was not active in the undifferentiated status. Next, we subjected the AMOT knockdown ESCs to hypertonic pressure during DE differentiation. We found a significant decrease in the proportion of CXCR4-/SOX17-double-positive DE cells in hypertonic AMOT knockdown cells compared to control cells (Figure 6A). Furthermore, AMOT knockdown did not alter the localization of AMOT but significantly decreased the protein level of AMOT and SOX17 in DE cells (Figure 6B). These results indicated that the absence of AMOT could abolish the promotion of hypertonic DE differentiation, which is further supported by reverse-transcription PCR analysis of DE marker genes (Figure 6C). In addition, AMOT knockdown cells appeared to have increased YAP activity under hypertonic pressure (Figures 6D and S7F). Consistently, YAP inhibitors effectively rescued the decreased efficiency of DE differentiation caused by AMOT knockdown, suggesting that AMOT functions upstream of YAP (Figures 6E and 6F). In contrast, hypertonic treatment did not enhance the decreased DE induction caused by AMOT knockdown (Figures 6E and 6F), as AMOT functioned downstream of hypertonic treatment. Moreover, AMOT knockdown cells also exhibited smaller cell size (Figure S7G). In summary, these results demonstrate that the promotion of hypertonic DE differentiation relies on AMOT, which interferes with YAP activation.

### Cell size and YAP signaling pathway are actively implicated in human early embryo development

**Figure 7:**
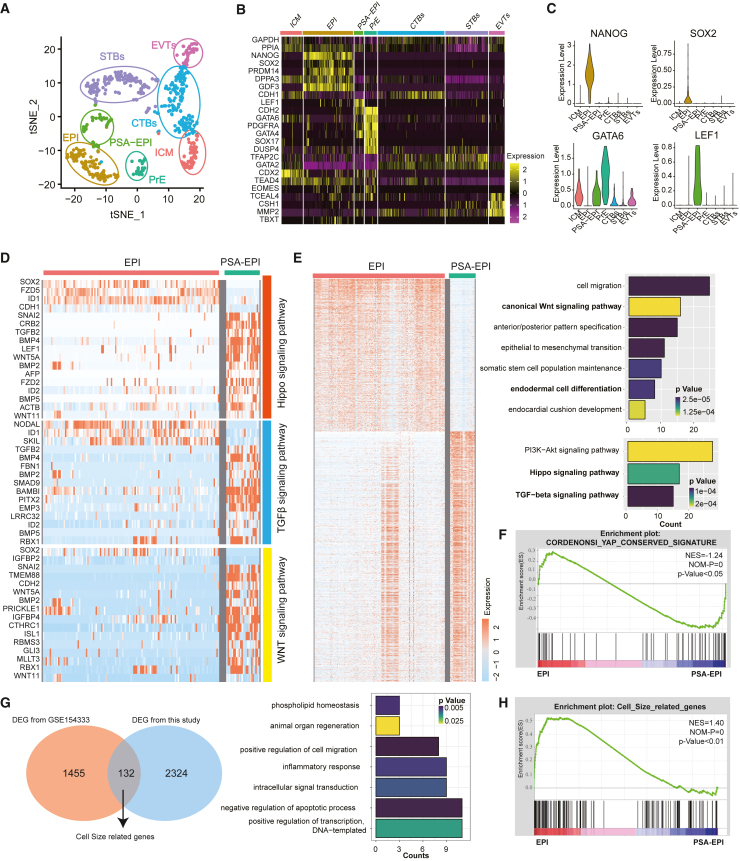
Cell size and YAP signal pathways likely involve in human early development (A) t-SNE analyses revealed 8 clusters, including EPI (epiblast) and PSA-EPI (primitive streak anlage derived from epiblast). (B) Different clusters expressed lineage-specific markers. (C) Violin plot showing expression of marker genes for EPI and PSA-EPI. (D) Heatmap showing expression levels of genes related to DE-associated signaling pathways (TGF-β and WNT as well as Hippo/YAP) between EPI and PSA-EPI. (E) Heatmap and representative GO terms showing expression levels of differentially expressed genes (DEGs) between EPI and PSA-EPI. (F) GSEA showing YAP signaling was enriched in DEGs between EPI and PSA-EPI. (G) Venn diagram defining the cell-size-related genes based on GSE154333 and this study and representative enriched GO terms for this gene set. (H) GSEA showing cell-size-related gene set was enriched in DEGs between EPI and PSA-EPI.

Our present study reveals that cell size diminution influences DE induction through YAP signaling; however, whether it happens *in vivo* is unknown yet. To investigate this issue, we surveyed the single-cell RNA-seq dataset from 3D-cultured human pre-gastrulation embryos (GSE136447) (Xiang et al., 2020), which contains different cell types around gastrulation stage: embryonic disc, amnion, basement membrane, primary and primate unique secondary yolk sac, anterior-posterior polarity formation, and primitive streak anlage (PSA). We downloaded and re-analyzed the data. We first repeated the annotation of different cell types (Figure 7A), particularly the epiblast (EPI) and the epiblast-derived PSA (PSA-EPI). The EPI sample highly expresses *NANOG* and *SOX2* while PSA-EPI sample highly expresses *GATA6* and *LEF1* as expected (Figures 7B and 7C). By analyzing the differentially expressed genes and enriched signaling pathways, we found the HIPPO/YAP signaling as well as WNT and transforming growth factor β (TGF-β) pathways (Figures 7D and 7E), indicating HIPPO/YAP actively involved in early embryonic germ layers’ specification. Gene set enrichment analysis (GSEA) also supported this notion (Figure 7F). In addition, since no any terms related to “cell size” was found from the Gene Ontology (GO) or GSEA database, we collected another dataset about hematopoietic stem cells with different sizes (Lengefeld et al., 2021) and generated the differentially expressed genes, followed by overlapping those genes with the differentially expressed genes in our study (with hypertonic treatment, Figure 2G). We labeled the overlapped 132 genes as “cell-size-related genes,” which enriches terms of intracellular signal transduction and phospholipid homeostasis (Figure 7G). We then performed the GSEA and found the cell-size-related genes were significantly enriched during early development (Figure 7H). These data support that cell size and HIPPO/YAP signaling pathways actively involved in human early embryo development. Due to the ethnical and technical limitation, the emergingly developing *ex vivo* cultured human gastruloids (Liu et al., 2023; Rivron et al., 2023) would provide a choice to comprehensively explore the function of cell size control in human early development. In fact, a recent report based on the 2D-micropatterned human ESC-derived gastruloids revealed that the knockout of YAP1 could result in hyperactive NODAL signaling which retained SMAD2/3 in the nuclei, thus impeding ectoderm differentiation and enlarging mesoderm and endoderm layers (Stronati et al., 2022). Mechanistically, YAP-TEAD could block SMAD2/3 induction of mesendodermal genes by regulating binding of the NELF negative elongation factor to impair SMAD recruitment (Estarás et al., 2015); YAP knockout enabled activin to induce WNT3 expression and stabilize β-catenin, facilitating mesendodermal differentiation (Estarás et al., 2017). These reports together with our present observation provide a comprehensive understanding on early embryonic development from the angles of intracellular modulation of YAP/SMAD/WNT and extracellular stimuli including hypertonic cell size manipulation. Furthermore, there remains a lack of direct evidence regarding the potential promotion of the activin/Nodal signaling pathway due to the inhibition of YAP signaling induced by hypertonic cell volume reduction, necessitating further investigation in future studies.

## Discussion

Cellular scale architecture is a key characteristic of cell state, supporting the cell functionality required for proper embryonic development. Changes in cell volume could impact cell senescence, spreading, and the cell cycle. However, the extent to which cell size affects ESC behavior is not well understood. We exploit DE differentiation as a model to investigate the role of cell size in ESC fate decisions. Our study reveals that DE differentiation involves reduced cell size and increased mechanical state (Figure 1). By inducing cell size reduction through hypertonic treatment or a TRPV4 agonist, we observed significant compression of differentiated cells and enhanced endoderm fate, accompanied by upregulated expression of developmental and endoderm lineage genes (Figure 2). Furthermore, we found that myosin activity participates in hypertonic cell size diminution and promotion of endoderm fate, and a myosin inhibitor partially reverses the upregulation of genes in hypertonic DE cells (Figure 3). We discovered that DE lineage differentiation is accompanied by the suppression of YAP activity with hypertonic cell compression speeding up this process. Meanwhile, we observed partial YAP protein nuclear localization in hypertonic DE cells compared to isotonic ones (Figure 4). Then, we investigated the YAP suppressor AMOT and discovered that hypertonic pressure accelerates its nuclear localization, enhancing YAP-AMOT co-localization. The nuclear localization of AMOT depends on actomyosin and contributes to DE differentiation by suppressing YAP activity (Figure 5). These findings were corroborated by AMOT knockdown experiments, which showed that inhibiting AMOT restricts the heightened DE differentiation induced by YAP signaling (Figure 6). Thus, we propose a model in which hypertonic conditions induce cell volume reduction, promoting DE differentiation (Figure 6G). Hypertonic pressure during DE differentiation accelerates cell volume reduction primarily through actomyosin remodeling, prompting AMOT translocation to the nucleus. This sequence suppresses YAP activity via AMOT-YAP binding, ultimately facilitating DE differentiation.

To understand how hypertonic pressure promotes endoderm differentiation, we manipulated cell-size-sensitive elements (Figures 3 and S4). Piezo1, a mechanosensitive ion channel, regulates cell contractility and volume (Syeda et al., 2016; Clapham, 2007; Hua et al., 2010). FAK and Src activate the volume-regulated anion channel, crucial for cellular responses to osmotic stress (Browe and Baumgarten, 2006; Hoffmann et al., 2009). As part of the cytoskeleton, the actomyosin cytoskeleton generates contractile tension through ATP hydrolysis, involving non-muscle myosin II and actin filaments (F-actin), influencing cell volume and morphology (Venkova et al., 2022; Hartman and Spudich, 2012). Remodeling of the actomyosin cytoskeleton is critical for mesoderm differentiation from pluripotent stem cells, characterized by a transition from cortical actin to stress fibers and associated cell compression (Pagliari et al., 2021). However, the functional effect of the actomyosin cytoskeleton under osmotic pressure remains unexplored. Potential explanations include the reduced spatial distance between F-actin and myosin due to altered intracellular crowding (Li et al., 2021b), or fluctuations in actomyosin cytoskeleton polymerization and depolymerization rates influenced by cytoplasmic viscosity (Molines et al., 2022). Further studies are needed to elucidate these specific mechanisms.

The nuclear localization of AMOT is regulated by its phosphorylation status; increased phosphorylation directs AMOT to the cytoplasm, while hypo-phosphorylation favors nuclear localization (Kang et al., 2020). However, understanding how hypertonic pressure or the actomyosin cytoskeleton influences AMOT’s nuclear entry remains challenging. Thus, we propose another mechanism: cells establish a physical connection between nuclei and the actomyosin cytoskeleton, wherein reduced cell size flattens nuclei, stretches nuclear pores, and lowers their mechanical resistance to molecular transport (Elosegui-Artola et al., 2017). Therefore, we speculate that earlier nuclear entry of AMOT in hypertonic DE cells depends on nuclear pore stretching and sensitivity to actomyosin cytoskeleton. YAP defines the first cell fate choice in the mouse embryo and aberrant YAP expression leads to embryonic lethality (Varelas, 2014). Moreover, human pluripotent stem cells display sustained basal YAP-driven transcriptional activity, which inhibits cytoskeleton dynamics and maintains pluripotency. When germ layers are specified, the YAP-TEAD complex is promptly inactivated, and cytoskeleton remodeling can occur (Pagliari et al., 2021). AMOT inhibits YAP activity through direct binding, acting as a scaffold protein to promote YAP phosphorylation and degradation through LATS1/2 kinases (Wang et al., 2022), or leading to YAP cytoplasmic retention (Zaltsman et al., 2019). Given that AMOT can bind to YAP in the nucleus (Li and Fan, 2017), we speculate that AMOT binds to YAP and competitively inhibits YAP interaction with TEAD or promotes the binding of YAP with nuclear actin. However, the specific mechanism still needs to be further explored in subsequent studies.

In summary, we report that cell size decreases during human ESC differentiation into endoderm, with hypertonic pressure enhancing DE differentiation. The actomyosin cytoskeleton plays a critical role in reducing cell size and promoting endoderm fate under hypertonic pressure. Cell size reduction suppresses YAP activity by facilitating AMOT nuclear translocation, thereby promoting endoderm differentiation. Therefore, our study provides insights into the functional relationship between cell size, physical state, and intracellular signaling during endoderm differentiation. Therefore, our study provides valuable insights into the role of cell size, by establishing a connection between the physical state of cells and intracellular signaling.

### Limitations of the study

Cell size is a complex feature that may affect numerous other aspects. During cell culture, cell size fluctuates due to factors such as cell cycle, growth, or mechanical environment. Apart from the signaling molecules mentioned in the manuscript, many other molecular changes may occur, warranting exploration in future studies. In addition, the protocol for endoderm differentiation is not optimized in different culture systems and therefore may not be generalizable. Furthermore, there is a lack of direct *in vivo* experiments to prove the conclusions of this study, necessitating further investigation in future, such as the emerging human embryoid/gastruloid system.

## Experimental procedures

### Resource availability

#### Lead contact

Further information and requests for resources and reagents should be directed to and will be fulfilled by the lead contact, Donghui Zhang (dongh.zhang@hubu.edu.cn) or Wei Jiang (jiangw.mri@whu.edu.cn).

#### Materials availability

Plasmids and cell lines generated in this study should be directed to and will be fulfilled by the lead contact.

This study did not generate new unique reagents.

#### Data and code availability

The RNA-seq data in this study have been uploaded to the Gene Expression Omnibus (GEO) database under accession number GSE232608. The processed RNA-seq data are available in the GEO database.

All original microscopy data reported in this paper will be shared by the lead contact upon reasonable request.

### Methods details

#### Cell culture and differentiation

Two different human ESC lines, H9 and HUES8, were used in this study. They were cultured in mTeSR1 medium (STEMCELL Technologies, #AB217641) on Matrigel-coated plates. Our work on human ESCs and iPSCs is approved by the Biomedical Ethics Committee of Wuhan University (WHU-LFMD-IRB2024026). Human embryonic kidney 293T (HEK293T) cells were cultured with DMEM containing 10% fetal bovine serum (Gibco, #10270–106) and 1% penicillin-streptomycin (Gibco, #15140163). The endoderm differentiation protocol of human ESCs was based on the previous reports (Lu et al., 2023; Yang et al., 2020; Jiang et al., 2013a) with minor modifications. IMDM (Gibco, #C12440500BT) and F12 (Gibco, #C11765500BT) mixed at a ratio of 1:1 (IMDM/F12) were used as basal medium, supplemented with 0.2% BSA (YEASEN, #36106ES76), 1% B27 (without Vitamin A, Shanghai BasalMedia Technologies, S441J7), and 1% penicillin-streptomycin and activin A (100 ng/mL, PeproTech, #120–14P) for 3 or 4 days for endoderm differentiation. DMEM could be also used as basal medium instead of IMDM/F12 and the rest ingredients were the same as aforementioned.

Hypertonic stimulation was performed by adding different amounts of polyethylene glycol 300 (PEG300) (Selleck, #S6704) or sucrose (Hushi, #10021418) to isotonic culture medium as reported (Casula et al., 2017; Li et al., 2021b). Isotonic culture medium was defined as control medium. Based on previous studies, PEG300 is a polymer preventing non-specific protein binding and, after adding PEG300 for 2 min, cell size and mechanics achieved equilibration (Guo et al., 2017; Akabayov et al., 2013). When indicated, 1 μM Yoda1 (Topscience, #T7506), 5 μM GsMTx-4 (MCE, #HY-P1410A), 1 μM PP1 (Topscience, #T6196), 1 μM PF-573228 (Topscience, #T2001), 10–100 nM jasplakinolide (Abcam, #ab141409), 0.1–1 μM latrunculin A (Cayman Chemical, #10010630), 10 μM blebbistatin (Topscience, #T6038), 2 μM ML-7 hydrochloride (MCE, #HY-15417), 0.5 nM calyculin A (MCE, #HY-18983), 2.5 μM CHIR-98014 (Selleck, #S2924), 2 μM IWR-1 (Selleck, #S7086), 10 μM Y27632 2HCl (Selleck, #S1049), 1–10 μM Lats-IN-1 (MCE, #HY-138489), 0.1–1 μM verteporfin (Topscience, #T3112), 10 μM GSK205 (MCE, # HY-120691A), 50 nM GSK101 (MCE, # HY-19608), 10 μM (E)-osmundacetone (MAPK inhibitor) (MCE, # HY-N1966), or corresponding amount of vehicle (dimethyl sulfoxide [DMSO, Sigma, #D2438]) was introduced into the endoderm differentiation medium.

#### Cell size measurement

For cell cross-section area and diameter statistics, after experimental treatment, cultured cells are digested into single cells and suspended in their specific culture medium. Suspended cells were visualized and photographed using microscopy (Olympus), and cross-sectioned area and diameter of cells were quantified from images using ImageJ/Fiji. Suspended cell volume was automatically measured by Cellometer Mini Automated Cell Counter (Nexcelom). For 3D confocal assay, single cells were seeded at low density in confocal culture dish. The fluorescent images were obtained by segmenting time-lapse 3D confocal stacks of cells, and the 3D cell volume was computed and reconstructed.

## Supplemental information

Document S1. Figures S1–S7 and Table S1

Document S2. Article plus supplemental information
