# Supplementary material for: Cell size regulates human endoderm specification through actomyosin-dependent AMOT-YAP signaling
Source: Stem Cell Reports. 2024 Aug 1;19(8):1137–55. doi: 10.1016/j.stemcr.2024.07.001 (PMC11368700; doi:10.1016/j.stemcr.2024.07.001)
Supplement: Document S1. Figures S1–S7 and Table S1 [file mmc1.pdf]

Stem Cell Reports, Volume *19*

Supplemental Information

Cell size regulates human endoderm specification through actomyo-sin-dependent AMOT-YAP signaling

Lai Jiang, Chenchao Yan, Ying Yi, Lihang Zhu, Zheng Liu, Donghui Zhang, and Wei Jiang

Supplemental Information

Cell size regulates human endoderm specification through actomyosin-dependent AMOT-YAP signaling

Lai Jiang, Chenchao Yan, Lihang Zhu, Zheng Liu, Donghui Zhang, Wei Jiang

Figure S1. Differentiation induces changes of cell volume and mechanical state.

Figure S2. Hypertonic pressure induces changes of cell volume and enhances human endoderm differentiation.

Figure S3. Cell size diminution promotes endodermal lineage differentiation.

Figure S4. Cytoskeletal plays a role in endoderm differentiation boost caused by cell size decrease.

Figure S5. Identification of cell size related signal pathway that contributes to hypertonic endoderm differentiation.

Figure S6. Cell size compression promotes AMOT nuclear translocation.

Figure S7. AMOT is not essential for pluripotency but influential for YAP activation.

Tables S1. Primer list.

Figure S1

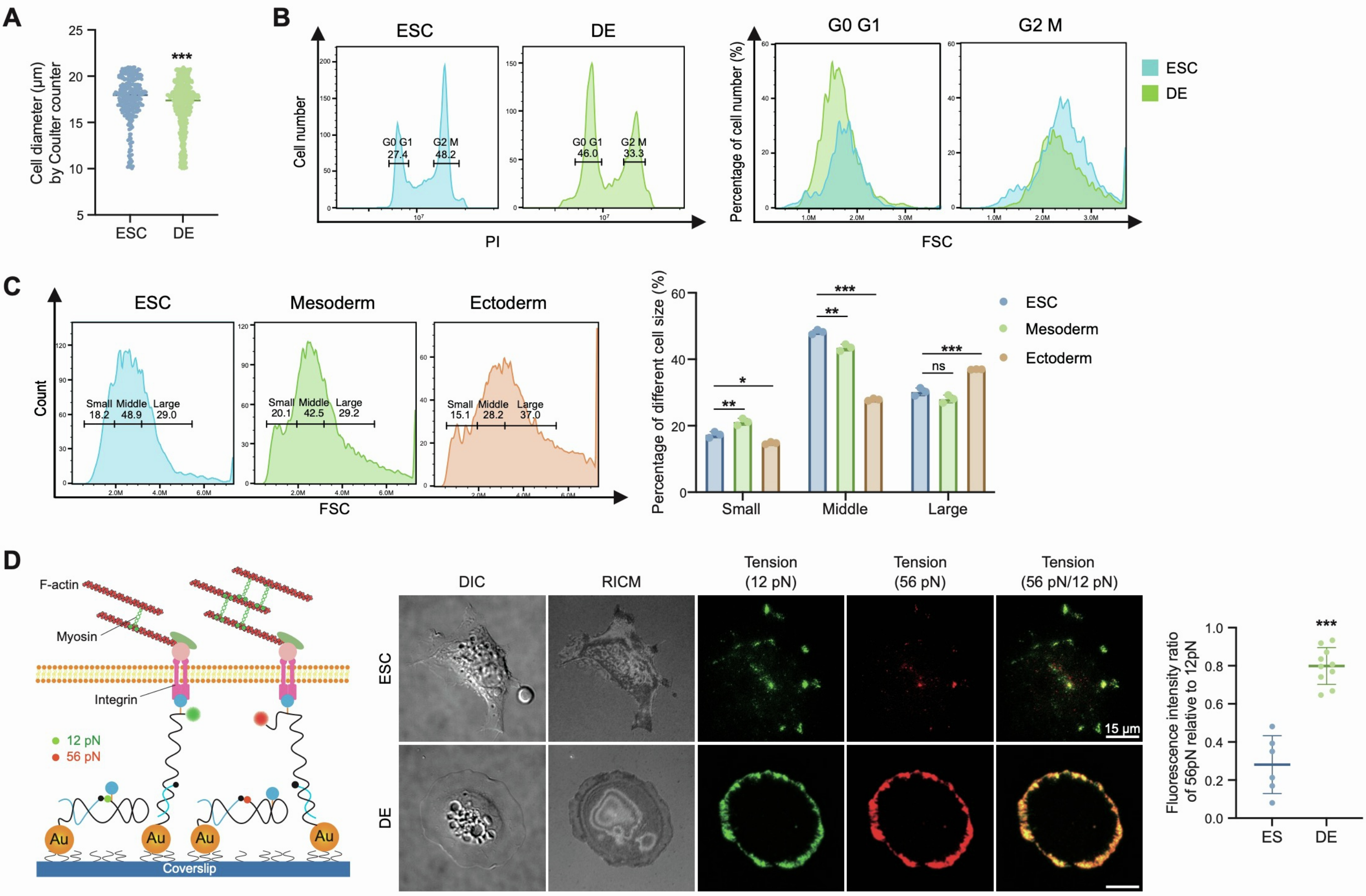

A
Cell diameter (μm) by Coulter counter
ESC
DE
***
B
ESC
DE
Cell number
G0 G1 27.4
G2 M 48.2
G0 G1 46.0
G2 M 33.3
PI
G0 G1
G2 M
Percentage of cell number (%)
FSC
ESC
DE
C
ESC
Mesoderm
Ectoderm
Count
Small 18.2
Middle 48.9
Large 29.0
Small 20.1
Middle 42.5
Large 29.2
Small 15.1
Middle 28.2
Large 37.0
FSC
Percentage of different cell size (%)
Small
Middle
Large
ns
D
F-actin
Myosin
Integrin
12 pN
56 pN
Au
Coverslip
DIC
RICM
Tension (12 pN)
Tension (56 pN)
Tension (56 pN/12 pN)
15 μm
Fluorescence intensity ratio of 56pN relative to 12pN
ES

Figure S1. Differentiation induces changes of cell volume and mechanical state.

(A) Quantification of cell diameter by Coulter counter (n > 330 cells from 3 independent batches per group analyzed).

(B) Flow cytometric analysis of cell cycle (left) and the corresponding size distribution (right) of ESCs and DE cells as determined by FSC.

(C) Flow cytometric analysis of cell size of ESCs, differentiated mesodermal and ectodermal cells. Size distribution of cells as determined by forward scatter (FSC).

(D) Schematic illustrating the interaction between cells and the probe-labeled surface (left). Representative microscopy images of differential interference contrast microscopy (DIC), reflection interference contrast microscopy (RICM, which can reflect the cell adherent area), and total internal reflection fluorescence (TIRF) microscopy of ESCs and DE cells that were seeded on 12pN and 56pN DNA probe surface[21] (middle, scale bar is 15 μm). Statistic total fluorescent intensities and 56pN/12pN fluorescent intensities ratio (right, n > 6 cells per group analyzed).

Figure S2

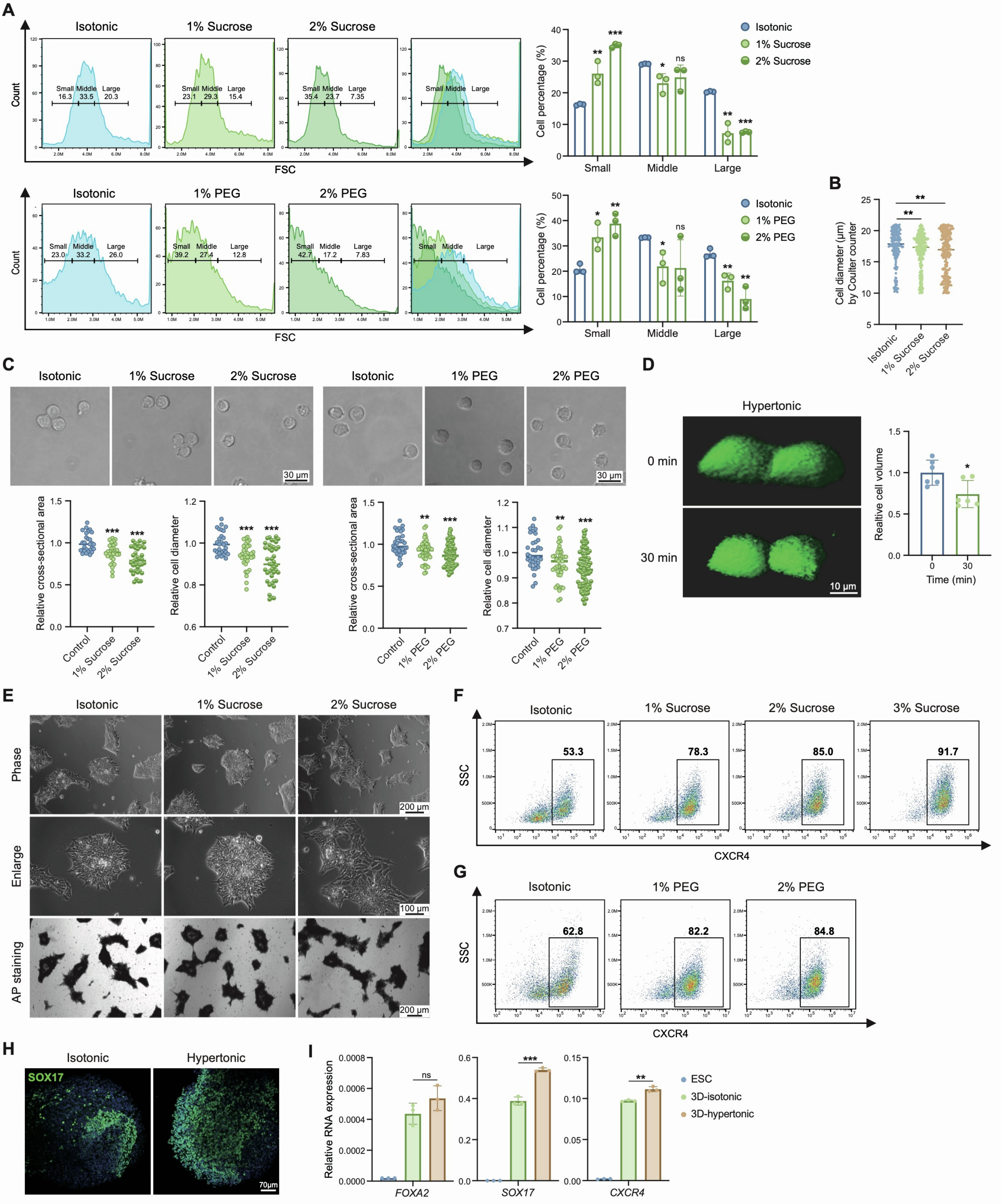

A
Isotonic
1% Sucrose
2% Sucrose
Count
Small Middle Large
16.3 33.5 20.3
23.1 29.3 15.4
35.4 23.7 7.35
FSC
Cell percentage (%)
Small
Middle
Large
Isotonic
1% Sucrose
2% Sucrose
1% PEG
2% PEG
23.0 33.2 26.0
39.2 27.4 12.8
42.7 17.2 7.83
B
Cell diameter (μm) by Coulter counter
C
Control
30 μm
Relative cross-sectional area
Relative cell diameter
D
Hypertonic
0 min
30 min
10 μm
Realtive cell volume
Time (min)
E
Phase
Enlarge
AP staining
200 μm
100 μm
F
3% Sucrose
SSC
CXCR4
53.3
78.3
85.0
91.7
G
62.8
82.2
84.8
H
SOX17
70μm
I
Relative RNA expression
ESC
3D-isotonic
3D-hypertonic
FOXA2
SOX17
CXCR4

Figure S2. Hypertonic pressure induces changes of cell volume and enhances human endoderm differentiation.

(A) Flow cytometric analysis showing ESCs volume decreases with the increasing concentration of sucrose or PEG300, after hypertonic pressure treatment for 48 hours. Size distribution of ESCs as determined by FSC. Gates of small, middle and large cell size and quantification of gates percentage are indicated (n = 3 from 3 independent batches).

(B) Quantification of cell diameter after hypertonic pressure treatment for 48 hours by Coulter counter (n > 190 cells from 3 independent batches per group analyzed).

(C) Representative images of ESCs and quantification of their cross-sectional area and diameter (n > 28 cells from 3 independent batches per group analyzed, scale bar is 30 μm).

(D) 3D images of ESCs after hypertonic pressure and quantification of cell volume (n = 6). Scale bar is 10 μm.

(E) Phase-contrast images of ESCs colonies and bright-field images showing the AP staining of ESCs which has been cultured in isotonic or hypertonic culture medium for 48 hours.

(F-G) Dosage-dependent promoting effect of sucrose (F) or PEG (G) for DE differentiation, measured by flow cytomeric analysis of CXCX4 expression.

(H-I) Immunostaining of SOX17 and RNA expression of DE marker genes under isotonic or hypertonic DE differentiation condition in suspension (3D).

(ns means not statistically significant, *P < 0.05, **P < 0.01, ***P < 0.001).

Figure S3

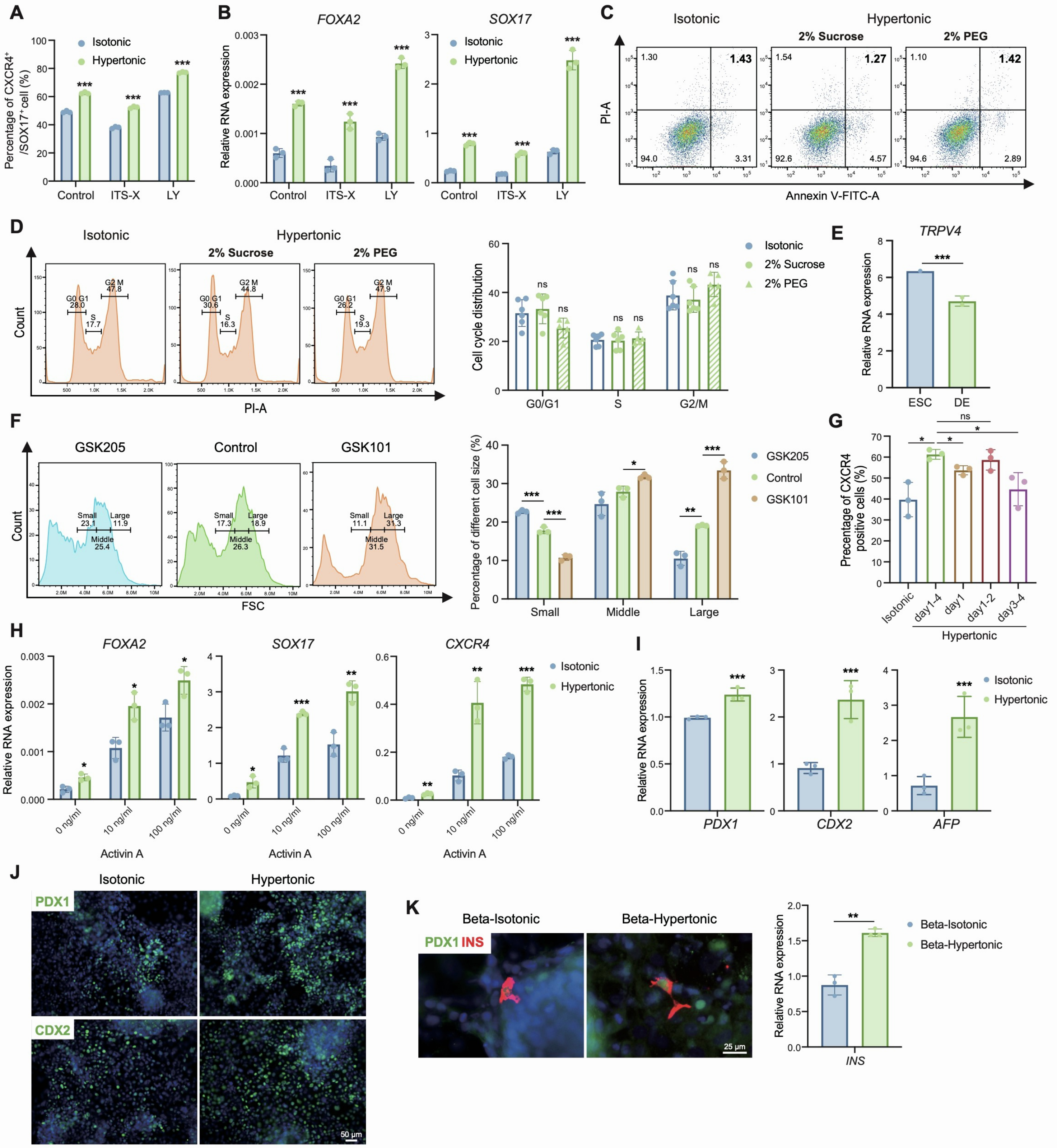

A
Isotonic
Hypertonic
Percentage of CXCR4+ /SOX17+cell (%)
Control
ITS-X
LY
B
FOXA2
SOX17
Relative RNA expression
C
Isotonic
Hypertonic
2% Sucrose
2% PEG
PI-A
Annexin V-FITC-A
1.30
1.43
94.0
3.31
1.54
1.27
92.6
4.57
1.10
1.42
94.6
2.89
D
Count
PI-A
G0 G1 28.0
S 17.7
G2 M 47.8
G0 G1 30.6
S 16.3
G2 M 44.8
G0 G1 26.2
S 19.3
G2 M 47.9
Cell cycle distribution
G0/G1
S
G2/M
E
TRPV4
ESC
DE
F
GSK205
Control
GSK101
Count
FSC
Small 23.1
Large 11.9
Middle 25.4
Small 17.3
Large 18.9
Middle 26.3
Small 11.1
Large 31.3
Middle 31.5
Percentage of different cell size (%)
Small
Middle
Large
G
Precentage of CXCR4 positive cells (%)
Isotonic
day1-4
day1
day1-2
day3-4
Hypertonic
H
FOXA2
SOX17
CXCR4
0 ng/ml
10 ng/ml
100 ng/ml
Activin A
I
PDX1
CDX2
AFP
J
PDX1
CDX2
50 μm
K
Beta-Isotonic
Beta-Hypertonic
PDX1 INS
25 μm
INS

Figure S3. Cell size diminution promotes endodermal lineage differentiation.

(A-B) DE differentiation efficiency determined by flow cytometric analysis of CXCR4 and SOX17 expression (A) and RNA expression of DE marker genes (B) in DE cells under isotonic or hypertonic pressure, treated with PI3K inhibitor LY294002 or agonist ITS-X.

(C-D) Annexin V/PI assay (C) and cell cycle analysis using PI staining (D) in isotonic or hypertonic differentiated DE cells ($n > 5$ from 5 independent batches). The quantification of different cell cycle phases was calculated in FlowJo.

(E) RNA expression of *TRPV4* in ESCs and DE cells, determined by RNA-seq.

(F) Flow cytometric analysis showing cell size distribution treated with TRPV4 agonist or antagonist, as determined by FSC. Gates of small, middle and large cell size and quantification of gates percentage are indicated ($n = 3$ from 3 independent batches).

(G) The quantification of the proportion of CXCR4-positive cells assessed by flow cytometry, under hypertonic treatment added in different days.

(H) Hypotonic condition reduced the demand for Activin A during DE differentiation.

(I-J) RNA expression (I) and immunostaining analysis (J) of pancreatic (PDX1), intestinal (CDX2), and hepatic (AFP) lineages after further differentiating hypotonic DE cells.

(K) RNA expression and immunostaining analysis of pancreatic beta cells (Insulin, INS) differentiated from hypertonic DE cells.

(ns means not statistically significant, $*P < 0.05$, $**P < 0.01$, $***P < 0.001$)

Figure S4

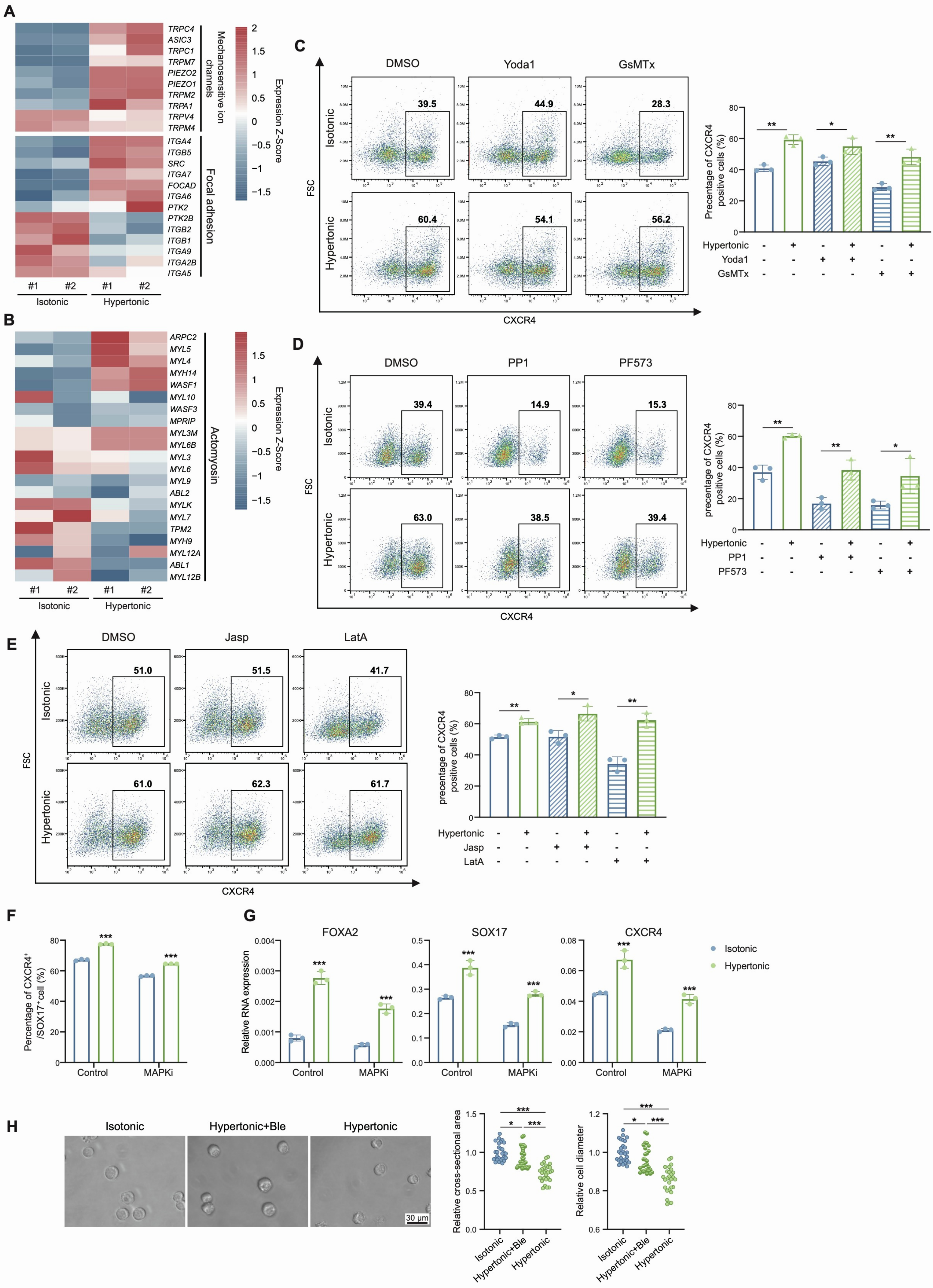

Figure S4. Cytoskeletal plays a role in endoderm differentiation boost caused by cell size decrease.

(A-B) Heatmap showing the expression of mechanosensitive ion channels, focal adhesion-related genes (A) and actomyosin cytoskeleton-related genes (B) within isotonic and hypertonic DE cells.

(C-E) Flow cytometry of CXCR4 expression in DE cells under isotonic or hypertonic culture differentiation condition with or without drug stimulations and quantification of CXCR4-positive cells proportion (n=3 from 3 independent batches). (C) 1μM Yoda1 (Piezo1 agonist) and 5μM GsMTx-4 (Piezo1 inhibitor), (D) 1μM PP1 (Src-family kinases inhibitor) and 1μM PF-573228 (focal adhesion kinase inhibitor), (E) 10nM Jasplakinolide (actin polymerization agonist) and 100nM Latrunculin A (actin polymerization inhibitor).

(F-G) DE differentiation efficiency determined by flow cytometric analysis of CXCR4 and SOX17 expression (F) and RNA expression of DE marker genes (G) in DE cells under isotonic or hypertonic pressure, treated with MAPK inhibitor.

(H) Representative images of ESCs in isotonic condition, hypertonic condition with or without Ble addition for 48h, and quantification of their cross-sectional area and diameter. (n > 28 from 3 independent batches cells per group analyzed, scale bar is 30 μm).

(ns means not statistically significant, *P < 0.05, **P < 0.01, ***P < 0.001).

Figure S5

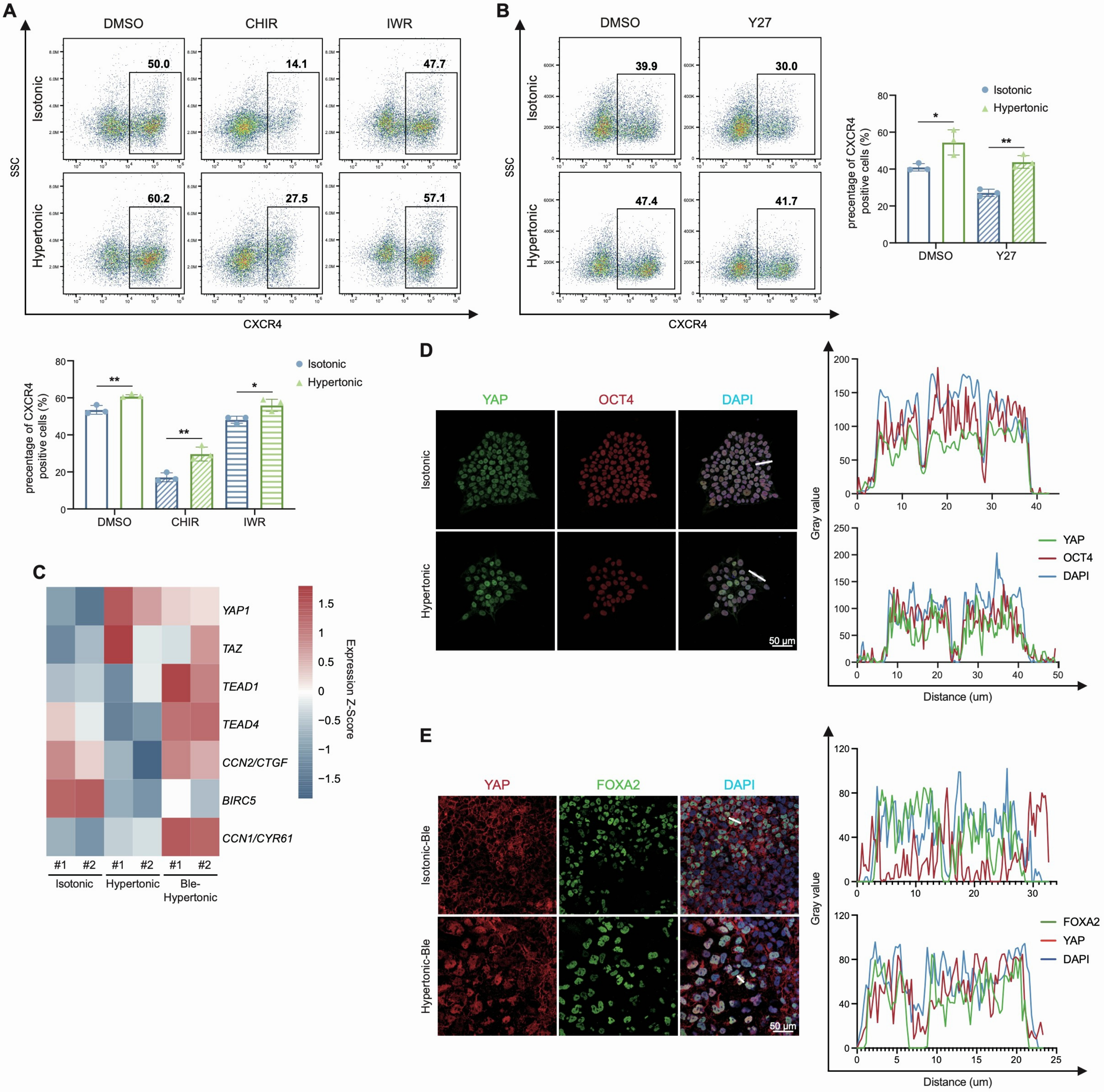

A
DMSO
CHIR
IWR
Isotonic
Hypertonic
SSC
CXCR4
50.0
14.1
47.7
60.2
27.5
57.1
B
DMSO
Y27
Isotonic
Hypertonic
SSC
CXCR4
39.9
30.0
47.4
41.7
Isotonic
Hypertonic
percentage of CXCR4 positive cells (%)
DMSO
Y27
precentage of CXCR4 positive cells (%)
DMSO
CHIR
IWR
C
YAP1
TAZ
TEAD1
TEAD4
CCN2/CTGF
BIRC5
CCN1/CYR61
Expression Z-Score
#1
#2
Isotonic
Hypertonic
Ble-Hypertonic
D
YAP
OCT4
DAPI
Isotonic
Hypertonic
50 μm
Gray value
Distance (um)
E
YAP
FOXA2
DAPI
Isotonic-Ble
Hypertonic-Ble
50 μm
Gray value
Distance (um)

Figure S5. Identification of cell size related signal pathway that contributes to hypertonic endoderm differentiation.

(A-B) Flow cytometric analysis of CXCR4 expression in DE cells under isotonic or hypertonic condition with or without drug stimulations and quantification of CXCR4-positive cells proportion as below (A) or right (B) (n=3 from 3 independent batches). (A) 2.5μM CHIR-98014 (WNT agonist) and 2μM IWR-1 (WNT inhibitor). (B) 10μM Y27632-2HCl (ROCK inhibitor).

(C) Heatmap showing the expression of YAP signaling-related genes in DE cells under isotonic or hypertonic condition with or without myosin inhibitor.

(D) Immunofluorescent labelling images of OCT4 and YAP in ESCs under hypertonic condition observed by confocal microscopy, with representative plots of fluorescent signals intensity along the white line for YAP (green), OCT4 (red) and DAPI (blue) (scale bar is 50 μm).

(E) Immunofluorescent labelling images of FOXA2 and YAP in DE cells under isotonic or hypertonic condition with Ble stimulations observed by confocal microscopy, with representative plots of fluorescent signals intensity along the white line for FOXA2 (green), YAP (red) and DAPI (blue) (scale bar is 50 μm).

(ns means not statistically significant, *P < 0.05, **P < 0.01)

Figure S6

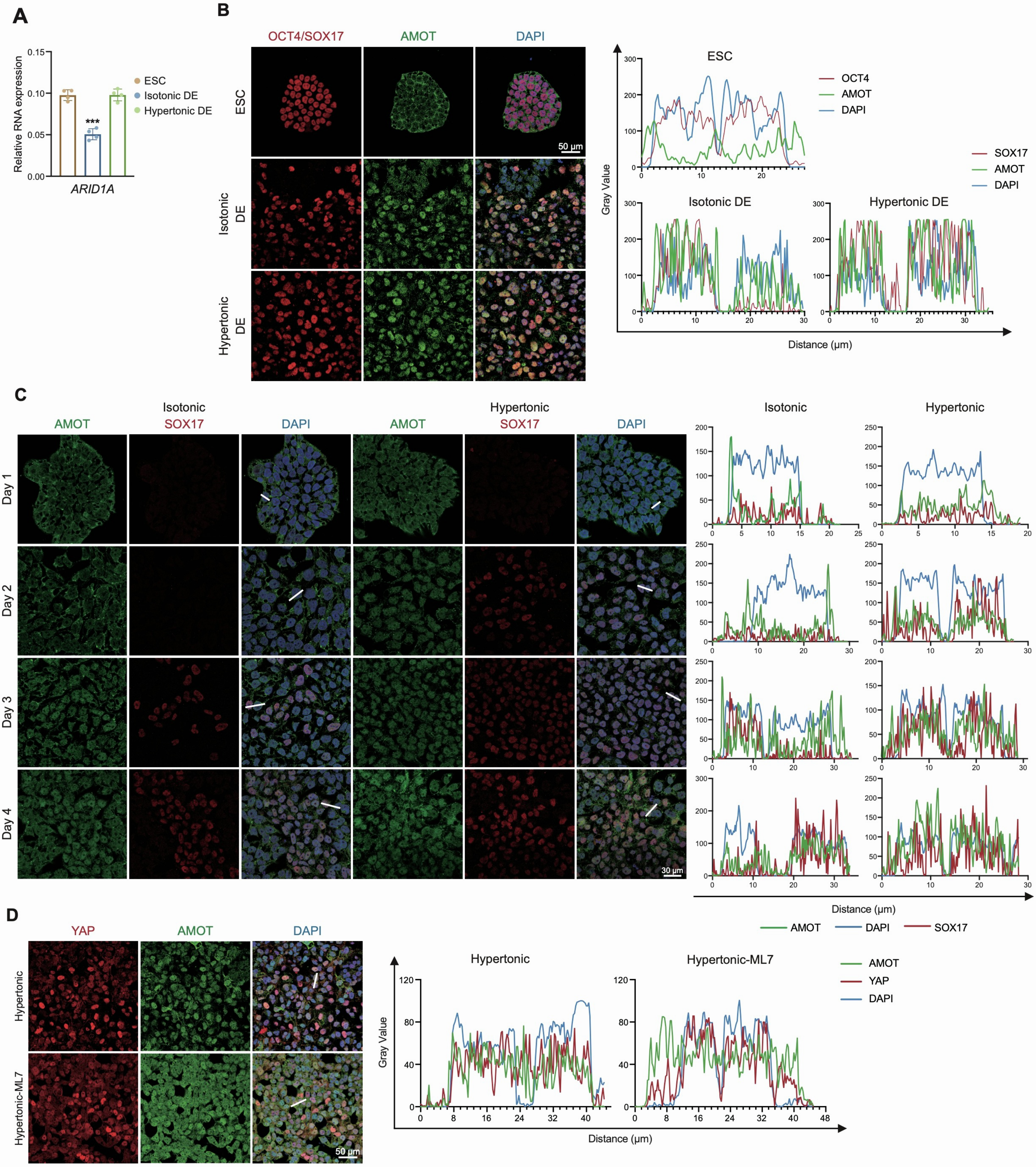
A
Relative RNA expression
ESC
Isotonic DE
Hypertonic DE
ARID1A
B
OCT4/SOX17
AMOT
DAPI
ESC
Isotonic DE
Hypertonic DE
50 μm
Gray Value
OCT4
SOX17
Distance (μm)
C
Isotonic
Hypertonic
Day 1
Day 2
Day 3
Day 4
30 μm
D
YAP
Hypertonic-ML7
Hypertonic
50 μm

Figure S6. Cell size compression promotes AMOT nuclear translocation.

(A) Relative mRNA expression of *ARID1A* in ESCs and DE cells under isotonic or hypertonic condition (n=4 from 4 independent batches). (***P < 0.001).

(B-D) Immunofluorescent labelling images were observed by confocal microscopy. Representative plots of fluorescent signals intensity along the white line. (B) AMOT nuclear translocation in ESCs and DE cells under isotonic or hypertonic condition (scale bar is 50 μm). (C) Time course AMOT nuclear translocation in H9 DE cells under isotonic or hypertonic condition (scale bar is 30 μm). (D) AMOT's co-localization with YAP and nuclear translocation in DE cells under hypertonic condition with or without myosin inhibition (scale bar is 50 μm).

Figure S7

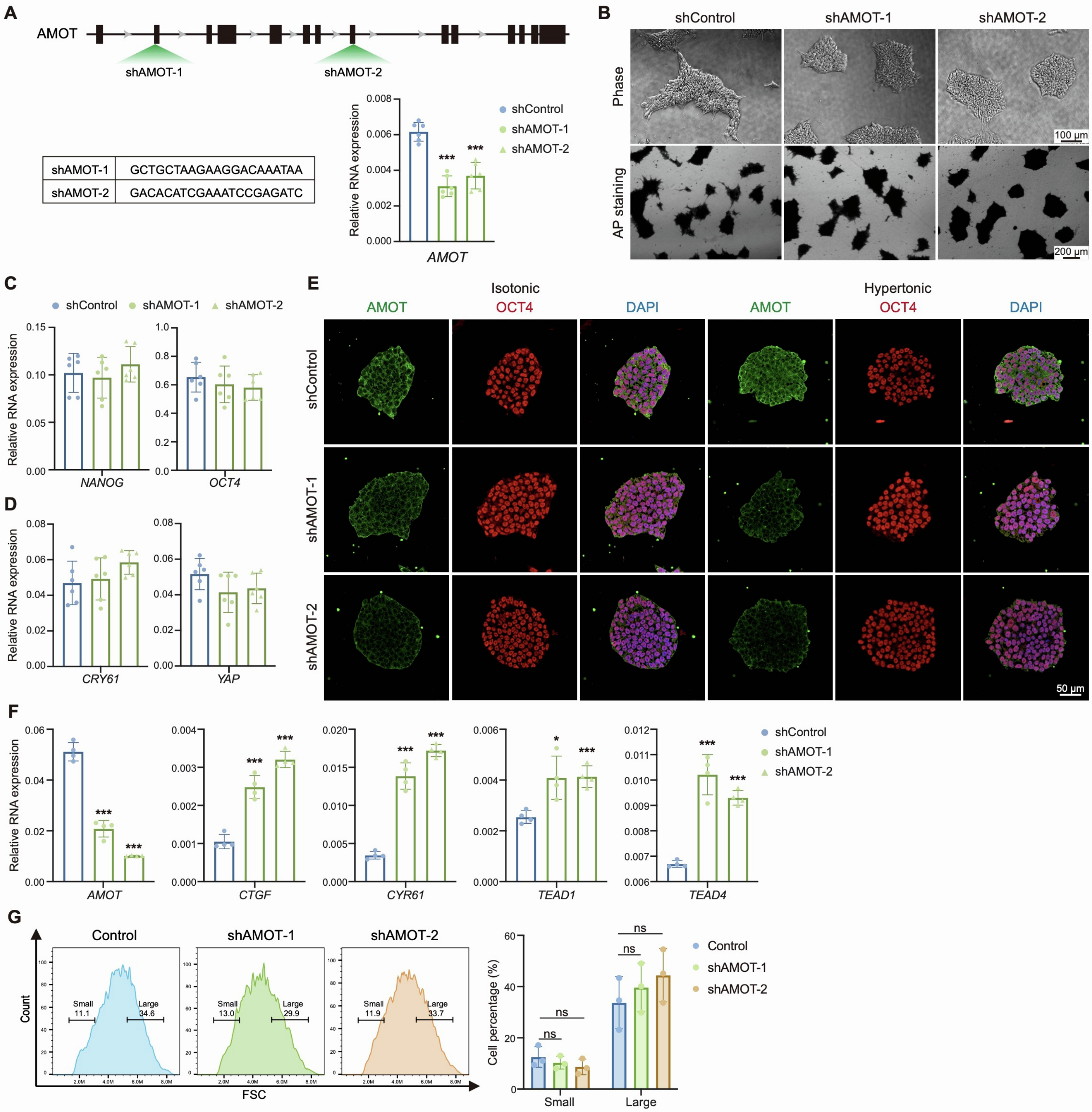

A
AMOT
shAMOT-1
shAMOT-2
shAMOT-1 GCTGCTAAGAAGGACAAATAA
shAMOT-2 GACACATCGAAATCCGAGATC
Relative RNA expression
AMOT
shControl
shAMOT-1
shAMOT-2
B
shControl
shAMOT-1
shAMOT-2
Phase
AP staining
100 µm
200 µm
C
NANOG
OCT4
D
CRY61
YAP
E
Isotonic
Hypertonic
AMOT
OCT4
DAPI
50 µm
F
CTGF
CYR61
TEAD1
TEAD4
G
Control
Count
FSC
Small
Large
Cell percentage (%)
ns

Figure S7. AMOT is not essential for pluripotency but influential for YAP activation.

(A) Illustration of designed shRNA target position and the measurement of knockdown efficiency of shControl and shAMOT in ESCs (n=6 from 6 independent batches).

(B) Phase-contrast images of shControl, shAMOT-1, shAMOT-2 ESCs colonies and bright-field images showing the AP staining of ESCs which has been cultured in isotonic or hypertonic medium for 48 hours (scale bar is 100 μm and 200 μm, respectively).

(C-D) Relative mRNA expression of pluripotency gene (C) and YAP target genes (D) in shControl and shAMOT ESCs (n=6 from 6 independent batches).

(E) Representative confocal images for AMOT (green) and OCT4 (red) as detected in shControl and shAMOT ESCs under hypertonic condition. Nuclei were counterstained with DAPI (blue) (scale bar is 50 μm).

(F) Relative mRNA expression of YAP target genes in shControl and shAMOT 293T.

(G) Cell size distribution of AMOT knockdown and control cells, determined by FSC.

(ns means not statistically significant, *$P < 0.05$, **$P < 0.01$, ***$P < 0.001$).

Supplementary Tables S1. Primer list.

Gene	Forward primer	Reverse primer
OCT4	CAAAGCAGAAACCCTCGTGC	TCTCACTCGGTTCTCGATACTG
SOX2	GTCATTTGCTGTGGGTGATG	AGAAAAACGAGGGAAATGGG
NANOG	CCCCAGCCTTTACTCTTCCTA	CCAGGTTGAATTGTTCCAGGTC
FOXA2	GGAGCAGCTACTATGCAGAGC	CGTGTTCATGCCGTTCATCC
SOX17	GCATGACTCCGGTGTGAATCT	TCACACGTCAGGATAGTTGCAGT
CXCR4	TACACCGAGGAAATGGGCTCA	AGATGATGGAGTAGATGGTGGG
T	GATGATCGTGACCAAGAACGG	CCACGAAGTCCAGCAGGAA
MIXL1	GAGACTTGGCACGCCTGT	GGTACCCCGACATCCACTT
AMOT	AGGGCGAGATTCGGAGGAT	CCTCTGACCCCTCATATTCCTT
ARID1A	TTATCTCCGCGTCAGCCTTC	ACTGGGGTAGTTGGCATTGG
YAP1	AAGAACTGCTTCGGCAGGTG	GCAGGGCTAACTCCTGACAT
TAZ	TCCCAGCCAAATCTCGTGATG	AGCGCATTGGGCATACTCAT
TEAD1	ATGGAAAGGATGAGTGACTCTGC	TCCCACATGGTGGATAGATAGC
TEAD4	CCGGTCGGAACGAGCTG	CCTTAGCTGCCTGGTCCTTT
CTGF	AGGAGTGGGTGTGTGACGA	CCAGGCAGTTGGCTCTAATC
CYR61	ACCGCTCTGAAGGGGATCT	ACTGATGTTTACAGTTGGGCTG
GAPDH	AATGAAGGGGTCATTGATGG	AAGGTGAAGGTCGGAGTCAA

EXPERIMENTAL PROCEDURES

METHODS DETAILS

Flow cytometric analysis

For endoderm differentiation analysis, the cultured cells were digested into single cells by TrypLE (Gibco, #12604021) for 3 minutes at 37°C and washed twice with DPBS containing 2% FBS. Then cells were incubated with 1:200 dilution of CD184 (CXCR4) -APC (BD, #555976) for 30 minutes, followed with intracellular antibody staining. Cells were fixed according to the manufacturer's instructions of Transcription Factor Buffer Set (BD, #562574) and then incubated with SOX17-Alexa488 (BD, #562205) antibody. Corresponding isotype was used as control. The SOX17-positive or CXCR4-positive cells were detected by NovoCyte flow cytometer (ACEA, USA) and analyzed by FlowJo software. For cell cycle analysis, a cell cycle detection kit (Keygen, China, Cat#KGA512) was used according to the protocol included in the kit. DE cells were dissociated into single cells and fixed with chilled anhydrous ethanol for 1 hour at room temperature. After washed with phosphate-buffered saline (PBS) twice, cells were resuspended with 500μL RNase A solution (100μg/ml RNase A) and incubated at 37 °C for 30 minutes. Then the cells were added with propidium iodide (PI) solution (50μg/ml PI) and incubated at room temperature for another 30 minutes in dark. Finally, a NovoCyte flow cytometer (ACEA, USA) was used to analyze these cells. The DNA content was analyzed on the basis of PI intensity in the FlowJo (v10.4.0), by which these sorted cells were divided into each cell cycle phase (subG1, G1, S, and G2/M) and calculated the proportion of each phase. For apoptosis analysis, an Annexin V-FITC/PI apoptosis detection kit (Keygen, China, Cat#KGA106) was used. DE cells were dissociated into single cells and added with Annexin V-FITC/PI, followed with incubation at room temperature for 15 minutes in dark. Cell apoptosis should be assessed by flow cytometer within 1 hour and later analyzed by FlowJo.

Immunofluorescence assay and alkaline phosphatase (AP) staining

For immunofluorescence assay, cells were fixed in 4% paraformaldehyde after PBS washing and blocked with blocking solution (DPBS with 10% (v/v) donkey serum and 0.3% Triton-100). Then cells were incubated overnight with the primary antibodies at proper concentration at 4 °C. The used primary antibodies in the experiment were: SOX17 (R&D, #AF1924, 1:200), FOXA2 (R&D, #AF2400, 1:200), OCT4 (CST, #2750, 1:200), NANOG (CST, #4903, 1:200), YAP1 (Abclonal, #A19134, 1:200), YAP1 (Abcam, # ab205270, 1:200), AMOT (Proteintech, #24550-1-AP, 1:200), Phospho-Myosin Light Chain 2 (Thr18/Ser19) Antibody (CST, #3674), Myosin IIa Antibody (CST, #3403). After washed with PBS for three times, cells were stained with secondary antibodies at room temperature in dark, then counterstained with 5 μg/ml 4',6-Diamidino-2-phenylindole dihydrochloride (DAPI, Sigma, Cat#10236276001) for 10 minutes. Cells were visualized and imaged using fluorescence microscopy (Olympus) or confocal microscope (Leica, Stellaris 5 WLL). Image analysis and image processing were performed using ImageJ/Fiji (NIH)(Schindelin et al., 2012). Co-localization was evaluated using the ImageJ to calculate Pearson's correlation or the RGB profiles tool to obtain the fluorescence intensity plots. For AP staining, the ESCs were cultured for at least 48 hours. The medium was discarded, and the ESC samples were washed three times with PBS, following AP staining experiments were performed according to manufacturer's recommendations (Beyotime, Cat#C3206). Briefly, cultured cells were fixed with 4% paraformaldehyde for 20 minutes, followed with washing three times with PBS and incubation in BCIP/NBT staining mix in dark for 3 hours. After staining reaction termination by PBS washing, colonies were visualized and photographed using microscopy (Olympus).

RNA extraction and RT-qPCR

Total RNA from cells was extracted with HiPure Total RNA Mini Kit (Magen, Cat#R4111-03) according to manufacturer's manual. 1 mg of total RNA was used for reverse-transcription to cDNA with the ABScript II RT Master Mix (ABclonal, Cat#RK20402). RT-qPCR was performed on a C1000 Touch Thermal Cycler machine

(Bio-Rad) using 2x SYBR Green qPCR Master Mix (Biomake, Cat#B21203). The relative gene expression levels were normalized to the level of *GAPDH* based on the delta Ct method. All RT-qPCR experiments were carried out at least three replicates. Comparison between samples was performed using Student's t test. The primers used in the RT-qPCR assays are listed in Table S1.

RNA-seq and data analysis

RNA was isolated with HiPure Total RNA Mini Kit and sent to Geekgene (Beijing, China) for RNA-seq library construction and sequencing on an Illumina Hiseq X Ten platform with paired-end reads. For data analysis, RNA-seq raw data that contained adapters were removed and trimmed by Trim Galore (v0.6.6). The clean reads were aligned to the human GRCh38 genome reference with the HISAT2 (v2.1.0), and gene expression counts were determined by featureCounts (v2.0.1). All counts were further normalized with TPM (Transcripts Per Million) in R software. The RNA-seq data of ESC and DE cells in Figure 1 were derived from our previous works(Lu et al., 2023; Yang et al., 2020; Zheng et al., 2021). To determine differential expression genes, DESeq2 (v1.30.1)(Love et al., 2014) was used to define significant differences genes by setting adjust p value <0.05 and abs (log_2(fold-change)) > 1.0. Gene Ontology analysis was executed by Profiler(Reimand et al., 2019) (https://biit.cs.ut.ee/gprofiler/gost), Venn diagram was made by Venn website (https://bioinformatics.psb.ugent.be/webtools/Venn/) and the heatmap was made by pheatmap (v1.0.12).

AMOT knockdown

We utilized shRNA for knockdown and the shRNAs specifically against *AMOT* and scramble control were cloned into lentiviral vector pLKO.1 plasmid (Addgene Plasmid 10878). For lentivirus packaging, the lentiviral shRNA plasmid and lentiviral helping vectors (psPAX2, pMD2.G) were transfected in HEK293T cells and the virus was concentrated. After lentivirus infection to human ESCs, 2 mg/mL puromycin was used for selection to establish stable shAMOT knockdown ESC line. The sequences of

oligoes were listed as Fig. S7A and below: shControl: GAAGTATTCCGCGTACGTT.

Statistical analysis

Statistical analysis was conducted using PRISM 9.5.1 for macOS. The results were shown as means ± SD from at least three independent experiments. Single comparison between two groups was analyzed by two-tailed unpaired t-test. Comparisons between multiple groups were determined using One-Way ANOVA analysis. Pearson's correlation analysis was used to evaluate the correlation between two variables. P value<0.05 is considered statistically significant (* means $p < 0.05$, ** means $p < 0.01$, and *** means $p < 0.001$), while "n.s." stands for not statistically significant.
