# Supplementary material for: Cell size regulates human endoderm specification through actomyosin-dependent AMOT-YAP signaling
Source: Stem Cell Reports. 2024 Aug 1;19(8):1137–55. doi: 10.1016/j.stemcr.2024.07.001 (PMC11368700; doi:10.1016/j.stemcr.2024.07.001)
Supplement: Document S2. Article plus supplemental information [file mmc2.pdf]

Stem Cell Reports
Article
OPEN ACCESS

Cell size regulates human endoderm specification through actomyosin-dependent AMOT-YAP signaling

Lai Jiang,[1,2] Chenchao Yan,[2] Ying Yi,[2] Lihang Zhu,[2] Zheng Liu,[3] Donghui Zhang,[1,*] and Wei Jiang[2,4,5,*]
[1]State Key Laboratory of Biocatalysis and Enzyme Engineering, School of Life Science, Hubei University, Wuhan 430062, China
[2]Department of Biological Repositories, Frontier Science Center for Immunology and Metabolism, Medical Research Institute, Zhongnan Hospital of Wuhan University, Wuhan University, Wuhan 430071, China
[3]The Institute for Advanced Studies, Wuhan University, Wuhan, China
[4]Hubei Provincial Key Laboratory of Developmentally Originated Disease, Wuhan, China
[5]Lead contact
*Correspondence: dongh.zhang@hubu.edu.cn (D.Z.), jiangw.mri@whu.edu.cn (W.J.)
https://doi.org/10.1016/j.stemcr.2024.07.001

SUMMARY

Cell size is a crucial physical property that significantly impacts cellular physiology and function. However, the influence of cell size on stem cell specification remains largely unknown. Here, we investigated the dynamic changes in cell size during the differentiation of human pluripotent stem cells into definitive endoderm (DE). Interestingly, cell size exhibited a gradual decrease as DE differentiation progressed with higher stiffness. Furthermore, the application of hypertonic pressure or chemical to accelerate the reduction in cell size significantly and specifically enhanced DE differentiation. By functionally intervening in mechanosensitive elements, we have identified actomyosin activity as a crucial mediator of both DE differentiation and cell size reduction. Mechanistically, the reduction in cell size induces actomyosin-dependent angiomotin (AMOT) nuclear translocation, which suppresses Yes-associated protein (YAP) activity and thus facilitates DE differentiation. Together, our study has established a novel connection between cell size diminution and DE differentiation, which is mediated by AMOT nuclear translocation. Additionally, our findings suggest that the application of osmotic pressure can effectively promote human endodermal lineage differentiation.

INTRODUCTION

Cell size is a key characteristic of cell state that undergoes changes throughout the cell's lifespan, such as enlarging during cell senescence (Lengefeld et al., 2021) and cell growth (Fingar et al., 2002). Furthermore, cell size can change rapidly on shorter time scales, for example, during cell cycle (Cadart et al., 2022) and cell spreading (Guo et al., 2017). Importantly, reducing the size of large cells can rescue their senescence, while inhibiting cell enlargement can suppress cell cycle (Lengefeld et al., 2021; Cadart et al., 2022), indicating that cell size actively regulates multiple biological functions. Cell size is intrinsically related to the mechanical state and physical properties of cells. External control of cell size can be achieved through physical stimuli, such as osmotic pressure and substrate stiffness, as well as chemicals, including various ion channel regulators (Liu et al., 2020; Venkova et al., 2022). Cell shrinkage leads to membrane folding, reducing cell plasma membrane tension and cell elasticity with increasing Young's modulus (Guo et al., 2017; Roffay et al., 2021). In addition, cell size diminution could induce phase-separated biomolecular condensates through molecular crowding, as reported in regulating WNT and HIPPO pathways (Li et al., 2021b; Wang et al., 2022). Interestingly, different cell types from the same organism can exhibit significantly different volumes. For example, pancreatic β cells are surrounded by acinar cells that are roughly twice their size (Auffret et al., 2013), and this disparity can be utilized as a strategy for cell sorting (Smejkal et al., 2023).

Cell volume undergoes changes during embryonic development and organogenesis. The oocyte undergoes maturation, accompanied by increased osmotic pressure and activation of intracellular calcium signals, during its transition from the ovary to the uterus (Horner and Wolfner, 2008). In the earliest preimplantation embryos, cell size continuously decreases from the zygote to the blastocysts. During this stage, the embryonic cells divide without increasing in volume (Courtois et al., 2012). In the blastocyst stage, cells actively create an osmotic gradient to generate luminal pressure. This pressure stretches and compresses trophectoderm cells, thereby determining their fate (Chan et al., 2019). In addition, neurogenesis from neural stem cells is strongly promoted in soft extracellular matrix with large cell size but suppressed by cell size compression (Baek et al., 2022); intestinal stem cells have small cell size and conical shape, distinct from their differentiating derivates. Interestingly, these unique cellular characteristics activate the WNT signaling pathway by regulating molecular crowding and niche interactions, further enhancing their stemness (Li et al., 2021b; Pentinmikko et al., 2022). These findings raise the hypothesis that cell size might actively participate in cell fate determination. However, it is still unknown whether cell size changes and contributes to human early embryonic germ layer differentiation. Human embryonic stem cells (ESCs) are derived from human

This is an open access article under the CC BY license (http://creativecommons.org/licenses/by/4.0/).

blastocysts and can self-renew indefinitely *in vitro* while retaining the potential to differentiate into the three germ layers (Thomson et al., 1998). Therefore, the *in vitro* differentiation of human ESCs provides a valuable tool to investigate the functional relationship between cell size and early germ layer differentiation.

In this study, we utilized the directed definitive endoderm (DE) differentiation system, which gives rise to respiratory and digestive epithelium, as well as the thyroid, thymus, liver, and pancreas (Wells and Melton, 1999), to investigate the cell size dynamics and dissect the functional link between cell size and differentiation. Our findings revealed that during DE differentiation from human ESCs, there was a decrease in cell size, and applying hypertonic pressure or chemical to induce cell compression promoted DE differentiation. In addition, we observed that the mechanosensitive actomyosin played a role in hypertonic cell compression and mediated the boosted DE differentiation. Furthermore, we explored the underlying mechanism and identified that Yes-associated protein (YAP)-angiomotin (AMOT) co-localization under hypertonic pressure contributed to DE differentiation by suppressing the YAP pathway.

RESULTS

DE differentiation is accompanied by a diminution of cell size and change in cell mechanical state

Since it has not yet been determined whether cell fate commitment during embryonic germ layer development is associated with changes in cell size, we utilized DE differentiation of human ESCs to investigate the dynamics of cell size. We initially examined the size of ESCs and differentiated DE cells under microscopy and observed a decrease in the cell size after endoderm differentiation (Figure 1A). The result from Coulter counter also clearly showed that DE cells exhibited smaller cell size than ESCs in suspension condition (Figure S1A). Since the forward scatter (FSC) value determined by the flow cytometer was able to reflect the cell size (Pentinmikko et al., 2022), we performed the same assay and observed that the size of DE cells was significantly smaller than that of ESCs (Figure 1B). We further analyzed the time-course size distribution by categorizing cells into small, middle, and large groups based on FSC gating. The results revealed a gradual increase in the proportion of cells with small size and a decrease in the proportion of cells with large size (Figure 1C), indicating a decrease in cell size over time during DE induction. In addition, we calculated the cell volume and surface area by segmenting time-lapse three-dimensional (3D) confocal stacks of GFP-labeled cells. The result showed that DE cells were statistically significantly smaller compared to ESCs (Figure 1D).

ESCs with a high proliferation rate exhibit increased G2/M phase, likely contributing to the larger size. To investigate this possibility arising from differences in the cell cycle phases, we compared the cell size of DE and ESC in the same state. We indeed observed a significant difference in the cell cycle phases between ESCs and DE cells, with ESCs exhibiting a higher proportion of G2/M phase compared to DE cells (Figure S1B). However, the size of DE cells during the G0/G1 and G2/M phases was smaller than that of ESCs in the corresponding phases (Figure S1B). These data demonstrate that the existence of a cell size difference between ESCs and DE cells cannot be attributed to disparities in the cell cycle states. Additionally, we investigated the alterations in cell size during mesoderm and ectoderm differentiation as a control for our endoderm differentiation. All three cell types were derived from human ESCs and exhibited lower proliferation rates compared to ESCs. Our data indicated that the cell size of mesoderm was slightly smaller than that of ESCs, while the cell size of ectoderm was significantly larger (Figure S1C). These variations in cell size among endoderm, mesoderm, and ectoderm could also support that the varying proliferation rates are not the major cause, at least, of cell size change during lineage differentiation.

Cell size is intrinsically related to cell mechanical state, including cell stiffness, membrane tension, and other parameters such as molecular crowding (Guo et al., 2017; Li et al., 2021b). Additionally, mechanosensitive ion channels are associated with cell susceptibility to various forms of mechanical forces, as well as the processes of cell-autonomous osmotic pressure regulation and cell volume change (Fang et al., 2021). Thus, we first examined the expression of integrin, focal adhesion, actomyosin cytoskeleton and mechanosensitive ion channel-related genes in ESCs and DE cells (Figures 1E and 1F). We found that ESCs and DE cells expressed different types of integrin, indicating that they exhibited different mechanical states. Integrin β1 has been reported to participate in the cell's response to a soft matrix and it was highly expressed in ESCs. In contrast, α5 integrin signaling is known to monitor cells with a high mechanical state (Lv et al., 2015), which showed higher expression in DE cells (Figure 1E). Meanwhile, the expression levels of most focal adhesion, actomyosin and mechanosensitive ion channel-related genes were significantly different between ESCs and DE cells (Figure 1F). To further assess the difference of integrin tension between ESC and DE cells, we used the reversible shearing DNA-based tension probe (Li et al., 2021a). The results indicated that the tension signal in ESCs was diffuse and their fluorescence intensities of 56-pN were significantly lower. In DE cells, the tension signal was localized at a large area of cell edge and a clear 56-pN tension signal was observed (Figure S1D). The quantification of the 56-pN/12-pN tension signal indicated that the integrin

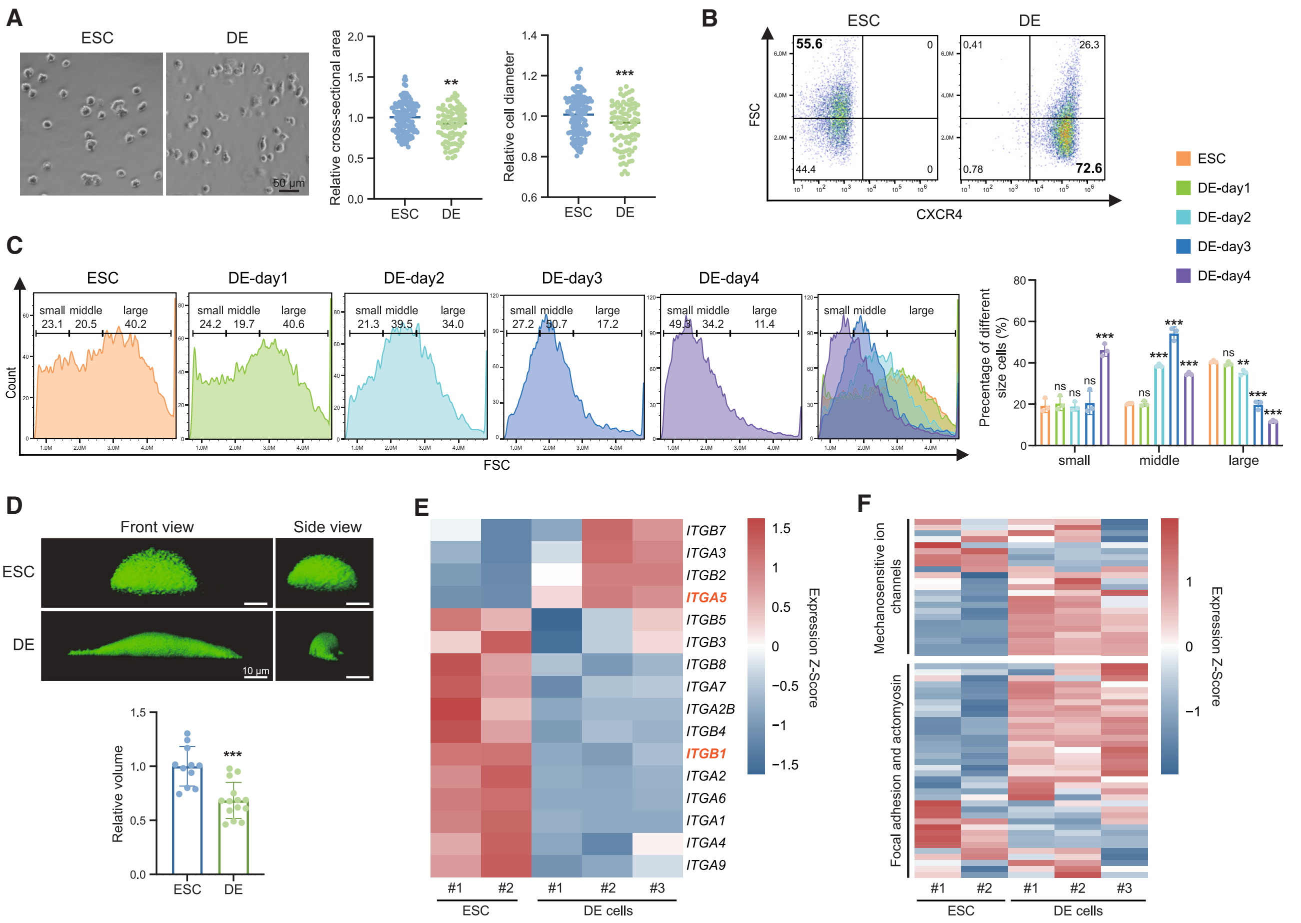

Figure 1. Human definitive endoderm differentiation is accompanied by changes in cell size and cell mechanical state

(A) Representative images of suspended ESCs and DE cells and quantification of their cross-sectional area and diameter ($n > 85$ cells from 3 independent batches per group analyzed, scale bar is 50 μm).

(B) Flow cytometric analysis of CXCR4 in ESCs and DE cells. Size distribution of cells as determined by forward scatter (FSC).

(C) Flow cytometric analysis of the time-course size distribution of ESCs and DE cells as determined by FSC. Gates of small, middle, and large cell size and time-course (day 0–4) quantification of gates percentage during endoderm differentiation are indicated ($n = 3$ from 3 independent batches).

(D) Representative 3D confocal images of GFP-labeled ESCs and DE cells, and quantification of their volume ($n > 11$ cells from 3 independent batches per group analyzed, scale bar is 10 μm).

(E and F) Heatmap showing the expression of cell volume and cell mechanical state-related genes within ESCs and DE. (E) Heatmap showing the higher expression of *ITGA5* in DE cells, which marks cell high mechanical state; the lower expression of *ITGB1* in DE cells, which participates in cell response to soft matrix (ns means not statistically significant, $**p < 0.01$, $***p < 0.001$).

tension to the substrate in DE cells is much stronger than ESCs. Overall, these observations demonstrate that human DE differentiation is accompanied by a decrease in cell size, a specific mechanical state with stronger mechanical tension, and an increase in cell stiffness.

Cell size diminution contributes to endoderm differentiation

To address whether smaller cells are more competent to endoderm differentiation, we manipulated cell size externally using hypertonic pressure. Hypertonic pressure treatment rapidly and consistently reduces cell volume within a few seconds and for the subsequent period (Guo et al., 2017; Lee et al., 2019; Li et al., 2021b; Roffay et al., 2021). We added sucrose or PEG300 to the medium as hypertonic pressure for 48 h and observed a significant decrease in the volume of ESCs with increasing hypertonicity (Figures S2A–S2C). We also observed that GFP-labeled ESCs presented obviously smaller cell size after 30 min of osmotic pressure stimulation (Figure S2D). The results demonstrate that osmotic pressure is an effective and stable way to regulate cell volume as expected (Guo et al., 2017;

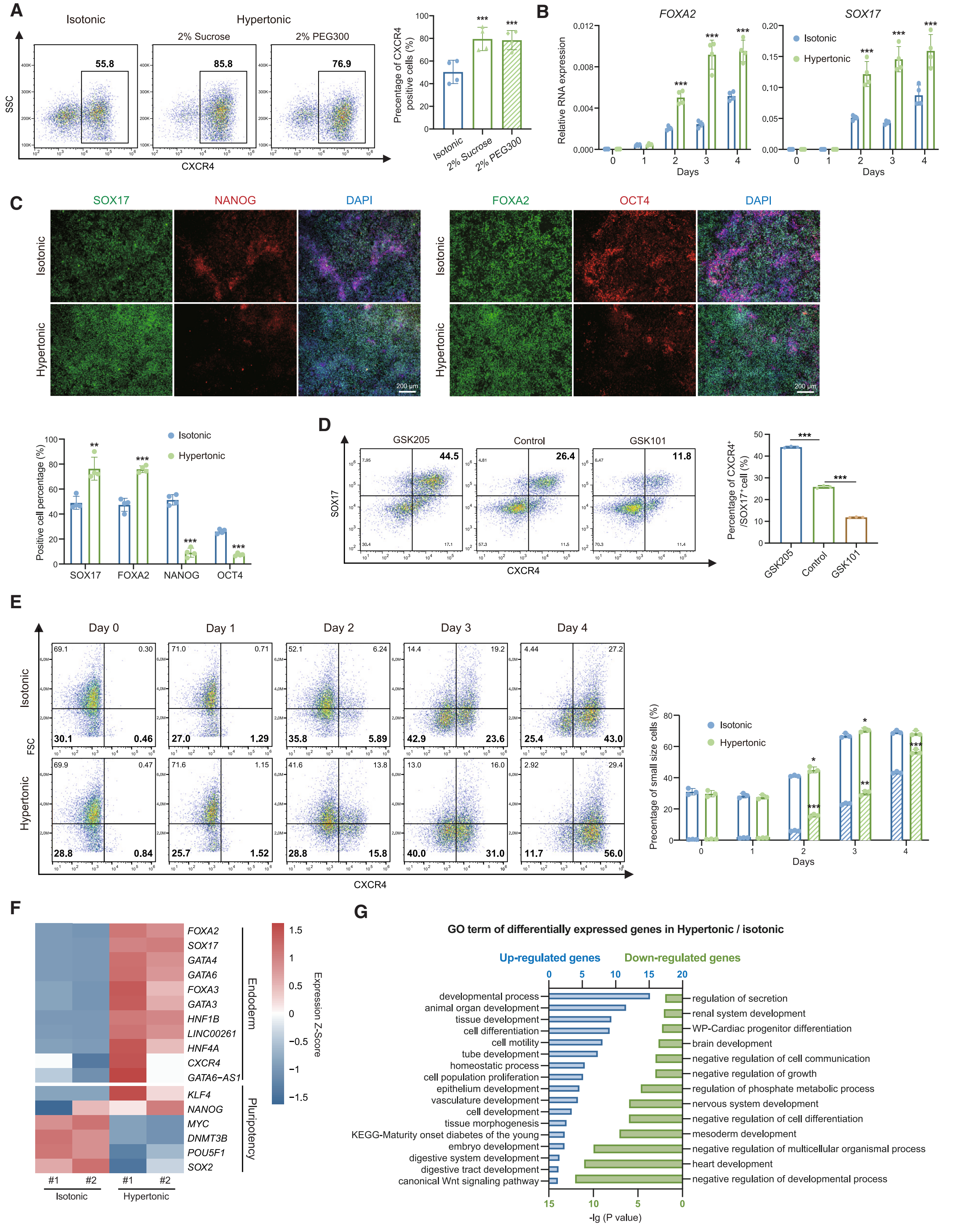

(legend on next page)

Lee et al., 2019; Li et al., 2021b; Roffay et al., 2021). Furthermore, under hypertonic treatment, human ESCs maintained good colony morphology and displayed positive alkaline phosphatase staining (Figure S2E).

Next, we performed DE differentiation under hypertonic condition and observed a significant increase in the percentage of CXCR4-positive DE cells compared to unperturbed DE cells in isotonic medium (Figure 2A). Moreover, the efficiency of DE differentiation increased with the degree of hypertonicity (Figures S2F and S2G). This was further supported by the analysis of the expression of endoderm markers, forkhead box A2 (*FOXA2*) and SRY-box transcription factor 17 (*SOX17*) (Figures 2B and 2C). Importantly, this phenomenon was observed in suspension differentiation systems as well (Figures S2H and S2I), and not impacted by the addition of PI3K inhibitor LY294002 or agonist insulin-containing medium (Figures S3A and S3B). Of note, the hypertonic pressure used in this study did not impact the apoptosis or cell cycle during DE differentiation (Figures S3C and S3D).

Moreover, we employed another approach to manipulate cell size, in addition to hypertonic treatment, and evaluated the DE differentiation. The transient receptor potential vanilloid-4 (TRPV4) ion channels regulate cell volume through balancing osmolality of calcium ions in the cytoplasm (Jo et al., 2015). The protein level of TRPV4 in MSCs was found to be diminished when cell volume expansion was restricted, such as increased osmotic pressure (Lee et al., 2019). By analyzing our RNA sequencing (RNA-seq) data (Jiang et al., 2015; Lu et al., 2023), we found TRPV4 was lower expressed in DE samples than in ESCs (Figure S3E), suggesting that TRPV4 might contribute to the cell volume change during DE differentiation. Since the TRPV4 agonist GSK101 increased the volume of cells, while cell volume expansion was significantly restricted by treatment with the TRPV4 antagonist GSK205 (Lee et al., 2019), we therefore applied the TRPV4 agonist and antagonist during DE differentiation of human ESCs. As expected, we found that the agonist GSK101 could efficiently enlarge cell volume, while the antagonist GSK205 could decrease cell size (Figure S3F). Most importantly, GSK205 enhanced DE induction and GSK101 reduced DE induction, measured by flow cytometric analysis of CXCR4 and SOX17 (Figure 2D). These data together with the hypertonic treatment support that cell size plays an important role in DE differentiation.

Next, we performed the time-course analysis of cell size distribution, showing that hypertonic pressure increased the percentage of cells with small size during endoderm differentiation, and CXCR4-positive cells appeared to have a more pronounced reduction in cell size (Figure 2E). Since the most significant reduction in cell size occurred between day 2 and day 3, we modified the timing of hypertonic treatment and discovered that hypertonic treatment on day 1–2 only could also effectively promote DE differentiation (Figure S3G). More importantly, we investigated whether manipulating cell size through hypertonic conditions could decrease the requirement for activin A, a widely used but costly growth factor. We reduced the concentration of activin A greatly to 10% (from 100 ng/mL to 10 ng/mL) and found that hypertonic conditions could efficiently induce DE formation at 10 ng/mL, comparable to the normal condition with 100 ng/mL activin A (Figure S3H), thereby reducing costs. Nevertheless, these results indicated that smaller cells were more prone to endoderm differentiation under hypertonic pressure.

To gain a better understanding of the impact of hypertonic pressure on endoderm differentiation, we conducted an RNA-seq experiment using isotonic and hypertonic DE cells. We found that hypertonic pressure downregulated the expression of key pluripotency genes while promoting endodermal gene expression (Figure 2F). Using a cutoff fold-change >2 and $p < 0.05$, we identified 1,675 differentially expressed genes between hypertonic and isotonic DE

Figure 2. Cell size diminution promotes endoderm differentiation

(A) Flow cytometric analysis of CXCR4 expression under isotonic or hypertonic DE differentiation condition and quantification of the DE differentiation efficiency marked as CXCR4-positive cells ($n = 4$ from 4 independent batches).

(B) Time-course analysis of mRNA expression of DE markers under isotonic or hypertonic differentiation condition ($n = 4$ from 4 independent batches).

(C) Immunostaining analysis of isotonic or hypertonic differentiated DE cells: DE markers SOX17 and FOXA2 (green); ESC markers: NANOG and OCT4 (red); and DNA (DAPI; blue) (scale bar is 200 μm). Quantifications of the positive staining shown as left-below.

(D) Intracellular flow cytometric analysis of CXCR4 and SOX17 of differentiated DE cells treated with TRPV4 antagonist GSK205, or agonist GSK101. Quantification was shown as right ($n = 3$ from 3 independent batches).

(E) Time-course analysis of cell size distribution during DE differentiation process ($n = 3$ from 3 independent batches). CXCR4-positive cells were used to mark DE cells and lower FSC marks small cells. Quantifications of small size cell percentage shown as right: hollow pillars indicate all the small size cells and filled pillars indicate the CXCR4-positive small size cells.

(F) The heatmap showed the differentially expressed DE and ESC marker genes in isotonic and hypertonic DE cells.

(G) Gene ontology analysis of upregulated (blue) and downregulated (green) genes in DE cells responding to hypertonic pressure (ns means not statistically significant, *$p < 0.05$, **$p < 0.01$, ***$p < 0.001$).

cells, with 1,212 genes upregulated in hypertonic DE cells. Many of the upregulated genes were associated to developmental processes, such as cell differentiation, tube development and epithelium development (Figure 2G). Notably, the upregulated genes were linked to digestive system development and maturity onset diabetes of the young (MODY), which are critical processes in the later stages of endoderm development. Meanwhile, the downregulated genes in hypertonic DE cells were significantly associated with the negative regulation of developmental process, mesoderm development, and nervous system development (Figure 2G). These results indicate that the reduction in cell size caused by hypertonic pressure specifically promotes the differentiation of human ESCs toward the endodermal fate.

To determine the impact of modulating cell size on endodermal developmental competence, we employed the same protocol to induce isotonic DE and hypertonic DE into endodermal lineages. We first observed that hypertonic DE cells exhibit higher expression levels of markers associated with pancreatic (PDX1), intestinal (CDX2), or hepatic (AFP) lineages (Figures S3I and S3J). Based on our previously developed protocol for differentiating pancreatic beta cells (Jiang et al., 2015; Tan et al., 2019), we further evaluated the beta cell differentiation competence of hypertonic DE cells. The results indicated that hypertonic DE cells were able to differentiate into insulin-positive β-like cells, similar to the isotonic DE cells (Figure S3K). Taken together, these data collectively demonstrate that treatment with hypotonic condition does not impact the developmental competence.

Actomyosin plays a role in endoderm differentiation boost caused by cell size diminution

Mechanosensitive ion channels, focal adhesion, and the actomyosin cytoskeleton are major factors that respond to changes in cell volume (Syeda et al., 2016; Hoffmann et al., 2009; Venkova et al., 2022). Since ESCs and DE cells have different expression levels of mechanosensitive ion channels, focal adhesion, and actomyosin-related genes (Figures 1E and 1F), we conducted an analysis of these genes in isotonic and hypertonic DE cells by RNA-seq. The results showed that most genes related to mechanosensitive ion channel were upregulated under hypertonic condition, while no consistent changes were observed in genes related to focal adhesion and the actomyosin cytoskeleton (Figures S4A and S4B). To investigate the effect of volume compression on DE differentiation, we applied agonists and antagonists of these targets during the differentiation process. We observed that the activation of Piezo1 was necessary for DE differentiation, while manipulating Piezo1 activity through either activator Yoda1 or inhibitor GsMTx-4 did not affect the enhancement of differentiation caused by hypertonic pressure (Figure S4C). Though inhibiting focal adhesion kinase (FAK) by PF-573228 or Src-family kinase by PP1 both hindered DE differentiation, they did not affect the influence of hypertonic pressure (Figure S4D). Additionally, we observed that actin polymerization was required for DE differentiation and hypertonic pressure could counteract the suppression of DE differentiation caused by actin polymerization inhibitor latrunculin A (Figure S4E). However, the actin polymerization agonist jasplakinolide did not further promote DE differentiation, suggesting that actin polymerization was already functionally saturated for DE differentiation and not the cause of hypertonic DE fate promotion. We also checked the effect of hypertonic treatment upon mitogen-activated protein kinase (MAPK) inhibition. Our data showed that inhibiting MAPKs could reduce the efficiency of DE differentiation as expected (Lau et al., 2023; Loh et al., 2014; Yap et al., 2014), but it did not affect the promoting effect of hypertonic treatment on DE differentiation (Figures S4F and S4G). These data do not support that osmotic stress impacts DE differentiation through MAPK.

Interestingly, the abolishment of actomyosin contractility using myosin inhibitor blebbistatin and ML-7, or its enhancement via treatment with calyculin A, was found to suppress or promote DE differentiation, respectively (Figure 3A). Meanwhile, all three myosin inhibitors or activators used were able to eliminate the promotion of endoderm differentiation by hypertonic pressure. This is confirmed by analyzing the expression of the endoderm markers (Figures 3B and 3C). This result indicates that myosin activity plays an active role in boosting endoderm differentiation under hypertonic pressure. In addition, we stained cells with fluorescently labeled phalloidin, which binds to F-actin, and with antibodies against non-muscle myosin IIA (NMMIIA) or phospho-myosin light chain (p-MLC), a marker of the activated form of myosin (Rosowski et al., 2015). The organization of actin in hypertonic cells showed no obvious difference from isotonic cells. However, hypertonic treatment increases the co-localization of NMMIIA and actin (Figure 3D), indicating a more mature actomyosin structure. Interestingly, elevated p-MLC content was accompanied by strong co-localization of activated myosin and actin in hypertonic-treated cells (Figure 3D), suggesting enhanced actomyosin activity under hyperosmotic conditions. In addition, the diminution in cell size under hypertonic treatment could be partially relieved by treatment with myosin inhibitors, as evidenced by microscopy and flow cytometry analysis (Figures 3E and S3H). These results suggest that the decrease in cell size under hypertonic treatment is dependent on actomyosin activation (Koushki et al., 2023).

To investigate the crucial role of actomyosin in hypertonic DE differentiation and whether inhibiting actomyosin activity can reverse the transcriptomic changes in hypertonic DE cells, we performed an RNA-seq experiment

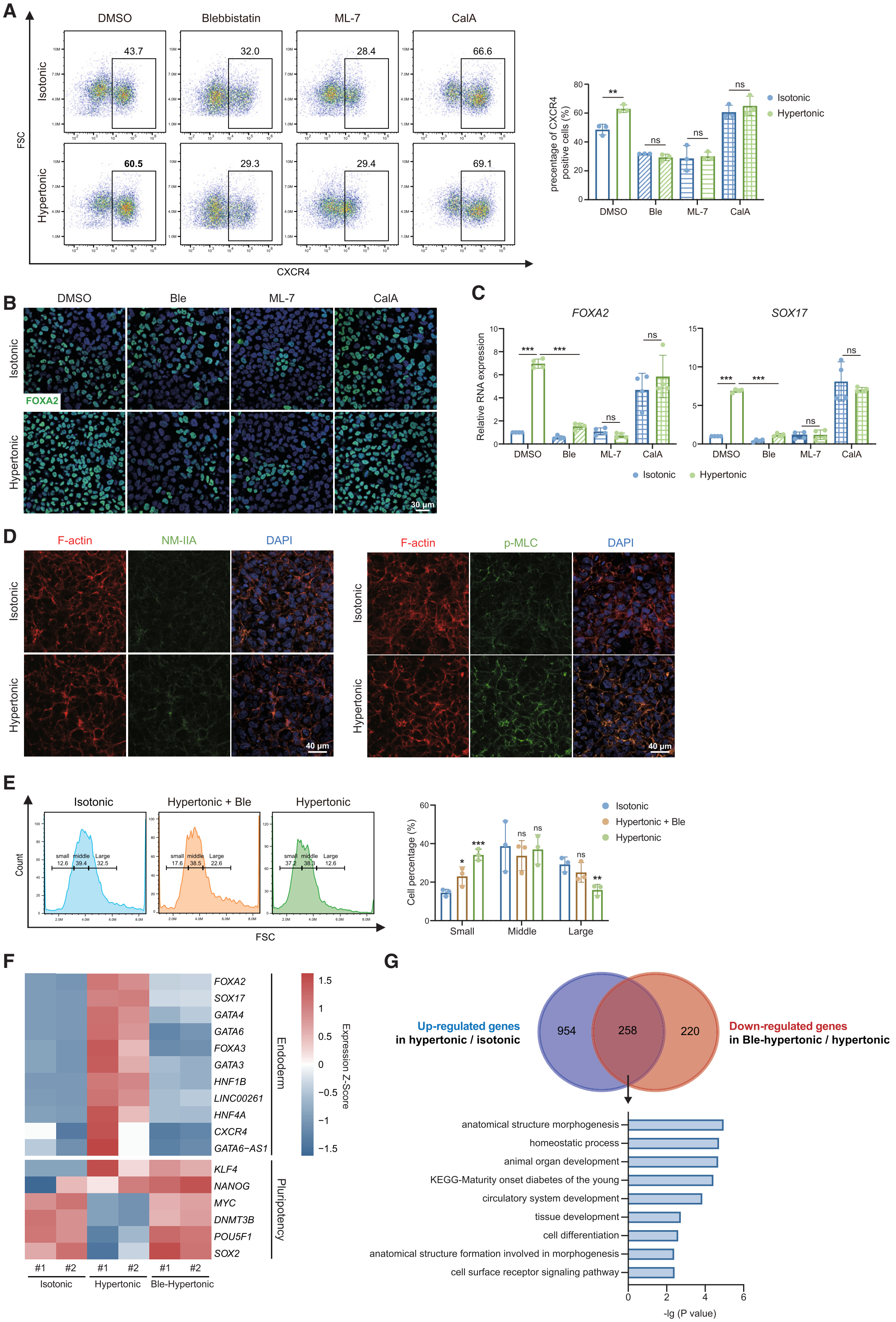

(legend on next page)

using isotonic, hypertonic, and Ble-treated hypertonic DE cells. Actomyosin inhibition rescued the decreased expression of the key pluripotent genes and increased the expression of endodermal genes induced by hypertonic pressure (Figure 3F). Using a cutoff fold-change >2 and $p < 0.05$, we identified 478 downregulated genes in Ble-hypertonic DE cells compared to cells treated with hypertonic solution alone. Interestingly, more than half of these downregulated genes (258/478) were found to be upregulated in hypertonic DE cells (1,212 genes) (Figure 3G). These overlapping genes were associated with developmental process, homeostatic process, and MODY (Figure 3G). These observations suggest that the enhancement of endoderm differentiation due to cell size decrease is largely dependent on the activation of actomyosin; moreover, the activation of myosin likely contributes to cell compression in hypertonic conditions.

YAP signal pathway participates in hypertonic endoderm differentiation

Since cell size reduction promotes DE differentiation from ESCs (Figure 2), our focus shifted to mechanosensitive signal pathways such as WNT/beta-catenin, Rho-associated protein kinase (ROCK), and YAP pathways. The WNT pathway, a crucial regulator of DE differentiation (Jiang et al., 2013b), is enhanced by intracellular crowding caused by cell volumetric compression (Li et al., 2021b). Rho/ROCK has been reported to control cell size (Tilly et al., 1996; Estevez et al., 2001; Sordella et al., 2002) and regulate gene expression (Taglietti et al., 2018; Yoshikawa et al., 2015). Thus, we used agonist and antagonist of these signaling pathways to determine their contribution to the hypertonic DE differentiation boost. We found that persistent activation of the WNT pathway restrained DE differentiation, and intervention in the WNT pathway did not affect the hypertonic differentiation boost (Figure S5A). Though inhibition of ROCK impeded DE differentiation, it failed to minimize the influence of hypertonic pressure (Figure S5B). These results indicated that the promotion of DE differentiation under hypertonic pressure was independent of the ROCK or WNT signaling pathway.

The YAP signaling pathway serves dual roles as a sensor and regulator of cell volume (Perez Gonzalez et al., 2018; Perez-Gonzalez et al., 2019; Pagliari et al., 2021; Sun et al., 2020). Thus, we applied Lats-IN-1 (YAP agonist) or verteporfin (YAP inhibitor), both of which abolished the hypertonic pressure-induced promotion of DE differentiation (Figure 4A). Importantly, YAP pathway suppression significantly enhanced DE lineage differentiation (Figures 4B and 4C). We hypothesized that hypertonic cell compression reduces YAP pathway activity. Through the analysis of gene expression related to the YAP pathway, we found that hypertonic pressure increased the expression of YAP and TAZ, while YAP target genes, such as *TEAD*, *CYR61*, and *BIRC5*, exhibited decreased expression under hypertonic pressure (Figure S5C). We conducted a further analysis of the temporal expression of YAP target gene *CYR61* and *CTGF* (Price et al., 2021). The result showed that the activity of YAP pathway decreased during DE differentiation and hypertonic pressure further accelerated this downregulation (Figure 4D). Moreover, inhibition of myosin partially reversed the suppressive effect of hypertonic pressure on YAP activity (Figure S5C). Given that YAP activity correlates with its intracellular localization (Nishioka et al., 2009), we observed nuclear YAP localization in ESCs and cytoplasmic localization in DE cells under isotonic conditions (Figures 4E and S5D). Unexpectedly, hypertonic treatment induced partial nuclear localization of YAP protein in DE cells but had minor effect on YAP localization in ESCs. This nuclear translocation of YAP caused by hypertonic pressure was not insensitive to actomyosin inhibition (Figure S5E), indicating a high sensitivity to cell size reduction independent of the cytoskeleton. Therefore, we hypothesized that a YAP repressor, responsive

Figure 3. Actomyosin plays a role in endoderm differentiation boost caused by cell size diminution

(A) Quantitative flow cytometric analysis of CXCR4 expression in DE cells in isotonic or hypertonic medium with or without myosin-related chemicals ($n = 3$ from 3 independent batches). 10 μM blebbistatin (myosin inhibitor), 2 μM ML-7 hydrochloride (myosin inhibitor), or 0.5 nM calyculin A (myosin agonist) was used.

(B) Immunostaining of FOXA2 (green) showing DE cells in isotonic or hypertonic medium with or without myosin-related chemicals (DAPI; blue) (scale bar is 30 μm).

(C) Relative mRNA expression of DE marker genes ($n = 4$ from 4 independent batches).

(D) Immunostaining of fluorescently labeled phalloidin (red, marking F-actin) and non-muscle myosin IIA (NMMIIA) or phospho-myosin light chain (p-MLC) of isotonic or hypertonic differentiated DE cells (scale bar is 40 μm).

(E) Size distribution of ESCs treated with or without blebbistatin was determined by FSC. Gates of small, middle, and large cell size and quantification of gates percentage are indicated ($n = 3$ from 3 independent batches).

(F) The heatmap showed the differentially expressed DE and ESC marker genes after differentiation in isotonic or hypertonic medium with or without blebbistatin.

(G) Venn diagram showing the overlap of upregulated genes in hypertonic condition (compared with isotonic DE cells) and downregulated genes in Ble-hypertonic condition (compared with hypertonic DE cells). GO analysis of these overlap genes (ns means not statistically significant, *$p < 0.05$, **$p < 0.01$, ***$p < 0.001$).

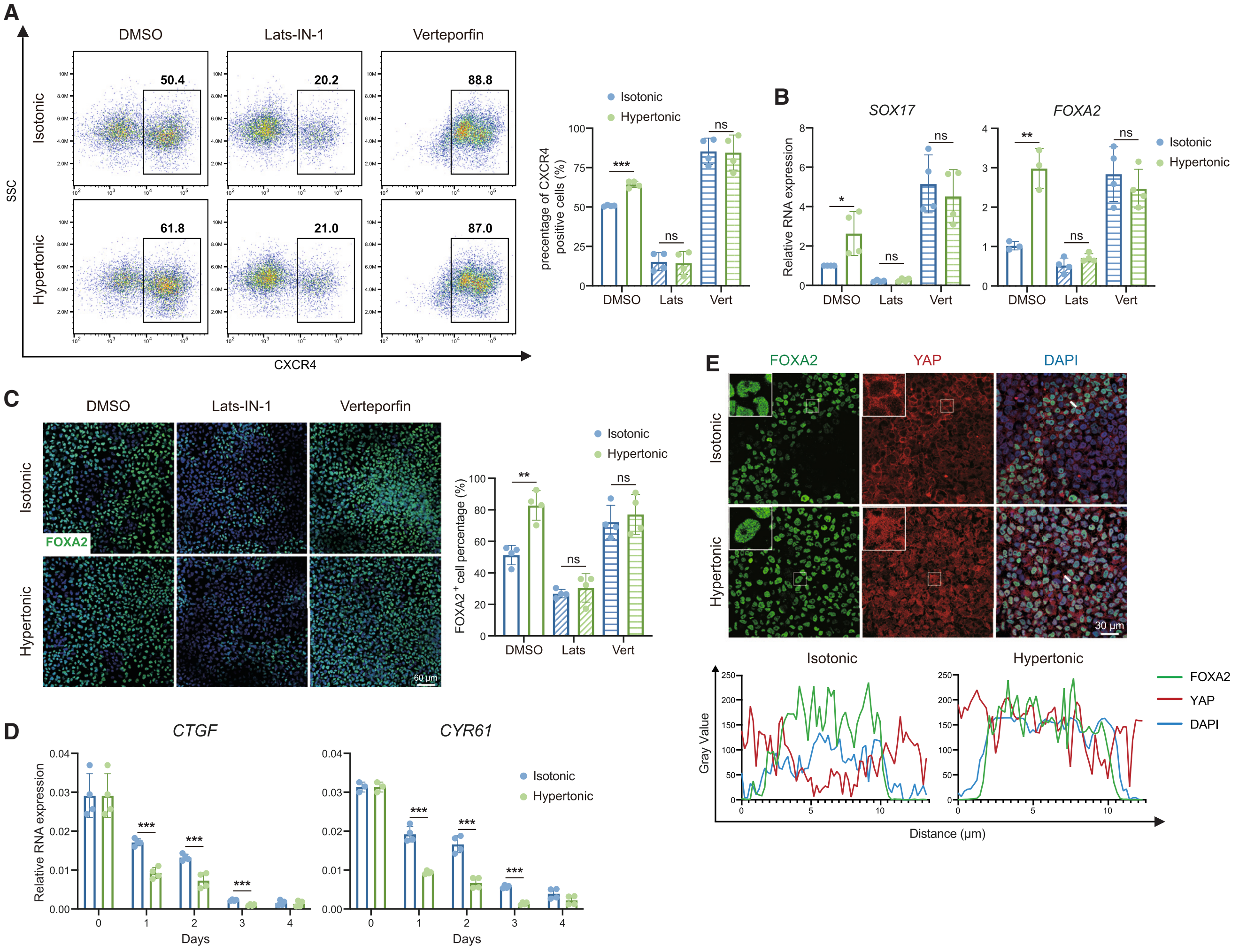

Figure 4. YAP signal pathway participates in the hypertonic endoderm differentiation boosting

(A) Quantitative flow cytometric analysis of CXCR4 expression in DE cells in isotonic or hypertonic medium with or without YAP-related chemicals (n = 4 from 4 independent batches). 1–10 μM Lats-IN-1 (YAP agonist) or 0.1–1 μM verteporfin (YAP inhibitor).

(B) Relative mRNA expression of DE marker genes (n = 4 from 4 independent batches).

(C) Immunostaining of FOXA2 (green) showing DE cells in isotonic or hypertonic medium with or without YAP-related chemicals (DAPI; blue) (scale bar is 60 μm). Quantifications of the positive staining shown as right.

(D) Time-course analysis of YAP target genes expression during isotonic or hypertonic DE differentiation (n = 4 from 4 independent batches).

(E) Confocal analysis of immunofluorescent images of YAP and FOXA2 in isotonic or hypertonic DE cells. Representative plots of fluorescent signals intensity along the white line for FOXA2 (green), YAP (red), and DAPI (blue) (scale bar is 30 μm) (ns means not statistically significant, *$p < 0.05$, **$p < 0.01$, ***$p < 0.001$).

to cell compression through actomyosin, may suppress YAP activity in the nucleus.

Cell size diminution disrupts YAP activation via promoting AMOT nuclear translocation

We then investigated the YAP repressor that is associated with hypertonic pressure during DE differentiation. From the literature, we identified two well-known YAP repressors, ARID1A and AMOT. The nuclear actin-binding factor ARID1A can sequester YAP away from TEADs and inhibit its activity (Chang et al., 2018). However, the expression of *ARID1A* was downregulated during DE differentiation from ESCs and upregulated under hypertonic pressure (Figure S6A). This suggests that ARID1A is not the suppressor of YAP activity under hypertonic pressure. AMOT inhibits YAP activity through directly interacting with YAP or regulating Lats1/2 activation in cytoplasm. Additionally, AMOT can directly bind with YAP in nucleus (Wang

et al., 2015; Nakajima et al., 2017). Interestingly, AMOT was reported to be involved in neural differentiation and mesoderm specification (Zaltsman et al., 2019; Pagliari et al., 2021). We observed an increase in the mRNA level of *AMOT* during DE differentiation. Furthermore, AMOT exhibited a higher level after hypertonic pressure (Figure 5A). By confocal examination, we found that AMOT was localized in the cytoplasm of ESCs but in the nucleus of SOX17-positive DE cells, regardless of isotonic or hypertonic conditions (Figure S6B). Moreover, hypertonic stimulation increased the co-localization of AMOT and YAP in both ESCs and DE cells (Figure 5B). In DE cells, YAP translocated from the cytoplasm to the nucleus and achieved higher Pearson's correlation with AMOT after hypertonic stimulation (Figure 5C). These observations indicate that the compression of cell size caused by hypertonic pressure promotes the co-localization of AMOT and YAP in nucleus, which is accompanied by decreased YAP activity.

We hypothesized that hypertonic pressure promotes the nuclear localization of AMOT, which in turn accelerates the inactivation of YAP signal pathway. To verify our hypothesis, we examined the temporal localization of AMOT during hypertonic DE differentiation. We observed that hypertonic pressure accelerated the nuclear localization of AMOT and the appearance of SOX17-positive DE cells (Figure 5D). This phenomenon was also observed in another human ESC line, H9 (Figure S6C). On day 2 after DE differentiation, the cytoplasmic localization of AMOT disappeared in the hypertonic treatment group, and nuclear localization began to occur, along with the appearance of SOX17-positive DE cells. This observation is highly consistent with the significant decrease in cell size on day 2 and the effective promotion of DE differentiation by hypertonic treatment only on day 1–2 (Figures 2E and S3G). To investigate the role of actomyosin in AMOT nuclear localization, we treated hypertonic DE differentiation with the actomyosin inhibitor ML-7. The result demonstrated that ML-7 effectively reduces the nuclear localization of AMOT, without significantly affecting the nuclear localization of YAP (Figure S6D). In addition, by assessing the expression of YAP target genes, we found that decreased co-localization of AMOT and YAP, caused by myosin inhibition, could activate YAP pathway under hypertonic condition (Figure S5C). Taken together, these observations demonstrate that the decrease in cell size promotes AMOT nuclear localization through actomyosin and increased the co-localization of AMOT and YAP, leading to reduced YAP signal activity.

To investigate the direct effect of AMOT on hypertonic DE differentiation, we used short hairpin RNA to target AMOT and established two human ESC lines with stable AMOT knockdown (Figure S7A). AMOT knockdown did not affect the colony morphology or expression of pluripotent genes and YAP-related genes in ESCs (Figures S7B–S7E), indicating that AMOT was not active in the undifferentiated status. Next, we subjected the AMOT knockdown ESCs to hypertonic pressure during DE differentiation. We found a significant decrease in the proportion of CXCR4-/SOX17-double-positive DE cells in hypertonic AMOT knockdown cells compared to control cells (Figure 6A). Furthermore, AMOT knockdown did not alter the localization of AMOT but significantly decreased the protein level of AMOT and SOX17 in DE cells (Figure 6B). These results indicated that the absence of AMOT could abolish the promotion of hypertonic DE differentiation, which is further supported by reverse-transcription PCR analysis of DE marker genes (Figure 6C). In addition, AMOT knockdown cells appeared to have increased YAP activity under hypertonic pressure (Figures 6D and S7F). Consistently, YAP inhibitors effectively rescued the decreased efficiency of DE differentiation caused by AMOT knockdown, suggesting that AMOT functions upstream of YAP (Figures 6E and 6F). In contrast, hypertonic treatment did not enhance the decreased DE induction caused by AMOT knockdown (Figures 6E and 6F), as AMOT functioned downstream of hypertonic treatment. Moreover, AMOT knockdown cells also exhibited smaller cell size (Figure S7G). In summary, these results demonstrate that the promotion of hypertonic DE differentiation relies on AMOT, which interferes with YAP activation.

Cell size and YAP signaling pathway are actively implicated in human early embryo development

Our present study reveals that cell size diminution influences DE induction through YAP signaling; however, whether it happens *in vivo* is unknown yet. To investigate this issue, we surveyed the single-cell RNA-seq dataset from 3D-cultured human pre-gastrulation embryos (GSE136447) (Xiang et al., 2020), which contains different cell types around gastrulation stage: embryonic disc, amnion, basement membrane, primary and primate unique secondary yolk sac, anterior-posterior polarity formation, and primitive streak anlage (PSA). We downloaded and re-analyzed the data. We first repeated the annotation of different cell types (Figure 7A), particularly the epiblast (EPI) and the epiblast-derived PSA (PSA-EPI). The EPI sample highly expresses *NANOG* and *SOX2* while PSA-EPI sample highly expresses *GATA6* and *LEF1* as expected (Figures 7B and 7C). By analyzing the differentially expressed genes and enriched signaling pathways, we found the HIPPO/YAP signaling as well as WNT and transforming growth factor β (TGF-β) pathways (Figures 7D and 7E), indicating HIPPO/YAP actively involved in early embryonic germ layers' specification. Gene set enrichment analysis (GSEA) also supported this notion (Figure 7F). In addition, since no any terms related to "cell size" was found from the Gene Ontology (GO) or GSEA database, we collected

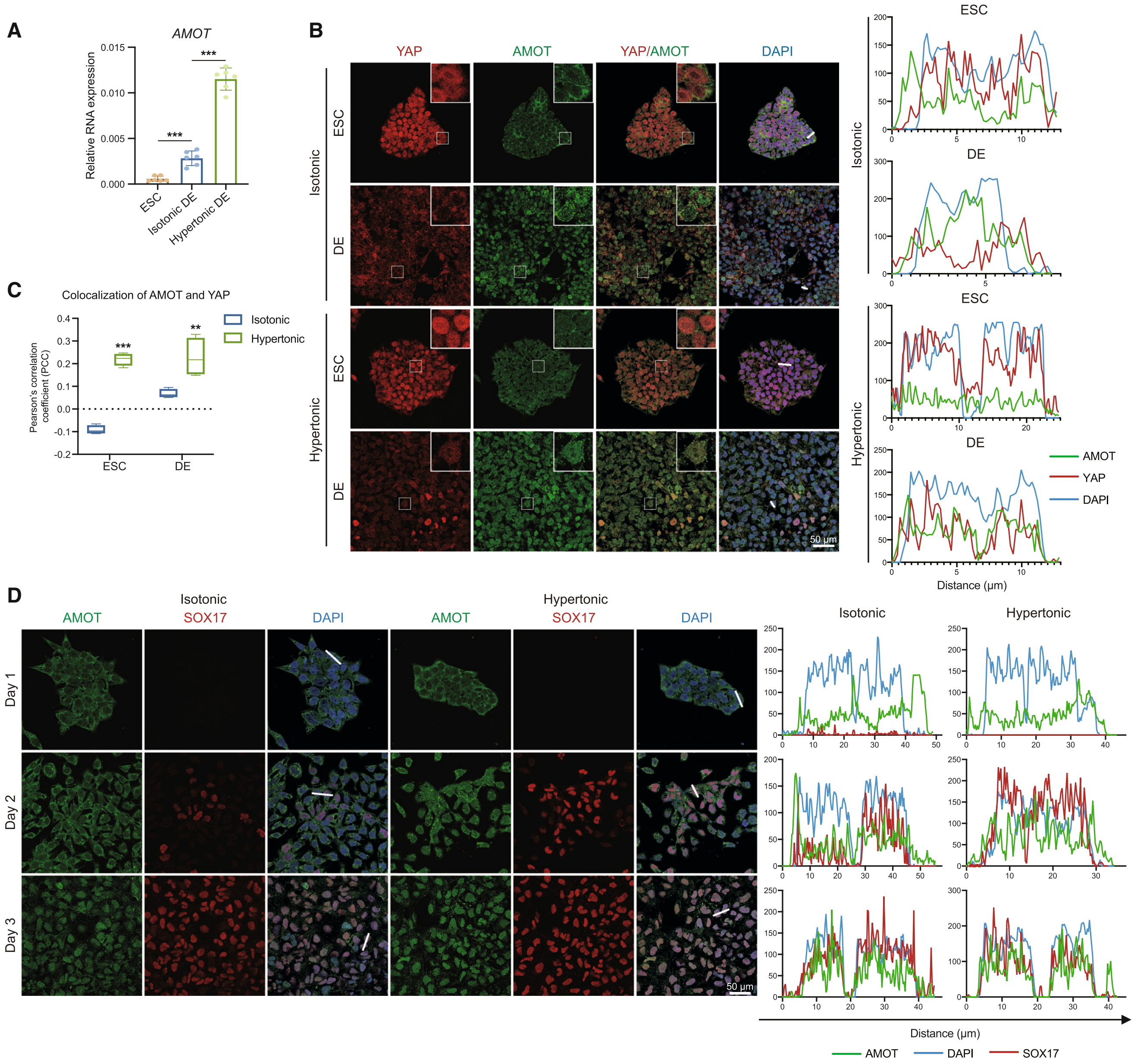

Figure 5. Cell size compression promotes the nuclear translocation of AMOT to co-localize with YAP

(A) Relative mRNA expression of *AMOT* in ESCs and DE cells under isotonic or hypertonic condition ($n = 6$ from 6 independent batches).

(B) Confocal analysis of immunofluorescent images of YAP and AMOT in isotonic or hypertonic ESCs and DE cells. Magnified views of the regions in the white boxes are provided. Representative plots of fluorescent signals intensity along the white line for YAP (red), AMOT (green), and DAPI (blue) (scale bar is 50 μm).

(C) Pearson's coefficient of YAP and AMOT proteins' co-localization.

(D) Confocal analysis of immunofluorescent images of time-course AMOT nuclear translocation in isotonic or hypertonic DE-differentiated cells. Representative plots of fluorescent signals intensity along the white line for SOX17 (red), AMOT (green), and DAPI (blue) (scale bar is 50 μm) (ns means not statistically significant, $^{**}p < 0.01$, $^{***}p < 0.001$).

another dataset about hematopoietic stem cells with different sizes (Lengefeld et al., 2021) and generated the differentially expressed genes, followed by overlapping those genes with the differentially expressed genes in our study (with hypertonic treatment, Figure 2G). We labeled the overlapped 132 genes as "cell-size-related genes," which enriches terms of intracellular signal transduction and phospholipid homeostasis (Figure 7G). We then

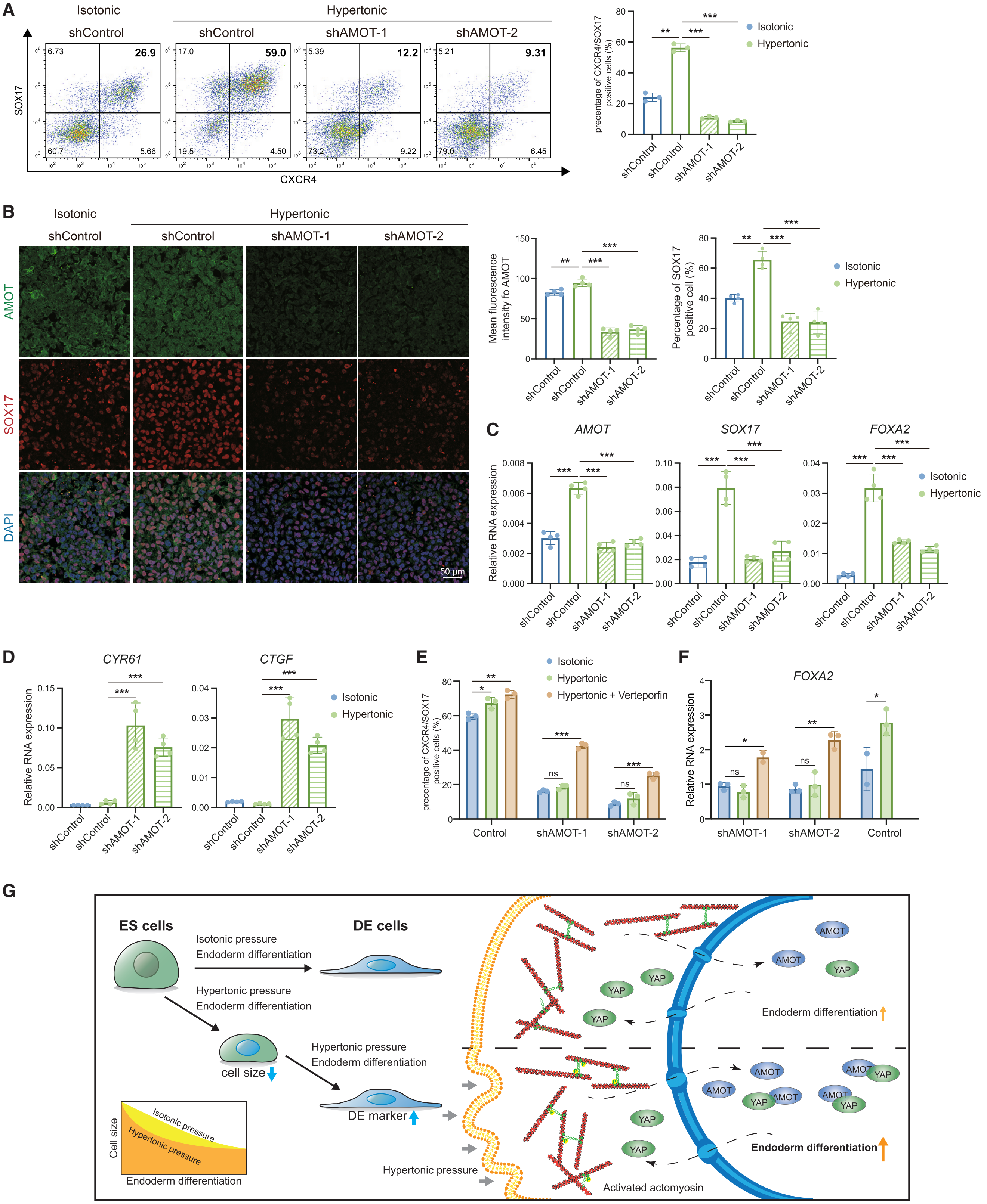

(legend on next page)

performed the GSEA and found the cell-size-related genes were significantly enriched during early development (Figure 7H). These data support that cell size and HIPPO/YAP signaling pathways actively involved in human early embryo development. Due to the ethnical and technical limitation, the emergingly developing *ex vivo* cultured human gastruloids (Liu et al., 2023; Rivron et al., 2023) would provide a choice to comprehensively explore the function of cell size control in human early development. In fact, a recent report based on the 2D-micropatterned human ESC-derived gastruloids revealed that the knockout of YAP1 could result in hyperactive NODAL signaling which retained SMAD2/3 in the nuclei, thus impeding ectoderm differentiation and enlarging mesoderm and endoderm layers (Stronati et al., 2022). Mechanistically, YAP-TEAD could block SMAD2/3 induction of mesendodermal genes by regulating binding of the NELF negative elongation factor to impair SMAD recruitment (Estarás et al., 2015); YAP knockout enabled activin to induce WNT3 expression and stabilize β-catenin, facilitating mesendodermal differentiation (Estarás et al., 2017). These reports together with our present observation provide a comprehensive understanding on early embryonic development from the angles of intracellular modulation of YAP/SMAD/WNT and extracellular stimuli including hypertonic cell size manipulation. Furthermore, there remains a lack of direct evidence regarding the potential promotion of the activin/Nodal signaling pathway due to the inhibition of YAP signaling induced by hypertonic cell volume reduction, necessitating further investigation in future studies.

DISCUSSION

Cellular scale architecture is a key characteristic of cell state, supporting the cell functionality required for proper embryonic development. Changes in cell volume could impact cell senescence, spreading, and the cell cycle. However, the extent to which cell size affects ESC behavior is not well understood. We exploit DE differentiation as a model to investigate the role of cell size in ESC fate decisions. Our study reveals that DE differentiation involves reduced cell size and increased mechanical state (Figure 1). By inducing cell size reduction through hypertonic treatment or a TRPV4 agonist, we observed significant compression of differentiated cells and enhanced endoderm fate, accompanied by upregulated expression of developmental and endoderm lineage genes (Figure 2). Furthermore, we found that myosin activity participates in hypertonic cell size diminution and promotion of endoderm fate, and a myosin inhibitor partially reverses the upregulation of genes in hypertonic DE cells (Figure 3). We discovered that DE lineage differentiation is accompanied by the suppression of YAP activity with hypertonic cell compression speeding up this process. Meanwhile, we observed partial YAP protein nuclear localization in hypertonic DE cells compared to isotonic ones (Figure 4). Then, we investigated the YAP suppressor AMOT and discovered that hypertonic pressure accelerates its nuclear localization, enhancing YAP-AMOT co-localization. The nuclear localization of AMOT depends on actomyosin and contributes to DE differentiation by suppressing YAP activity (Figure 5). These findings were corroborated by AMOT knockdown experiments, which showed that inhibiting AMOT restricts the heightened DE differentiation induced by YAP signaling (Figure 6). Thus, we propose a model in which hypertonic conditions induce cell volume reduction, promoting DE differentiation (Figure 6G). Hypertonic pressure during DE differentiation accelerates cell volume reduction primarily through actomyosin remodeling, prompting AMOT translocation to the nucleus. This sequence suppresses YAP activity via AMOT-YAP binding, ultimately facilitating DE differentiation.

To understand how hypertonic pressure promotes endoderm differentiation, we manipulated cell-size-sensitive elements (Figures 3 and S4). Piezo1, a mechanosensitive ion channel, regulates cell contractility and volume (Syeda et al., 2016; Clapham, 2007; Hua et al., 2010). FAK and Src activate the volume-regulated anion channel, crucial for cellular responses to osmotic stress (Browe and

Figure 6. AMOT contributes to the promotion effect of cell size diminution on DE differentiation by inhibiting YAP pathway
(A) Quantitative flow cytometric analysis of CXCR4 and SOX17 expression in shControl and shAMOT DE cells under hypertonic condition (n = 3 from 3 independent batches).
(B) Representative confocal images for AMOT (green) and SOX17 (red) as detected in shControl and shAMOT DE cells under hypertonic condition (scale bar is 50 μm). Quantifications of the intensity of the AMOT fluorescent signals (a.u.) and percentage of SOX17-positive cell (%), with nuclei counterstained with DAPI (blue), shown as right.
(C and D) Relative mRNA expression of *AMOT*, DE marker (C) and YAP target genes (D) in shControl and shAMOT DE cells under hypertonic condition.
(E and F) Quantitative flow cytometric analysis of CXCR4 and SOX17 expression (E) and RNA expression of DE marker gene *FOXA2* (F) in shControl and shAMOT DE cells under hypertonic condition, with or without YAP inhibitor verteporfin (n = 3 from 3 independent batches).
(G) Model of hypertonic condition promoting endoderm differentiation via actomyosin-dependent cell size diminution, and AMOT nuclear translocation, which suppresses YAP activity (ns means not statistically significant, $^{**}p < 0.01$, $^{***}p < 0.001$).

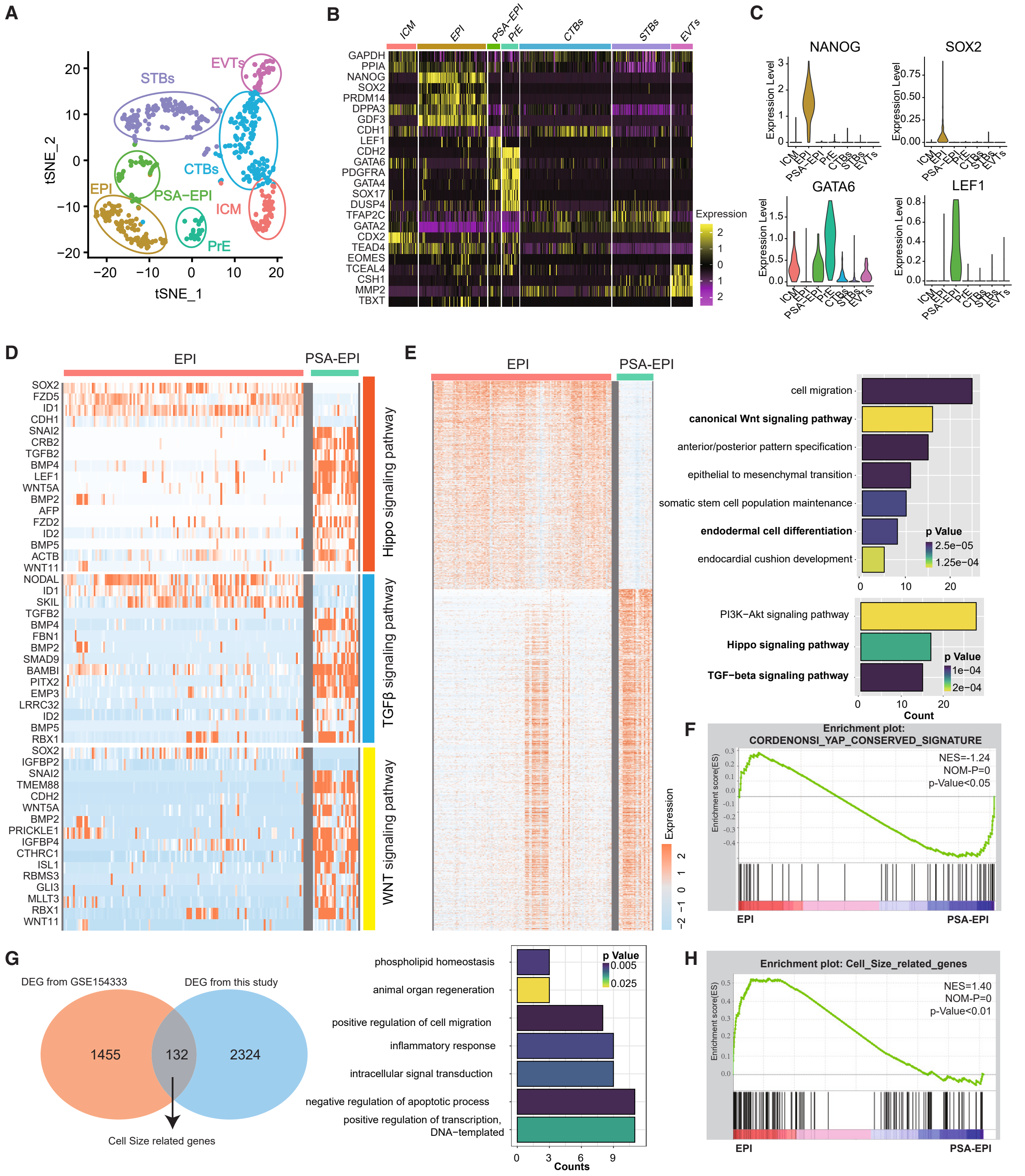

Figure 7. Cell size and YAP signal pathways likely involve in human early development

(A) t-SNE analyses revealed 8 clusters, including EPI (epiblast) and PSA-EPI (primitive streak anlage derived from epiblast).

(B) Different clusters expressed lineage-specific markers.

(C) Violin plot showing expression of marker genes for EPI and PSA-EPI.

(legend continued on next page)

Baumgarten, 2006; Hoffmann et al., 2009). As part of the cytoskeleton, the actomyosin cytoskeleton generates contractile tension through ATP hydrolysis, involving non-muscle myosin II and actin filaments (F-actin), influencing cell volume and morphology (Venkova et al., 2022; Hartman and Spudich, 2012). Remodeling of the actomyosin cytoskeleton is critical for mesoderm differentiation from pluripotent stem cells, characterized by a transition from cortical actin to stress fibers and associated cell compression (Pagliari et al., 2021). However, the functional effect of the actomyosin cytoskeleton under osmotic pressure remains unexplored. Potential explanations include the reduced spatial distance between F-actin and myosin due to altered intracellular crowding (Li et al., 2021b), or fluctuations in actomyosin cytoskeleton polymerization and depolymerization rates influenced by cytoplasmic viscosity (Molines et al., 2022). Further studies are needed to elucidate these specific mechanisms.

The nuclear localization of AMOT is regulated by its phosphorylation status; increased phosphorylation directs AMOT to the cytoplasm, while hypo-phosphorylation favors nuclear localization (Kang et al., 2020). However, understanding how hypertonic pressure or the actomyosin cytoskeleton influences AMOT's nuclear entry remains challenging. Thus, we propose another mechanism: cells establish a physical connection between nuclei and the actomyosin cytoskeleton, wherein reduced cell size flattens nuclei, stretches nuclear pores, and lowers their mechanical resistance to molecular transport (Elosegui-Artola et al., 2017). Therefore, we speculate that earlier nuclear entry of AMOT in hypertonic DE cells depends on nuclear pore stretching and sensitivity to actomyosin cytoskeleton. YAP defines the first cell fate choice in the mouse embryo and aberrant YAP expression leads to embryonic lethality (Varelas, 2014). Moreover, human pluripotent stem cells display sustained basal YAP-driven transcriptional activity, which inhibits cytoskeleton dynamics and maintains pluripotency. When germ layers are specified, the YAP-TEAD complex is promptly inactivated, and cytoskeleton remodeling can occur (Pagliari et al., 2021). AMOT inhibits YAP activity through direct binding, acting as a scaffold protein to promote YAP phosphorylation and degradation through LATS1/2 kinases (Wang et al., 2022), or leading to YAP cytoplasmic retention (Zaltsman et al., 2019). Given that AMOT can bind to YAP in the nucleus (Li and Fan, 2017), we speculate that AMOT binds to YAP and competitively inhibits YAP interaction with TEAD or promotes the binding of YAP with nuclear actin. However, the specific mechanism still needs to be further explored in subsequent studies.

In summary, we report that cell size decreases during human ESC differentiation into endoderm, with hypertonic pressure enhancing DE differentiation. The actomyosin cytoskeleton plays a critical role in reducing cell size and promoting endoderm fate under hypertonic pressure. Cell size reduction suppresses YAP activity by facilitating AMOT nuclear translocation, thereby promoting endoderm differentiation. Therefore, our study provides insights into the functional relationship between cell size, physical state, and intracellular signaling during endoderm differentiation. Therefore, our study provides valuable insights into the role of cell size, by establishing a connection between the physical state of cells and intracellular signaling.

Limitations of the study

Cell size is a complex feature that may affect numerous other aspects. During cell culture, cell size fluctuates due to factors such as cell cycle, growth, or mechanical environment. Apart from the signaling molecules mentioned in the manuscript, many other molecular changes may occur, warranting exploration in future studies. In addition, the protocol for endoderm differentiation is not optimized in different culture systems and therefore may not be generalizable. Furthermore, there is a lack of direct *in vivo* experiments to prove the conclusions of this study, necessitating further investigation in future, such as the emerging human embryoid/gastruloid system.

EXPERIMENTAL PROCEDURES

Resource availability

Lead contact

Further information and requests for resources and reagents should be directed to and will be fulfilled by the lead contact, Donghui Zhang (dongh.zhang@hubu.edu.cn) or Wei Jiang (jiangw.mri@whu.edu.cn).

Materials availability

Plasmids and cell lines generated in this study should be directed to and will be fulfilled by the lead contact.

This study did not generate new unique reagents.

(D) Heatmap showing expression levels of genes related to DE-associated signaling pathways (TGF-β and WNT as well as Hippo/YAP) between EPI and PSA-EPI.

(E) Heatmap and representative GO terms showing expression levels of differentially expressed genes (DEGs) between EPI and PSA-EPI.

(F) GSEA showing YAP signaling was enriched in DEGs between EPI and PSA-EPI.

(G) Venn diagram defining the cell-size-related genes based on GSE154333 and this study and representative enriched GO terms for this gene set.

(H) GSEA showing cell-size-related gene set was enriched in DEGs between EPI and PSA-EPI.

Data and code availability

The RNA-seq data in this study have been uploaded to the Gene Expression Omnibus (GEO) database under accession number GSE232608. The processed RNA-seq data are available in the GEO database.

All original microscopy data reported in this paper will be shared by the lead contact upon reasonable request.

Methods details

Cell culture and differentiation

Two different human ESC lines, H9 and HUES8, were used in this study. They were cultured in mTeSR1 medium (STEMCELL Technologies, #AB217641) on Matrigel-coated plates. Our work on human ESCs and iPSCs is approved by the Biomedical Ethics Committee of Wuhan University (WHU-LFMD-IRB2024026). Human embryonic kidney 293T (HEK293T) cells were cultured with DMEM containing 10% fetal bovine serum (Gibco, #10270–106) and 1% penicillin-streptomycin (Gibco, #15140163). The endoderm differentiation protocol of human ESCs was based on the previous reports (Lu et al., 2023; Yang et al., 2020; Jiang et al., 2013a) with minor modifications. IMDM (Gibco, #C12440500BT) and F12 (Gibco, #C11765500BT) mixed at a ratio of 1:1 (IMDM/F12) were used as basal medium, supplemented with 0.2% BSA (YEASEN, #36106ES76), 1% B27 (without Vitamin A, Shanghai BasalMedia Technologies, S441J7), and 1% penicillin-streptomycin and activin A (100 ng/mL, PeproTech, #120–14P) for 3 or 4 days for endoderm differentiation. DMEM could be also used as basal medium instead of IMDM/F12 and the rest ingredients were the same as aforementioned.

Hypertonic stimulation was performed by adding different amounts of polyethylene glycol 300 (PEG300) (Selleck, #S6704) or sucrose (Hushi, #10021418) to isotonic culture medium as reported (Casula et al., 2017; Li et al., 2021b). Isotonic culture medium was defined as control medium. Based on previous studies, PEG300 is a polymer preventing non-specific protein binding and, after adding PEG300 for 2 min, cell size and mechanics achieved equilibration (Guo et al., 2017; Akabayov et al., 2013). When indicated, 1 μM Yoda1 (Topscience, #T7506), 5 μM GsMTx-4 (MCE, #HY-P1410A), 1 μM PP1 (Topscience, #T6196), 1 μM PF-573228 (Topscience, #T2001), 10–100 nM jasplakinolide (Abcam, #ab141409), 0.1–1 μM latrunculin A (Cayman Chemical, #10010630), 10 μM blebbistatin (Topscience, #T6038), 2 μM ML-7 hydrochloride (MCE, #HY-15417), 0.5 nM calyculin A (MCE, #HY-18983), 2.5 μM CHIR-98014 (Selleck, #S2924), 2 μM IWR-1 (Selleck, #S7086), 10 μM Y27632 2HCl (Selleck, #S1049), 1–10 μM Lats-IN-1 (MCE, #HY-138489), 0.1–1 μM verteporfin (Topscience, #T3112), 10 μM GSK205 (MCE, # HY-120691A), 50 nM GSK101 (MCE, # HY-19608), 10 μM (E)-osmundacetone (MAPK inhibitor) (MCE, # HY-N1966), or corresponding amount of vehicle (dimethyl sulfoxide [DMSO, Sigma, #D2438]) was introduced into the endoderm differentiation medium.

Cell size measurement

For cell cross-section area and diameter statistics, after experimental treatment, cultured cells are digested into single cells and suspended in their specific culture medium. Suspended cells were visualized and photographed using microscopy (Olympus), and cross-sectioned area and diameter of cells were quantified from images using ImageJ/Fiji. Suspended cell volume was automatically measured by Cellometer Mini Automated Cell Counter (Nexcelom). For 3D confocal assay, single cells were seeded at low density in confocal culture dish. The fluorescent images were obtained by segmenting time-lapse 3D confocal stacks of cells, and the 3D cell volume was computed and reconstructed.

SUPPLEMENTAL INFORMATION

Supplemental information can be found online at https://doi.org/10.1016/j.stemcr.2024.07.001.

ACKNOWLEDGMENTS

We would like to thank Dr. Hongyun Li and Dr. Wenbin Zhang at Wuhan University for technical help and helpful discussion and Dr. Xi Ren at Carnegie Mellon University and Dr. Linfeng Huang at Kunshan Duke University for insightful discussion and editorial support. We thank the core facility of the Medical Research Institute at Wuhan University for the technical support. This work was supported by the National Key R&D Program of China (2021YFA1101902), the National Natural Science Foundation of China (no. 32350019, 32270857, and 32171107), the Fundamental Research Funds for the Central Universities China (2042022dx0003), and the Open Funds of Hubei Key Laboratory of Embryonic Stem Cell Research (Hubei University of Medicine) (ESOF2023003).

AUTHOR CONTRIBUTIONS

W.J., D.Z., and L.J. conceived the project and designed the experiment. L.J. performed most of the bench experiments. Z.L. performed the biomechanics test. C.Y. analyzed the sequencing data and L.Z. re-analyzed the single-cell RNA-seq data (GSE136447). Y.Y. provided support in cell culture. L.J. drafted the manuscript, and W.J. and L.J. finalized the manuscript. All authors contributed to and approved the final manuscript.

DECLARATION OF INTERESTS

The authors declare no competing interests.

Received: May 30, 2024
Revised: June 29, 2024
Accepted: July 1, 2024
Published: August 1, 2024

REFERENCES

Akabayov, B., Akabayov, S.R., Lee, S.J., Wagner, G., and Richardson, C.C. (2013). Impact of macromolecular crowding on DNA replication. Nat. Commun. *4*, 1615.

Auffret, J., Freemark, M., Carré, N., Mathieu, Y., Tourrel-Cuzin, C., Lombès, M., Movassat, J., and Binart, N. (2013). Defective prolactin signaling impairs pancreatic β-cell development during the perinatal period. Am. J. Physiol. Endocrinol. Metab. *305*, E1309–E1318.

Baek, J., Lopez, P.A., Lee, S., Kim, T.S., Kumar, S., and Schaffer, D.V. (2022). Egr1 is a 3D matrix-specific mediator of mechanosensitive stem cell lineage commitment. Sci. Adv. *8*, eabm4646.

Browe, D.M., and Baumgarten, C.M. (2006). EGFR kinase regulates volume-sensitive chloride current elicited by integrin stretch via PI-3K and NADPH oxidase in ventricular myocytes. J. Gen. Physiol. *127*, 237–251.

Cadart, C., Venkova, L., Piel, M., and Cosentino Lagomarsino, M. (2022). Volume growth in animal cells is cell cycle dependent and shows additive fluctuations. Elife *11*, e70816.

Casula, E., Asuni, G.P., Sogos, V., Fadda, S., Delogu, F., and Cincotti, A. (2017). Osmotic behaviour of human mesenchymal stem cells: Implications for cryopreservation. PLoS One *12*, e0184180.

Chan, C.J., Costanzo, M., Ruiz-Herrero, T., Mönke, G., Petrie, R.J., Bergert, M., Diz-Muñoz, A., Mahadevan, L., and Hiiragi, T. (2019). Hydraulic control of mammalian embryo size and cell fate. Nature *571*, 112–116.

Chang, L., Azzolin, L., Di Biagio, D., Zanconato, F., Battilana, G., Lucon Xiccato, R., Aragona, M., Giulitti, S., Panciera, T., Gandin, A., et al. (2018). The SWI/SNF complex is a mechanoregulated inhibitor of YAP and TAZ. Nature *563*, 265–269.

Clapham, D.E. (2007). Calcium Signaling. Cell *131*, 1047–1058.

Courtois, A., Schuh, M., Ellenberg, J., and Hiiragi, T. (2012). The transition from meiotic to mitotic spindle assembly is gradual during early mammalian development. J. Cell Biol. *198*, 357–370.

Elosegui-Artola, A., Andreu, I., Beedle, A.E.M., Lezamiz, A., Uroz, M., Kosmalska, A.J., Oria, R., Kechagia, J.Z., Rico-Lastres, P., Le Roux, A.L., et al. (2017). Force Triggers YAP Nuclear Entry by Regulating Transport across Nuclear Pores. Cell *171*, 1397–1410.

Estarás, C., Benner, C., and Jones, K.A. (2015). SMADs and YAP compete to control elongation of β-catenin:LEF-1-recruited RNAPII during hESC differentiation. Mol. Cell *58*, 780–793.

Estarás, C., Hsu, H.T., Huang, L., and Jones, K.A. (2017). YAP repression of the WNT3 gene controls hESC differentiation along the cardiac mesoderm lineage. Genes Dev. *31*, 2250–2263.

Estevez, A.Y., Bond, T., and Strange, K. (2001). Regulation of I(Cl,swell) in neuroblastoma cells by G protein signaling pathways. Am. J. Physiol. Cell Physiol. *281*, C89–C98.

Fang, X.Z., Zhou, T., Xu, J.Q., Wang, Y.X., Sun, M.M., He, Y.J., Pan, S.W., Xiong, W., Peng, Z.K., Gao, X.H., and Shang, Y. (2021). Structure, kinetic properties and biological function of mechanosensitive Piezo channels. Cell Biosci. *11*, 13.

Fingar, D.C., Salama, S., Tsou, C., Harlow, E., and Blenis, J. (2002). Mammalian cell size is controlled by mTOR and its downstream targets S6K1 and 4EBP1/eIF4E. Genes Dev. *16*, 1472–1487.

Guo, M., Pegoraro, A.F., Mao, A., Zhou, E.H., Arany, P.R., Han, Y., Burnette, D.T., Jensen, M.H., Kasza, K.E., Moore, J.R., et al. (2017). Cell volume change through water efflux impacts cell stiffness and stem cell fate. Proc. Natl. Acad. Sci. USA *114*. E8618–e8627.

Hartman, M.A., and Spudich, J.A. (2012). The myosin superfamily at a glance. J. Cell Sci. *125*, 1627–1632.

Hoffmann, E.K., Lambert, I.H., and Pedersen, S.F. (2009). Physiology of cell volume regulation in vertebrates. Physiol. Rev. *89*, 193–277.

Horner, V.L., and Wolfner, M.F. (2008). Mechanical stimulation by osmotic and hydrostatic pressure activates Drosophila oocytes in vitro in a calcium-dependent manner. Dev. Biol. *316*, 100–109.

Hua, S.Z., Gottlieb, P.A., Heo, J., and Sachs, F. (2010). A mechanosensitive ion channel regulating cell volume. Am. J. Physiol. Cell Physiol. *298*, C1424–C1430.

Jiang, W., Liu, Y., Liu, R., Zhang, K., and Zhang, Y. (2015). The lncRNA DEANR1 facilitates human endoderm differentiation by activating FOXA2 expression. Cell Rep. *11*, 137–148.

Jiang, W., Wang, J., and Zhang, Y. (2013a). Histone H3K27me3 demethylases KDM6A and KDM6B modulate definitive endoderm differentiation from human ESCs by regulating WNT signaling pathway. Cell Res. *23*, 122–130.

Jiang, W., Zhang, D., Bursac, N., and Zhang, Y. (2013b). WNT3 is a biomarker capable of predicting the definitive endoderm differentiation potential of hESCs. Stem Cell Rep. *1*, 46–52.

Jo, A.O., Ryskamp, D.A., Phuong, T.T., Verkman, A.S., Yarishkin, O., MacAulay, N., and Križaj, D. (2015). TRPV4 and AQP4 Channels Synergistically Regulate Cell Volume and Calcium Homeostasis in Retinal Müller Glia. J. Neurosci. *35*, 13525–13537.

Kang, P.H., Schaffer, D.V., and Kumar, S. (2020). Angiomotin links ROCK and YAP signaling in mechanosensitive differentiation of neural stem cells. Mol. Biol. Cell *31*, 386–396.

Koushki, N., Ghagre, A., Srivastava, L.K., Molter, C., and Ehrlicher, A.J. (2023). Nuclear compression regulates YAP spatiotemporal fluctuations in living cells. Proc. Natl. Acad. Sci. USA *120*, e2301285120.

Lau, H.H., Amirruddin, N.S., Loo, L.S.W., Chan, J.W., Iich, E., Krishnan, V.G., Hoon, S., and Teo, A.K.K. (2023). FGFR-mediated ERK1/2 signaling contributes to mesendoderm and definitive endoderm formation in vitro. iScience *26*, 107265.

Lee, H.P., Stowers, R., and Chaudhuri, O. (2019). Volume expansion and TRPV4 activation regulate stem cell fate in three-dimensional microenvironments. Nat. Commun. *10*, 529.

Lengefeld, J., Cheng, C.W., Maretich, P., Blair, M., Hagen, H., McReynolds, M.R., Sullivan, E., Majors, K., Roberts, C., Kang, J.H., et al. (2021). Cell size is a determinant of stem cell potential during aging. Sci. Adv. *7*, eabk0271.

Li, H., Zhang, C., Hu, Y., Liu, P., Sun, F., Chen, W., Zhang, X., Ma, J., Wang, W., Wang, L., et al. (2021a). A reversible shearing DNA probe for visualizing mechanically strong receptors in living cells. Nat. Cell Biol. *23*, 642–651.

Li, L., and Fan, C.M. (2017). A CREB-MPP7-AMOT Regulatory Axis Controls Muscle Stem Cell Expansion and Self-Renewal Competence. Cell Rep. *21*, 1253–1266.

Li, Y., Chen, M., Hu, J., Sheng, R., Lin, Q., He, X., and Guo, M. (2021b). Volumetric Compression Induces Intracellular Crowding to Control Intestinal Organoid Growth via Wnt/β-Catenin Signaling. Cell Stem Cell *28*, 170–172.

Liu, A., Yu, T., Young, K., Stone, N., Hanasoge, S., Kirby, T.J., Varadarajan, V., Colonna, N., Liu, J., Raj, A., et al. (2020). Cell Mechanical and Physiological Behavior in the Regime of Rapid Mechanical Compressions that Lead to Cell Volume Change. Small *16*, e1903857.

Liu, L., Oura, S., Markham, Z., Hamilton, J.N., Skory, R.M., Li, L., Sakurai, M., Wang, L., Pinzon-Arteaga, C.A., Plachta, N., et al. (2023). Modeling post-implantation stages of human development into early organogenesis with stem-cell-derived peri-gastruloids. Cell *186*, 3776–3792.

Loh, K.M., Ang, L.T., Zhang, J., Kumar, V., Ang, J., Auyeong, J.Q., Lee, K.L., Choo, S.H., Lim, C.Y., Nichane, M., et al. (2014). Efficient endoderm induction from human pluripotent stem cells by logically directing signals controlling lineage bifurcations. Cell Stem Cell *14*, 237–252.

Lu, P., Yang, J., Li, M., Wen, S., Zhang, T., Yan, C., Liu, R., Xiao, Y., Wang, X., and Jiang, W. (2023). A desert lncRNA HIDEN regulates human endoderm differentiation via interacting with IMP1 and stabilizing FZD5 mRNA. Genome Biol. *24*, 92.

Lv, H., Li, L., Sun, M., Zhang, Y., Chen, L., Rong, Y., and Li, Y. (2015). Mechanism of regulation of stem cell differentiation by matrix stiffness. Stem Cell Res. Ther. *6*, 103.

Molines, A.T., Lemière, J., Gazzola, M., Steinmark, I.E., Edrington, C.H., Hsu, C.T., Real-Calderon, P., Suhling, K., Goshima, G., Holt, L.J., et al. (2022). Physical properties of the cytoplasm modulate the rates of microtubule polymerization and depolymerization. Dev. Cell *57*, 466–479.

Nakajima, H., Yamamoto, K., Agarwala, S., Terai, K., Fukui, H., Fukuhara, S., Ando, K., Miyazaki, T., Yokota, Y., Schmelzer, E., et al. (2017). Flow-Dependent Endothelial YAP Regulation Contributes to Vessel Maintenance. Dev. Cell *40*, 523–536.

Nishioka, N., Inoue, K., Adachi, K., Kiyonari, H., Ota, M., Ralston, A., Yabuta, N., Hirahara, S., Stephenson, R.O., Ogonuki, N., et al. (2009). The Hippo signaling pathway components Lats and Yap pattern Tead4 activity to distinguish mouse trophectoderm from inner cell mass. Dev. Cell *16*, 398–410.

Pagliari, S., Vinarsky, V., Martino, F., Perestrelo, A.R., Oliver De La Cruz, J., Caluori, G., Vrbsky, J., Mozetic, P., Pompeiano, A., Zancla, A., et al. (2021). YAP-TEAD1 control of cytoskeleton dynamics and intracellular tension guides human pluripotent stem cell mesoderm specification. Cell Death Differ. *28*, 1193–1207.

Pentinmikko, N., Lozano, R., Scharaw, S., Andersson, S., Englund, J.I., Castillo-Azofeifa, D., Gallagher, A., Broberg, M., Song, K.Y., Sola Carvajal, A., et al. (2022). Cellular shape reinforces niche to stem cell signaling in the small intestine. Sci. Adv. *8*, eabm1847.

Perez Gonzalez, N., Tao, J., Rochman, N.D., Vig, D., Chiu, E., Wirtz, D., and Sun, S.X. (2018). Cell tension and mechanical regulation of cell volume. Mol. Biol. Cell *29*, 2591–2600.

Perez-Gonzalez, N.A., Rochman, N.D., Yao, K., Tao, J., Le, M.T., Flanary, S., Sablich, L., Toler, B., Crentsil, E., Takaesu, F., et al. (2019). YAP and TAZ regulate cell volume. J. Cell Biol. *218*, 3472–3488.

Price, C.J., Stavish, D., Gokhale, P.J., Stevenson, B.A., Sargeant, S., Lacey, J., Rodriguez, T.A., and Barbaric, I. (2021). Genetically variant human pluripotent stem cells selectively eliminate wild-type counterparts through YAP-mediated cell competition. Dev. Cell *56*, 2455–2470.

Rivron, N.C., Martinez Arias, A., Pera, M.F., Moris, N., and M'Hamdi, H.,I. (2023). An ethical framework for human embryology with embryo models. Cell *186*, 3548–3557.

Roffay, C., Molinard, G., Kim, K., Urbanska, M., Andrade, V., Barbarasa, V., Nowak, P., Mercier, V., García-Calvo, J., Matile, S., et al. (2021). Passive coupling of membrane tension and cell volume during active response of cells to osmosis. Proc. Natl. Acad. Sci. USA *118*, e2103228118.

Rosowski, K.A., Mertz, A.F., Norcross, S., Dufresne, E.R., and Horsley, V. (2015). Edges of human embryonic stem cell colonies display distinct mechanical properties and differentiation potential. Sci. Rep. *5*, 14218.

Smejkal, J., Aubrecht, P., Semerádtová, A., Štofik, M., Liegertová, M., and Malý, J. (2023). Immunocapturing rare cells from blood: A simple and robust microsystem approach. Biosens. Bioelectron. *227*, 115155.

Sordella, R., Classon, M., Hu, K.Q., Matheson, S.F., Brouns, M.R., Fine, B., Zhang, L., Takami, H., Yamada, Y., and Settleman, J. (2002). Modulation of CREB activity by the Rho GTPase regulates cell and organism size during mouse embryonic development. Dev. Cell *2*, 553–565.

Stronati, E., Giraldez, S., Huang, L., Abraham, E., McGuire, G.R., Hsu, H.T., Jones, K.A., and Estarás, C. (2022). YAP1 regulates the self-organized fate patterning of hESC-derived gastruloids. Stem Cell Rep. *17*, 211–220.

Sun, X., Ren, Z., Cun, Y., Zhao, C., Huang, X., Zhou, J., Hu, R., Su, X., Ji, L., Li, P., et al. (2020). Hippo-YAP signaling controls lineage differentiation of mouse embryonic stem cells through modulating the formation of super-enhancers. Nucleic Acids Res. *48*, 7182–7196.

Syeda, R., Florendo, M.N., Cox, C.D., Kefauver, J.M., Santos, J.S., Martinac, B., and Patapoutian, A. (2016). Piezo1 Channels Are Inherently Mechanosensitive. Cell Rep. *17*, 1739–1746.

Taglietti, V., Angelini, G., Mura, G., Bonfanti, C., Caruso, E., Monteverde, S., Le Carrou, G., Tajbakhsh, S., Relaix, F., and Messina, G. (2018). RhoA and ERK signalling regulate the expression of the transcription factor Nfix in myogenic cells. Development *145*, dev163956.

Tan, M., Jiang, L., Li, Y., and Jiang, W. (2019). Dual Inhibition of BMP and WNT Signals Promotes Pancreatic Differentiation from Human Pluripotent Stem Cells. Stem Cells Int. *2019*, 5026793.

Thomson, J.A., Itskovitz-Eldor, J., Shapiro, S.S., Waknitz, M.A., Swiergiel, J.J., Marshall, V.S., and Jones, J.M. (1998). Embryonic stem cell lines derived from human blastocysts. Science *282*, 1145–1147.

Tilly, B.C., Edixhoven, M.J., Tertoolen, L.G., Morii, N., Saitoh, Y., Narumiya, S., and de Jonge, H.R. (1996). Activation of the osmo-sensitive chloride conductance involves P21rho and is accompanied by a transient reorganization of the F-actin cytoskeleton. Mol. Biol. Cell *7*, 1419–1427.

Varelas, X. (2014). The Hippo pathway effectors TAZ and YAP in development, homeostasis and disease. Development *141*, 1614–1626.

Venkova, L., Vishen, A.S., Lembo, S., Srivastava, N., Duchamp, B., Ruppel, A., Williart, A., Vassilopoulos, S., Deslys, A., Garcia Arcos, J.M., et al. (2022). A mechano-osmotic feedback couples cell volume to the rate of cell deformation. Elife *11*, e72381.

Wang, L., Choi, K., Su, T., Li, B., Wu, X., Zhang, R., Driskill, J.H., Li, H., Lei, H., Guo, P., et al. (2022). Multiphase coalescence mediates Hippo pathway activation. Cell *185*, 4376–4393.

Wang, W., Li, N., Li, X., Tran, M.K., Han, X., and Chen, J. (2015). Tankyrase Inhibitors Target YAP by Stabilizing Angiomotin Family Proteins. Cell Rep. *13*, 524–532.

Wells, J.M., and Melton, D.A. (1999). Vertebrate endoderm development. Annu. Rev. Cell Dev. Biol. *15*, 393–410.

Xiang, L., Yin, Y., Zheng, Y., Ma, Y., Li, Y., Zhao, Z., Guo, J., Ai, Z., Niu, Y., Duan, K., et al. (2020). A developmental landscape of 3D-cultured human pre-gastrulation embryos. Nature *577*, 537–542.

Yang, J., Lu, P., Li, M., Yan, C., Zhang, T., and Jiang, W. (2020). GATA6-AS1 Regulates GATA6 Expression to Modulate Human Endoderm Differentiation. Stem Cell Rep. *15*, 694–705.

Yap, C., Goh, H.N., Familari, M., Rathjen, P.D., and Rathjen, J. (2014). The formation of proximal and distal definitive endoderm populations in culture requires p38 MAPK activity. J. Cell Sci. *127*, 2204–2216.

Yoshikawa, T., Wu, J., Otsuka, M., Kishikawa, T., Ohno, M., Shibata, C., Takata, A., Han, F., Kang, Y.J., Chen, C.Y., et al. (2015). ROCK inhibition enhances microRNA function by promoting deadenylation of targeted mRNAs via increasing PAIP2 expression. Nucleic Acids Res. *43*, 7577–7589.

Zaltsman, Y., Masuko, S., Bensen, J.J., and Kiessling, L.L. (2019). Angiomotin Regulates YAP Localization during Neural Differentiation of Human Pluripotent Stem Cells. Stem Cell Rep. *12*, 869–877.

Stem Cell Reports, Volume *19*

Supplemental Information

Cell size regulates human endoderm specification through actomyo-sin-dependent AMOT-YAP signaling

Lai Jiang, Chenchao Yan, Ying Yi, Lihang Zhu, Zheng Liu, Donghui Zhang, and Wei Jiang

Supplemental Information

Cell size regulates human endoderm specification through actomyosin-dependent AMOT-YAP signaling

Lai Jiang, Chenchao Yan, Lihang Zhu, Zheng Liu, Donghui Zhang, Wei Jiang

Figure S1. Differentiation induces changes of cell volume and mechanical state.

Figure S2. Hypertonic pressure induces changes of cell volume and enhances human endoderm differentiation.

Figure S3. Cell size diminution promotes endodermal lineage differentiation.

Figure S4. Cytoskeletal plays a role in endoderm differentiation boost caused by cell size decrease.

Figure S5. Identification of cell size related signal pathway that contributes to hypertonic endoderm differentiation.

Figure S6. Cell size compression promotes AMOT nuclear translocation.

Figure S7. AMOT is not essential for pluripotency but influential for YAP activation.

Tables S1. Primer list.

Figure S1

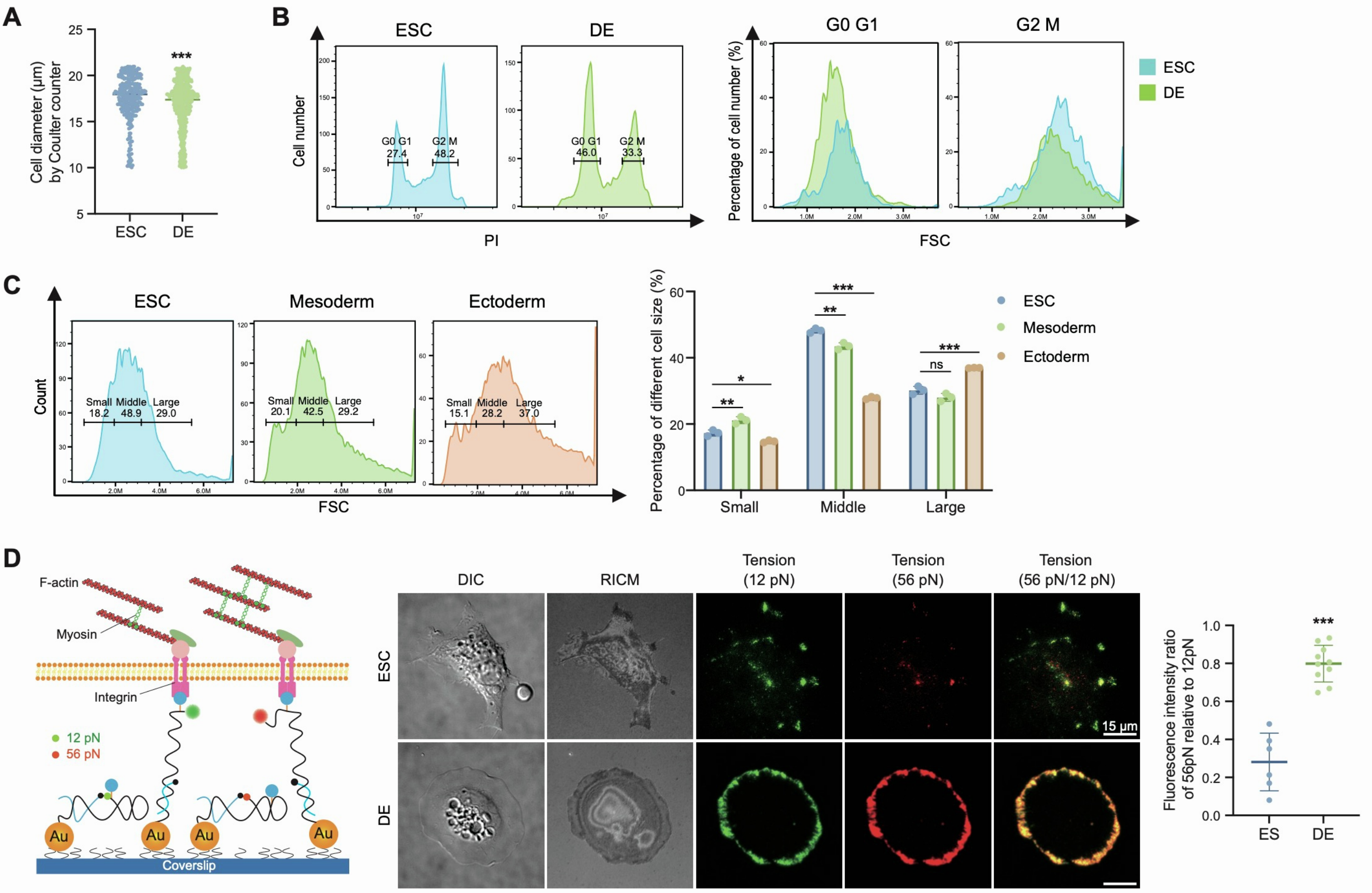
A
Cell diameter (μm) by Coulter counter
***
ESC
DE
B
ESC
DE
G0 G1
27.4
G2 M
48.2
G0 G1
46.0
G2 M
33.3
Cell number
PI
G0 G1
G2 M
ESC
DE
Percentage of cell number (%)
FSC
C
ESC
Mesoderm
Ectoderm
Count
Small 18.2
Middle 48.9
Large 29.0
Small 20.1
Middle 42.5
Large 29.2
Small 15.1
Middle 28.2
Large 37.0
FSC
Percentage of different cell size (%)
Small
Middle
Large
ns
ESC
Mesoderm
Ectoderm
D
F-actin
Myosin
Integrin
12 pN
56 pN
Au
Coverslip
DIC
RICM
Tension (12 pN)
Tension (56 pN)
Tension (56 pN/12 pN)
ESC
DE
15 μm
Fluorescence intensity ratio of 56pN relative to 12pN
ES
DE
***

Figure S1. Differentiation induces changes of cell volume and mechanical state.

(A) Quantification of cell diameter by Coulter counter (n > 330 cells from 3 independent batches per group analyzed).

(B) Flow cytometric analysis of cell cycle (left) and the corresponding size distribution (right) of ESCs and DE cells as determined by FSC.

(C) Flow cytometric analysis of cell size of ESCs, differentiated mesodermal and ectodermal cells. Size distribution of cells as determined by forward scatter (FSC).

(D) Schematic illustrating the interaction between cells and the probe-labeled surface (left). Representative microscopy images of differential interference contrast microscopy (DIC), reflection interference contrast microscopy (RICM, which can reflect the cell adherent area), and total internal reflection fluorescence (TIRF) microscopy of ESCs and DE cells that were seeded on 12pN and 56pN DNA probe surface[21] (middle, scale bar is 15 μm). Statistic total fluorescent intensities and 56pN/12pN fluorescent intensities ratio (right, n > 6 cells per group analyzed).

Figure S2

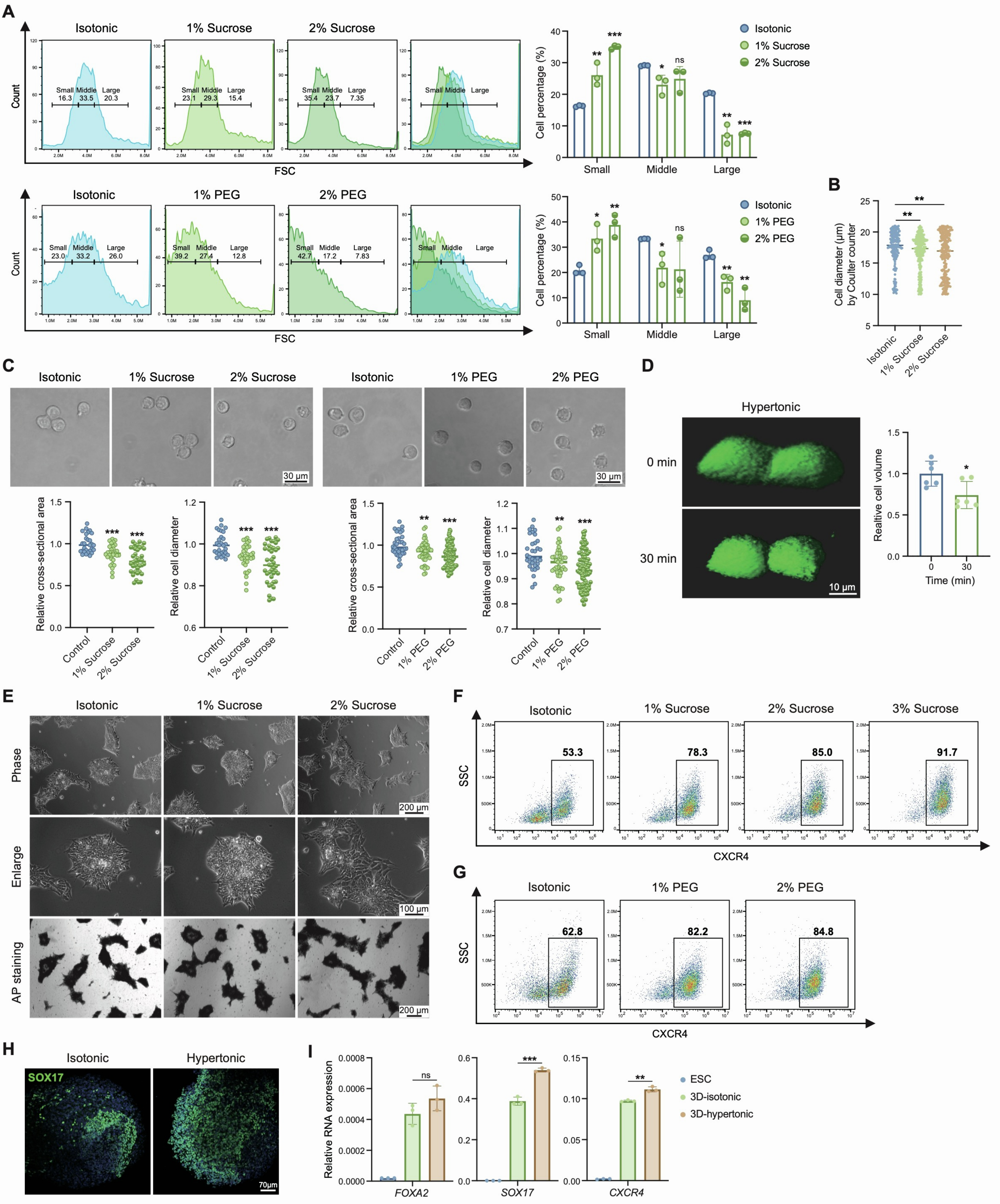
A
Isotonic
1% Sucrose
2% Sucrose
Count
FSC
Small Middle Large
16.3 33.5 20.3
23.1 29.3 15.4
35.4 23.7 7.35
Cell percentage (%)
Small
Middle
Large
1% PEG
2% PEG
23.0 33.2 26.0
39.2 27.4 12.8
42.7 17.2 7.83
B
Cell diameter (μm) by Coulter counter
C
30 μm
Relative cross-sectional area
Relative cell diameter
Control
D
Hypertonic
0 min
30 min
10 μm
Realtive cell volume
Time (min)
E
Phase
Enlarge
AP staining
200 μm
100 μm
F
3% Sucrose
SSC
CXCR4
53.3
78.3
85.0
91.7
G
62.8
82.2
84.8
H
SOX17
70μm
I
Relative RNA expression
FOXA2
SOX17
CXCR4
ESC
3D-isotonic
3D-hypertonic

Figure S2. Hypertonic pressure induces changes of cell volume and enhances human endoderm differentiation.

(A) Flow cytometric analysis showing ESCs volume decreases with the increasing concentration of sucrose or PEG300, after hypertonic pressure treatment for 48 hours. Size distribution of ESCs as determined by FSC. Gates of small, middle and large cell size and quantification of gates percentage are indicated (n = 3 from 3 independent batches).

(B) Quantification of cell diameter after hypertonic pressure treatment for 48 hours by Coulter counter (n > 190 cells from 3 independent batches per group analyzed).

(C) Representative images of ESCs and quantification of their cross-sectional area and diameter (n > 28 cells from 3 independent batches per group analyzed, scale bar is 30 μm).

(D) 3D images of ESCs after hypertonic pressure and quantification of cell volume (n = 6). Scale bar is 10 μm.

(E) Phase-contrast images of ESCs colonies and bright-field images showing the AP staining of ESCs which has been cultured in isotonic or hypertonic culture medium for 48 hours.

(F-G) Dosage-dependent promoting effect of sucrose (F) or PEG (G) for DE differentiation, measured by flow cytomeric analysis of CXCX4 expression.

(H-I) Immunostaining of SOX17 and RNA expression of DE marker genes under isotonic or hypertonic DE differentiation condition in suspension (3D).

(ns means not statistically significant, *P < 0.05, **P < 0.01, ***P < 0.001).

Figure S3

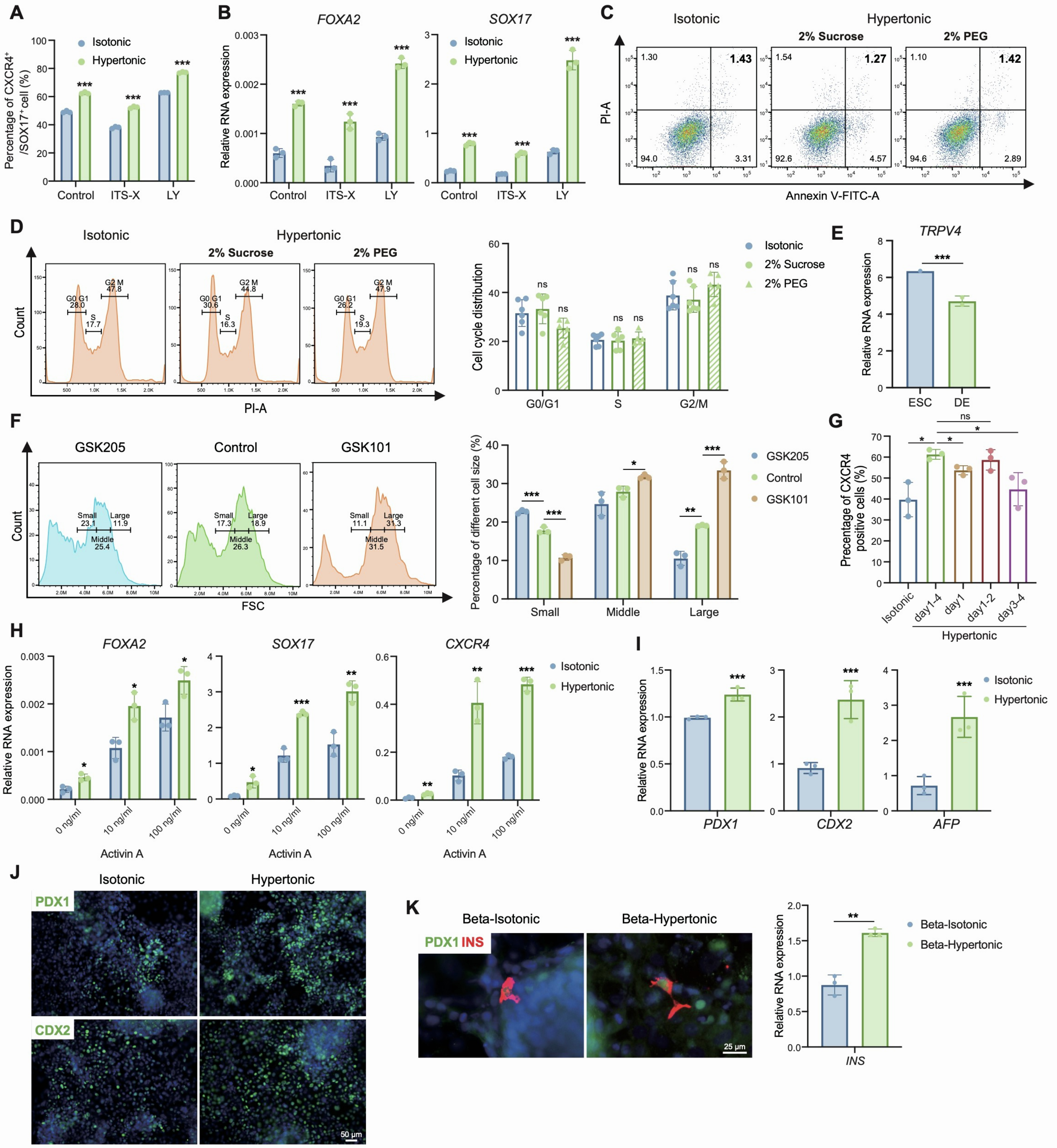
A
Isotonic
Hypertonic
Percentage of CXCR4+ /SOX17+ cell (%)
Control
ITS-X
LY
B
FOXA2
SOX17
Relative RNA expression
C
Isotonic
Hypertonic
2% Sucrose
2% PEG
PI-A
Annexin V-FITC-A
D
Count
PI-A
Cell cycle distribution
G0/G1
S
G2/M
E
TRPV4
ESC
DE
F
GSK205
Control
GSK101
Count
FSC
Percentage of different cell size (%)
Small
Middle
Large
G
Precentage of CXCR4 positive cells (%)
Isotonic
day1-4
day1
day1-2
day3-4
Hypertonic
H
FOXA2
SOX17
CXCR4
Activin A
I
PDX1
CDX2
AFP
J
Isotonic
Hypertonic
PDX1
CDX2
50 μm
K
Beta-Isotonic
Beta-Hypertonic
PDX1 INS
25 μm
INS

Figure S3. Cell size diminution promotes endodermal lineage differentiation.

(A-B) DE differentiation efficiency determined by flow cytometric analysis of CXCR4 and SOX17 expression (A) and RNA expression of DE marker genes (B) in DE cells under isotonic or hypertonic pressure, treated with PI3K inhibitor LY294002 or agonist ITS-X.

(C-D) Annexin V/PI assay (C) and cell cycle analysis using PI staining (D) in isotonic or hypertonic differentiated DE cells ($n > 5$ from 5 independent batches). The quantification of different cell cycle phases was calculated in FlowJo.

(E) RNA expression of *TRPV4* in ESCs and DE cells, determined by RNA-seq.

(F) Flow cytometric analysis showing cell size distribution treated with TRPV4 agonist or antagonist, as determined by FSC. Gates of small, middle and large cell size and quantification of gates percentage are indicated ($n = 3$ from 3 independent batches).

(G) The quantification of the proportion of CXCR4-positive cells assessed by flow cytometry, under hypertonic treatment added in different days.

(H) Hypotonic condition reduced the demand for Activin A during DE differentiation.

(I-J) RNA expression (I) and immunostaining analysis (J) of pancreatic (PDX1), intestinal (CDX2), and hepatic (AFP) lineages after further differentiating hypotonic DE cells.

(K) RNA expression and immunostaining analysis of pancreatic beta cells (Insulin, INS) differentiated from hypertonic DE cells.

(ns means not statistically significant, $^{*}P < 0.05$, $^{**}P < 0.01$, $^{***}P < 0.001$)

Figure S4

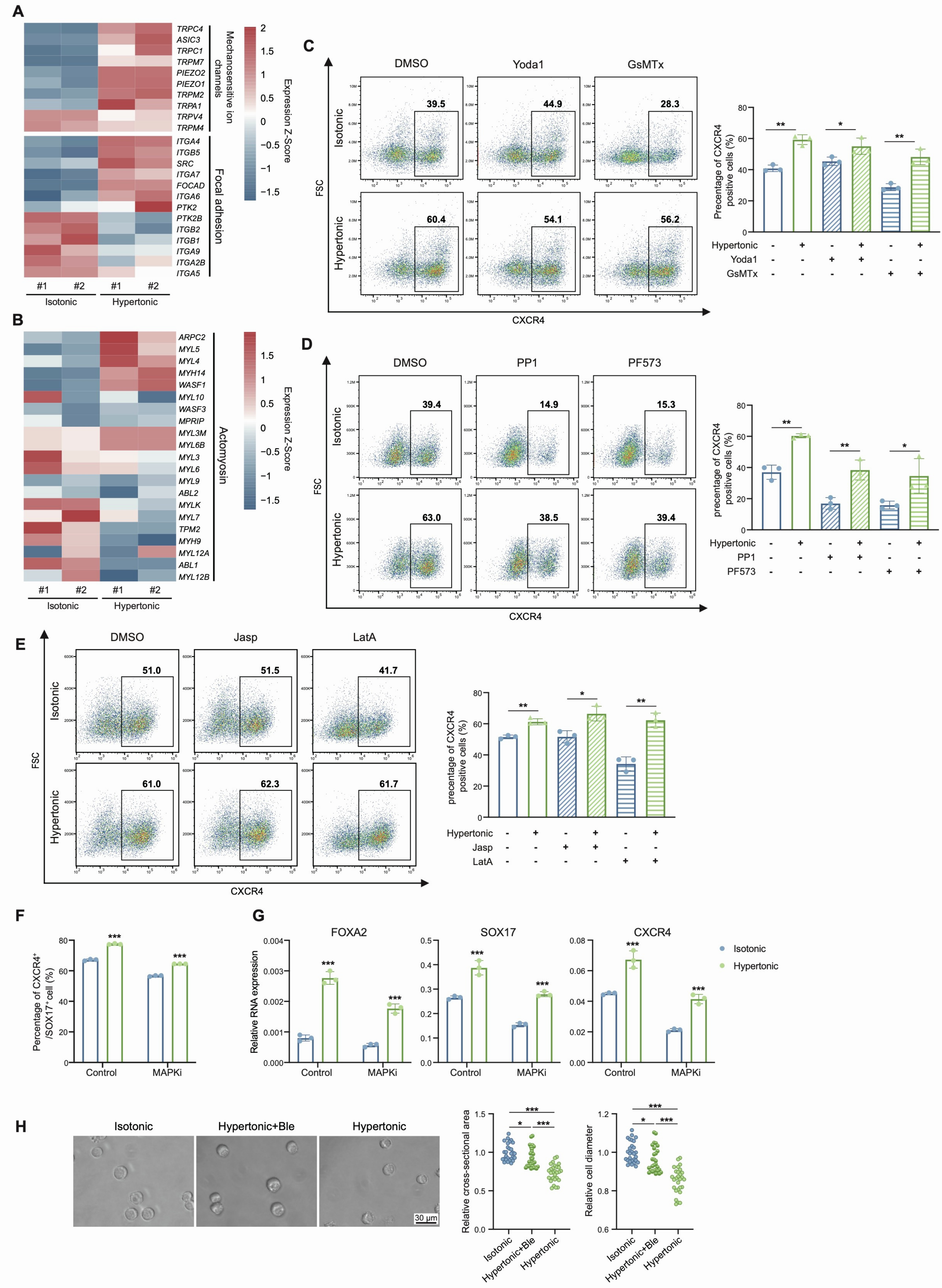
A
TRPC4
ASIC3
TRPC1
TRPM7
PIEZO2
PIEZO1
TRPM2
TRPA1
TRPV4
TRPM4
Mechanosensitive ion channels
ITGA4
ITGB5
SRC
ITGA7
FOCAD
ITGA6
PTK2
PTK2B
ITGB2
ITGB1
ITGA9
ITGA2B
ITGA5
Focal adhesion
Expression Z-Score
2
1.5
1
0.5
0
−0.5
−1
−1.5
#1
#2
#1
#2
Isotonic
Hypertonic
B
ARPC2
MYL5
MYL4
MYH14
WASF1
MYL10
WASF3
MPRIP
MYL3M
MYL6B
MYL3
MYL6
MYL9
ABL2
MYLK
MYL7
TPM2
MYH9
MYL12A
ABL1
MYL12B
Actomyosin
Expression Z-Score
1.5
1
0.5
0
−0.5
−1
−1.5
#1
#2
#1
#2
Isotonic
Hypertonic
C
DMSO
Yoda1
GsMTx
Isotonic
Hypertonic
FSC
CXCR4
39.5
44.9
28.3
60.4
54.1
56.2
Precentage of CXCR4 positive cells (%)
80
60
40
20
0
**
*
**
Hypertonic - + - + - +
Yoda1 - - + + - -
GsMTx - - - - + +
D
DMSO
PP1
PF573
Isotonic
Hypertonic
FSC
CXCR4
39.4
14.9
15.3
63.0
38.5
39.4
precentage of CXCR4 positive cells (%)
80
60
40
20
0
**
**
*
Hypertonic - + - + - +
PP1 - - + + - -
PF573 - - - - + +
E
DMSO
Jasp
LatA
Isotonic
Hypertonic
FSC
CXCR4
51.0
51.5
41.7
61.0
62.3
61.7
precentage of CXCR4 positive cells (%)
80
60
40
20
0
**
*
**
Hypertonic - + - + - +
Jasp - - + + - -
LatA - - - - + +
F
Percentage of CXCR4+ /SOX17+ cell (%)
80
60
40
20
0
***
***
Control
MAPKi
G
FOXA2
SOX17
CXCR4
Relative RNA expression
0.004
0.003
0.002
0.001
0.000
0.5
0.4
0.3
0.2
0.1
0.0
0.08
0.06
0.04
0.02
0.00
***
Control
MAPKi
Isotonic
Hypertonic
H
Isotonic
Hypertonic+Ble
Hypertonic
30 μm
Relative cross-sectional area
1.5
1.0
0.5
0.0
Relative cell diameter
1.2
1.0
0.8
0.6
***
*
Isotonic
Hypertonic+Ble
Hypertonic

Figure S4. Cytoskeletal plays a role in endoderm differentiation boost caused by cell size decrease.

(A-B) Heatmap showing the expression of mechanosensitive ion channels, focal adhesion-related genes (A) and actomyosin cytoskeleton-related genes (B) within isotonic and hypertonic DE cells.

(C-E) Flow cytometry of CXCR4 expression in DE cells under isotonic or hypertonic culture differentiation condition with or without drug stimulations and quantification of CXCR4-positive cells proportion (n=3 from 3 independent batches). (C) 1μM Yoda1 (Piezo1 agonist) and 5μM GsMTx-4 (Piezo1 inhibitor), (D) 1μM PP1 (Src-family kinases inhibitor) and 1μM PF-573228 (focal adhesion kinase inhibitor), (E) 10nM Jasplakinolide (actin polymerization agonist) and 100nM Latrunculin A (actin polymerization inhibitor).

(F-G) DE differentiation efficiency determined by flow cytometric analysis of CXCR4 and SOX17 expression (F) and RNA expression of DE marker genes (G) in DE cells under isotonic or hypertonic pressure, treated with MAPK inhibitor.

(H) Representative images of ESCs in isotonic condition, hypertonic condition with or without Ble addition for 48h, and quantification of their cross-sectional area and diameter. ($n > 28$ from 3 independent batches cells per group analyzed, scale bar is 30 μm).

(ns means not statistically significant, $*P < 0.05$, $**P < 0.01$, $***P < 0.001$).

Figure S5

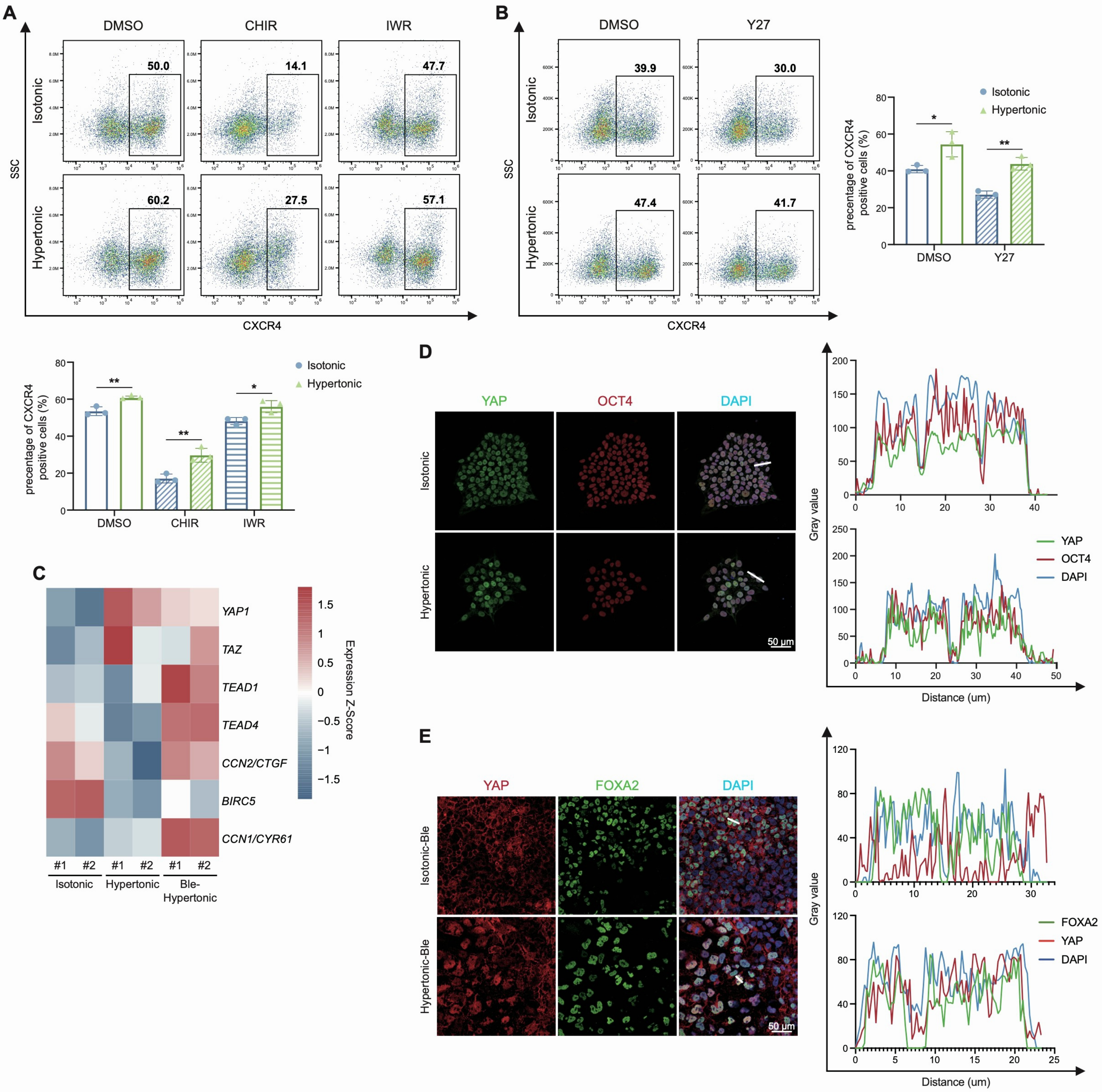

A
DMSO
CHIR
IWR
Isotonic
Hypertonic
SSC
CXCR4
50.0
14.1
47.7
60.2
27.5
57.1
precentage of CXCR4 positive cells (%)
Isotonic
Hypertonic
DMSO
CHIR
IWR
B
DMSO
Y27
39.9
30.0
47.4
41.7
precentage of CXCR4 positive cells (%)
C
YAP1
TAZ
TEAD1
TEAD4
CCN2/CTGF
BIRC5
CCN1/CYR61
Expression Z-Score
#1
#2
Isotonic
Hypertonic
Ble-Hypertonic
D
YAP
OCT4
DAPI
50 μm
Gray value
Distance (um)
E
YAP
FOXA2
DAPI
Isotonic-Ble
Hypertonic-Ble

Figure S5. Identification of cell size related signal pathway that contributes to hypertonic endoderm differentiation.

(A-B) Flow cytometric analysis of CXCR4 expression in DE cells under isotonic or hypertonic condition with or without drug stimulations and quantification of CXCR4-positive cells proportion as below (A) or right (B) (n=3 from 3 independent batches). (A) 2.5μM CHIR-98014 (WNT agonist) and 2μM IWR-1 (WNT inhibitor). (B) 10μM Y27632-2HCl (ROCK inhibitor).

(C) Heatmap showing the expression of YAP signaling-related genes in DE cells under isotonic or hypertonic condition with or without myosin inhibitor.

(D) Immunofluorescent labelling images of OCT4 and YAP in ESCs under hypertonic condition observed by confocal microscopy, with representative plots of fluorescent signals intensity along the white line for YAP (green), OCT4 (red) and DAPI (blue) (scale bar is 50 μm).

(E) Immunofluorescent labelling images of FOXA2 and YAP in DE cells under isotonic or hypertonic condition with Ble stimulations observed by confocal microscopy, with representative plots of fluorescent signals intensity along the white line for FOXA2 (green), YAP (red) and DAPI (blue) (scale bar is 50 μm).

(ns means not statistically significant, $*P < 0.05$, $**P < 0.01$)

Figure S6

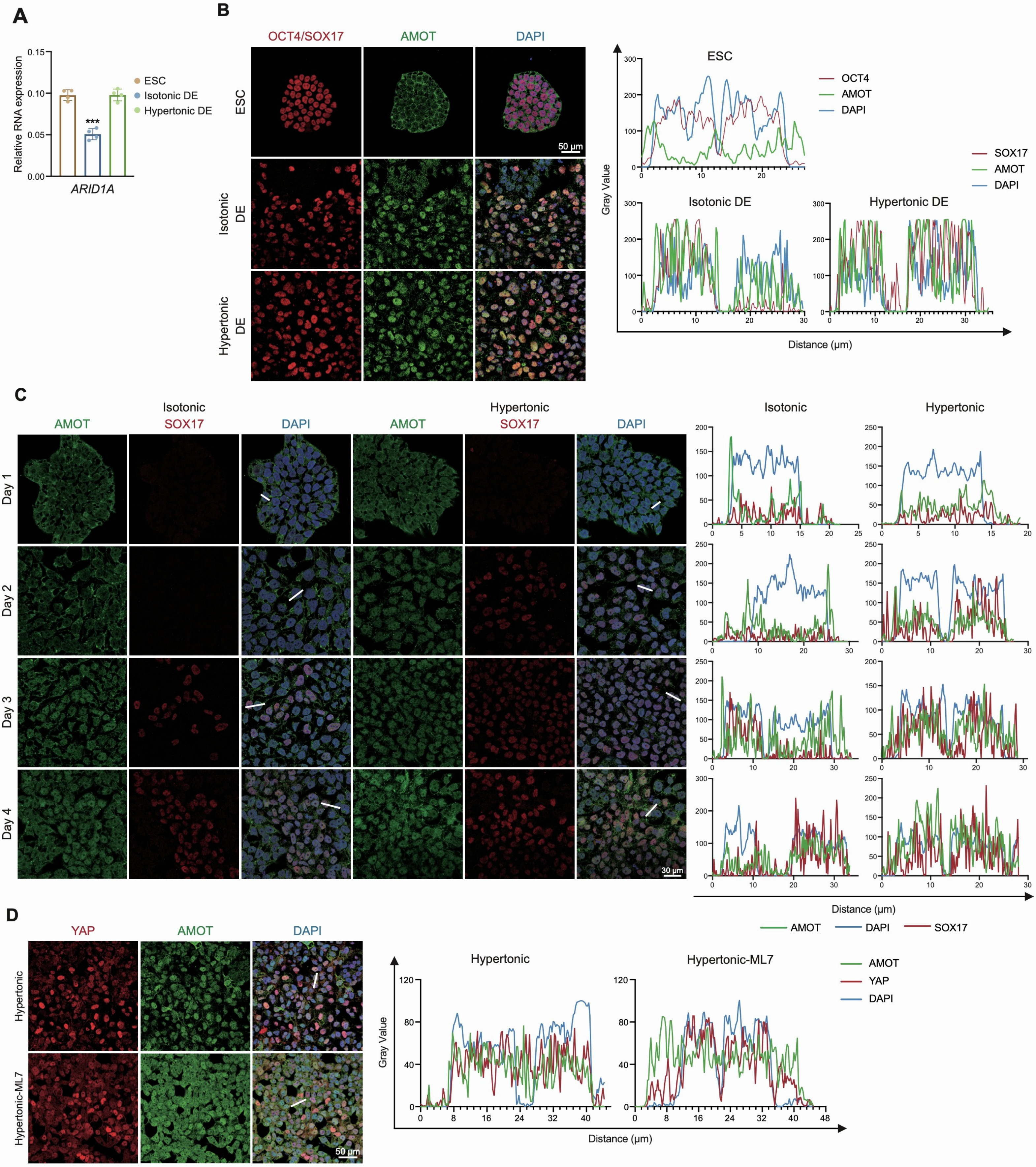
A
Relative RNA expression
0.15
0.10
0.05
0.00
***
ARID1A
ESC
Isotonic DE
Hypertonic DE
B
OCT4/SOX17
AMOT
DAPI
ESC
Isotonic DE
Hypertonic DE
50 μm
ESC
OCT4
AMOT
DAPI
SOX17
AMOT
DAPI
Isotonic DE
Hypertonic DE
Gray Value
Distance (μm)
C
Isotonic
Hypertonic
AMOT
SOX17
DAPI
Day 1
Day 2
Day 3
Day 4
30 μm
Isotonic
Hypertonic
Distance (μm)
AMOT
DAPI
SOX17
D
YAP
AMOT
DAPI
Hypertonic
Hypertonic-ML7
50 μm
Hypertonic
Hypertonic-ML7
AMOT
YAP
DAPI
Gray Value
Distance (μm)

Figure S6. Cell size compression promotes AMOT nuclear translocation.

(A) Relative mRNA expression of *ARID1A* in ESCs and DE cells under isotonic or hypertonic condition (n=4 from 4 independent batches). (***$P < 0.001$).

(B-D) Immunofluorescent labelling images were observed by confocal microscopy. Representative plots of fluorescent signals intensity along the white line. (B) AMOT nuclear translocation in ESCs and DE cells under isotonic or hypertonic condition (scale bar is 50 μm). (C) Time course AMOT nuclear translocation in H9 DE cells under isotonic or hypertonic condition (scale bar is 30 μm). (D) AMOT's co-localization with YAP and nuclear translocation in DE cells under hypertonic condition with or without myosin inhibition (scale bar is 50 μm).

Figure S7

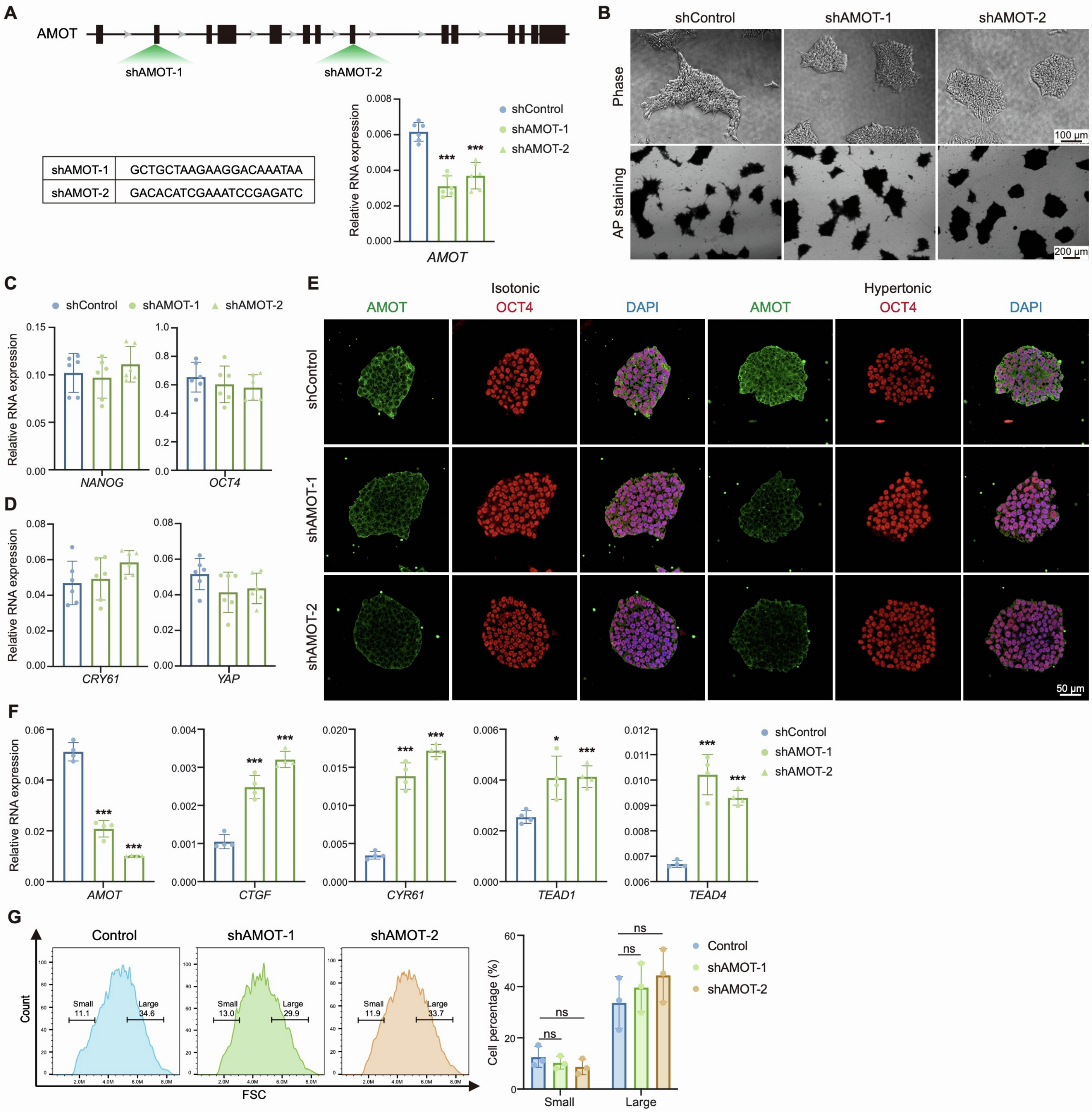

A
AMOT
shAMOT-1
shAMOT-2
shAMOT-1 GCTGCTAAGAAGGACAAATAA
shAMOT-2 GACACATCGAAATCCGAGATC
Relative RNA expression
AMOT
shControl
shAMOT-1
shAMOT-2
***
B
shControl
shAMOT-1
shAMOT-2
Phase
AP staining
100 μm
200 μm
C
NANOG
OCT4
D
CRY61
YAP
E
Isotonic
Hypertonic
AMOT
OCT4
DAPI
shControl
shAMOT-1
shAMOT-2
50 μm
F
AMOT
CTGF
CYR61
TEAD1
TEAD4
G
Control
shAMOT-1
shAMOT-2
Count
FSC
Small
Large
Cell percentage (%)
ns

Figure S7. AMOT is not essential for pluripotency but influential for YAP activation.

(A) Illustration of designed shRNA target position and the measurement of knockdown efficiency of shControl and shAMOT in ESCs (n=6 from 6 independent batches).

(B) Phase-contrast images of shControl, shAMOT-1, shAMOT-2 ESCs colonies and bright-field images showing the AP staining of ESCs which has been cultured in isotonic or hypertonic medium for 48 hours (scale bar is 100 μm and 200 μm, respectively).

(C-D) Relative mRNA expression of pluripotency gene (C) and YAP target genes (D) in shControl and shAMOT ESCs (n=6 from 6 independent batches).

(E) Representative confocal images for AMOT (green) and OCT4 (red) as detected in shControl and shAMOT ESCs under hypertonic condition. Nuclei were counterstained with DAPI (blue) (scale bar is 50 μm).

(F) Relative mRNA expression of YAP target genes in shControl and shAMOT 293T.

(G) Cell size distribution of AMOT knockdown and control cells, determined by FSC.

(ns means not statistically significant, $*P < 0.05$, $**P < 0.01$, $***P < 0.001$).

Supplementary Tables S1. Primer list.

Gene	Forward primer	Reverse primer
OCT4	CAAAGCAGAAACCCTCGTGC	TCTCACTCGGTTCTCGATACTG
SOX2	GTCATTTGCTGTGGGTGATG	AGAAAAACGAGGGAAATGGG
NANOG	CCCCAGCCTTTACTCTTCCTA	CCAGGTTGAATTGTTCCAGGTC
FOXA2	GGAGCAGCTACTATGCAGAGC	CGTGTTCATGCCGTTCATCC
SOX17	GCATGACTCCGGTGTGAATCT	TCACACGTCAGGATAGTTGCAGT
CXCR4	TACACCGAGGAAATGGGCTCA	AGATGATGGAGTAGATGGTGGG
T	GATGATCGTGACCAAGAACGG	CCACGAAGTCCAGCAGGAA
MIXL1	GAGACTTGGCACGCCTGT	GGTACCCCGACATCCACTT
AMOT	AGGGCGAGATTCGGAGGAT	CCTCTGACCCCTCATATTCCTT
ARID1A	TTATCTCCGCGTCAGCCTTC	ACTGGGGTAGTTGGCATTGG
YAP1	AAGAACTGCTTCGGCAGGTG	GCAGGGCTAACTCCTGACAT
TAZ	TCCCAGCCAAATCTCGTGATG	AGCGCATTGGGCATACTCAT
TEAD1	ATGGAAAGGATGAGTGACTCTGC	TCCCACATGGTGGATAGATAGC
TEAD4	CCGGTCGGAACGAGCTG	CCTTAGCTGCCTGGTCCTTT
CTGF	AGGAGTGGGTGTGTGACGA	CCAGGCAGTTGGCTCTAATC
CYR61	ACCGCTCTGAAGGGGATCT	ACTGATGTTTACAGTTGGGCTG
GAPDH	AATGAAGGGGTCATTGATGG	AAGGTGAAGGTCGGAGTCAA

EXPERIMENTAL PROCEDURES

METHODS DETAILS

Flow cytometric analysis

For endoderm differentiation analysis, the cultured cells were digested into single cells by TrypLE (Gibco, #12604021) for 3 minutes at 37°C and washed twice with DPBS containing 2% FBS. Then cells were incubated with 1:200 dilution of CD184 (CXCR4) -APC (BD, #555976) for 30 minutes, followed with intracellular antibody staining. Cells were fixed according to the manufacturer's instructions of Transcription Factor Buffer Set (BD, #562574) and then incubated with SOX17-Alexa488 (BD, #562205) antibody. Corresponding isotype was used as control. The SOX17-positive or CXCR4-positive cells were detected by NovoCyte flow cytometer (ACEA, USA) and analyzed by FlowJo software. For cell cycle analysis, a cell cycle detection kit (Keygen, China, Cat#KGA512) was used according to the protocol included in the kit. DE cells were dissociated into single cells and fixed with chilled anhydrous ethanol for 1 hour at room temperature. After washed with phosphate-buffered saline (PBS) twice, cells were resuspended with 500μL RNase A solution (100μg/ml RNase A) and incubated at 37 °C for 30 minutes. Then the cells were added with propidium iodide (PI) solution (50μg/ml PI) and incubated at room temperature for another 30 minutes in dark. Finally, a NovoCyte flow cytometer (ACEA, USA) was used to analyze these cells. The DNA content was analyzed on the basis of PI intensity in the FlowJo (v10.4.0), by which these sorted cells were divided into each cell cycle phase (subG1, G1, S, and G2/M) and calculated the proportion of each phase. For apoptosis analysis, an Annexin V-FITC/PI apoptosis detection kit (Keygen, China, Cat#KGA106) was used. DE cells were dissociated into single cells and added with Annexin V-FITC/PI, followed with incubation at room temperature for 15 minutes in dark. Cell apoptosis should be assessed by flow cytometer within 1 hour and later analyzed by FlowJo.

Immunofluorescence assay and alkaline phosphatase (AP) staining

For immunofluorescence assay, cells were fixed in 4% paraformaldehyde after PBS washing and blocked with blocking solution (DPBS with 10% (v/v) donkey serum and 0.3% Triton-100). Then cells were incubated overnight with the primary antibodies at proper concentration at 4 °C. The used primary antibodies in the experiment were: SOX17 (R&D, #AF1924, 1:200), FOXA2 (R&D, #AF2400, 1:200), OCT4 (CST, #2750, 1:200), NANOG (CST, #4903, 1:200), YAP1 (Abclonal, #A19134, 1:200), YAP1 (Abcam, # ab205270, 1:200), AMOT (Proteintech, #24550-1-AP, 1:200), Phospho-Myosin Light Chain 2 (Thr18/Ser19) Antibody (CST, #3674), Myosin IIa Antibody (CST, #3403). After washed with PBS for three times, cells were stained with secondary antibodies at room temperature in dark, then counterstained with 5 μg/ml 4',6-Diamidino-2-phenylindole dihydrochloride (DAPI, Sigma, Cat#10236276001) for 10 minutes. Cells were visualized and imaged using fluorescence microscopy (Olympus) or confocal microscope (Leica, Stellaris 5 WLL). Image analysis and image processing were performed using ImageJ/Fiji (NIH)(Schindelin et al., 2012). Co-localization was evaluated using the ImageJ to calculate Pearson's correlation or the RGB profiles tool to obtain the fluorescence intensity plots. For AP staining, the ESCs were cultured for at least 48 hours. The medium was discarded, and the ESC samples were washed three times with PBS, following AP staining experiments were performed according to manufacturer's recommendations (Beyotime, Cat#C3206). Briefly, cultured cells were fixed with 4% paraformaldehyde for 20 minutes, followed with washing three times with PBS and incubation in BCIP/NBT staining mix in dark for 3 hours. After staining reaction termination by PBS washing, colonies were visualized and photographed using microscopy (Olympus).

RNA extraction and RT-qPCR

Total RNA from cells was extracted with HiPure Total RNA Mini Kit (Magen, Cat#R4111-03) according to manufacturer's manual. 1 mg of total RNA was used for reverse-transcription to cDNA with the ABScript II RT Master Mix (ABclonal, Cat#RK20402). RT-qPCR was performed on a C1000 Touch Thermal Cycler machine

(Bio-Rad) using 2x SYBR Green qPCR Master Mix (Biomake, Cat#B21203). The relative gene expression levels were normalized to the level of *GAPDH* based on the delta Ct method. All RT-qPCR experiments were carried out at least three replicates. Comparison between samples was performed using Student's t test. The primers used in the RT-qPCR assays are listed in Table S1.

RNA-seq and data analysis

RNA was isolated with HiPure Total RNA Mini Kit and sent to Geekgene (Beijing, China) for RNA-seq library construction and sequencing on an Illumina Hiseq X Ten platform with paired-end reads. For data analysis, RNA-seq raw data that contained adapters were removed and trimmed by Trim Galore (v0.6.6). The clean reads were aligned to the human GRCh38 genome reference with the HISAT2 (v2.1.0), and gene expression counts were determined by featureCounts (v2.0.1). All counts were further normalized with TPM (Transcripts Per Million) in R software. The RNA-seq data of ESC and DE cells in Figure 1 were derived from our previous works(Lu et al., 2023; Yang et al., 2020; Zheng et al., 2021). To determine differential expression genes, DESeq2 (v1.30.1)(Love et al., 2014) was used to define significant differences genes by setting adjust p value <0.05 and abs (log_2(fold-change)) > 1.0. Gene Ontology analysis was executed by Profiler(Reimand et al., 2019) (https://biit.cs.ut.ee/gprofiler/gost), Venn diagram was made by Venn website (https://bioinformatics.psb.ugent.be/webtools/Venn/) and the heatmap was made by pheatmap (v1.0.12).

AMOT knockdown

We utilized shRNA for knockdown and the shRNAs specifically against *AMOT* and scramble control were cloned into lentiviral vector pLKO.1 plasmid (Addgene Plasmid 10878). For lentivirus packaging, the lentiviral shRNA plasmid and lentiviral helping vectors (psPAX2, pMD2.G) were transfected in HEK293T cells and the virus was concentrated. After lentivirus infection to human ESCs, 2 mg/mL puromycin was used for selection to establish stable shAMOT knockdown ESC line. The sequences of

oligoes were listed as Fig. S7A and below: shControl: GAAGTATTCCGCGTACGTT.

Statistical analysis

Statistical analysis was conducted using PRISM 9.5.1 for macOS. The results were shown as means ± SD from at least three independent experiments. Single comparison between two groups was analyzed by two-tailed unpaired t-test. Comparisons between multiple groups were determined using One-Way ANOVA analysis. Pearson's correlation analysis was used to evaluate the correlation between two variables. P value<0.05 is considered statistically significant (* means $p < 0.05$, ** means $p < 0.01$, and *** means $p < 0.001$), while "n.s." stands for not statistically significant.
